# RAGE mediates LPS-induced inflammatory pain in human skin – a double-blind, randomised, placebo-controlled full crossover study

**DOI:** 10.1038/s41467-026-76771-2

**Published:** 2026-09-02

**Authors:** Felix J. Resch, Sibylle Pramhas, Bernd Jilma, Sabine Sator, Stefan Heber, Michael J. M. Fischer

**Affiliations:** 1 Center for Physiology and Pharmacology, Medical University of Vienna, Vienna, Austria; 2 Division of Special Anesthesia and Pain Medicine, Department of Anesthesia, Intensive Care and Pain Medicine, Medical University of Vienna, Vienna, Austria; 3 Department of Clinical Pharmacology, Medical University of Vienna, Vienna, Austria

**Keywords:** Molecular medicine, Neurophysiology, Pattern recognition receptors, Experimental models of disease

## Abstract

Inflammatory pain is a major clinical challenge, yet appropriate human models mimicking local infection are lacking. We established a human pain model based on intradermal administration of lipopolysaccharide, a component of Gram-negative bacteria, and investigated the time course and underlying molecular mechanisms of inflammatory pain hypersensitivity. A placebo-controlled pilot study with 12 healthy subjects showed that intradermal lipopolysaccharide injection induces hyperaemia peaking at 4.5 h and mechanical hypersensitivity peaking at 6 h. Hypersensitivity to increasingly acidic injections and mechanical pinch lasts longer than hyperaemia. The double-blind, randomised, placebo-controlled full crossover main study (NCT07092215) included 40 healthy subjects and investigated the role of the Receptor for Advanced Glycation End-products. The primary hypothesis was: ‘The blood flow is different in lipopolysaccharide-injected skin spots compared to lipopolysaccharide-injected skin spots where azeliragon is co-injected’. The two secondary hypotheses were: ‘Acid-induced pain is different in lipopolysaccharide-injected skin spots compared to lipopolysaccharide-injected skin spots where azeliragon is co-injected’ and ‘Mechanical pinch-induced pain is different in lipopolysaccharide-injected skin spots compared to lipopolysaccharide-injected skin spots where azeliragon is co-injected’. Co-injection of the respective antagonist azeliragon largely reduces lipopolysaccharide-induced hyperaemia (−87%) and significantly attenuates hypersensitivity to mechanical (−55%) and increasingly acidic stimuli (−40%). In contrast, the Toll-like receptor 4 antagonist resatorvid has no effect on these readouts. In both naïve and inflamed skin, the TRPV1 antagonist BCTC inhibits the majority of acid-induced pain. Lipopolysaccharide-induced inflammation causes a substantial shift in the pH sensitivity of pain, suggesting that even mild tissue acidification contributes to inflammatory pain. The human lipopolysaccharide skin inflammation model depends largely on the Receptor for Advanced Glycation End-products, highlighting its potential as a target in inflammation.

## Abstract (web summary)

Bacterial lipopolysaccharide induces inflammation. Inflammatory pain is a major clinical challenge lacking suitable and viable human pain models. Here the authors present a double-blind, randomised, placebo controlled full crossover clinical trial examining the pain response to LPS challenge in human skin and suggest a role for RAGE in the LPS-induced inflammatory pain response.

## Introduction

Most painful conditions involve inflammation. This is reflected in human models of skin inflammation, including UV-B exposure^1^, injection of the proinflammatory mediators NGF^2^ or bradykinin^3^, and thermal injury by freeze^4^ or burn^5^, which also have an inflammatory component. However, there are currently no human pain models mimicking local infection. Intravenous administration of Lipopolysaccharide (LPS), a highly proinflammatory component of gram-negative bacteria, is an established human model of systemic inflammation^6^. LPS has so far also been applied intradermally in humans in four studies^7–10^. It results in small-diameter inflamed skin spots, which appear suitable for establishing a pain model.

In cellular as well as in animal models, LPS has been shown to sensitise, which is generally thought to be mediated by Toll-like receptor 4 (TLR4)^11,12^. Aside from TLR4 activation, other receptors might contribute to the LPS-induced inflammation. A potential role of the receptor for advanced glycation end-products (RAGE) pathway has been suggested^13,14^. RAGE is a multiligand receptor activated by endogenous danger-associated molecular patterns such as High Mobility Group Box 1 (HMGB1), which is released during tissue injury and inflammation^15,16^. In the skin, RAGE is expressed by keratinocytes, fibroblasts, immune cells, and sensory neurons, positioning it as a potential modulator of both immune and nociceptive processes^17^. LPS exposure triggering RAGE signalling has been directly linked to sensory neuron sensitisation^14^. TLR4- and RAGE-dependent pathways converge on shared downstream mechanisms, including NF-κB activation and oxidative stress. Modulation of the combined RAGE/TLR4 signalling axis attenuated mechanical and thermal hypersensitivity in models of inflammatory pain^18^. This study makes use of the RAGE antagonist azeliragon, which has been investigated in three phase III clinical trials, and the TLR4 antagonist resatorvid to clarify the relative importance of these receptors in LPS-induced human skin inflammation.

Inflammation is associated with tissue acidification^19^. Sensory neurons express several ion channels, which have been shown to be sensitised or activated by acidic pH, including transient receptor potential cation channel subfamily V member 1 (TRPV1)^20^ and subfamily A member 1 (TRPA1)^21^, or acid-sensing ion channels (ASICs)^22^. In healthy human skin, TRPV1 is responsible for the majority of acid-induced pain^23^. Inflammatory mediators, like ATP, PGE_2_, bradykinin or NGF sensitise proton activation of TRPV1^24^, which suggests a relevant role of this ion channel in inflammation. Acid-induced pain has not been investigated in inflammation. It is unclear whether TRPV1 continues to be the primary acid sensor in inflamed human skin. The objectives of this study were (1) to establish LPS-based skin inflammation as a human pain model, (2) to elucidate the underlying pathway leading to inflammatory pain hypersensitivity and (3) to clarify the role of TRPV1 in acid-induced pain in LPS-based skin inflammation. To address objective 1, a pilot study involving 12 healthy subjects was conducted to measure the time course of LPS-induced change in blood flow and pain sensitivity. Mechanical pinch, contact heat and intradermal injection of acidic fluid were used as noxious stimuli. Based on these data, 40 healthy subjects completed the main study to address objectives 2 by application of antagonists and objective 3 by application of a TRPV1 antagonist.

## Results

### Time course of intradermal LPS-induced inflammation

**Fig. 1:**
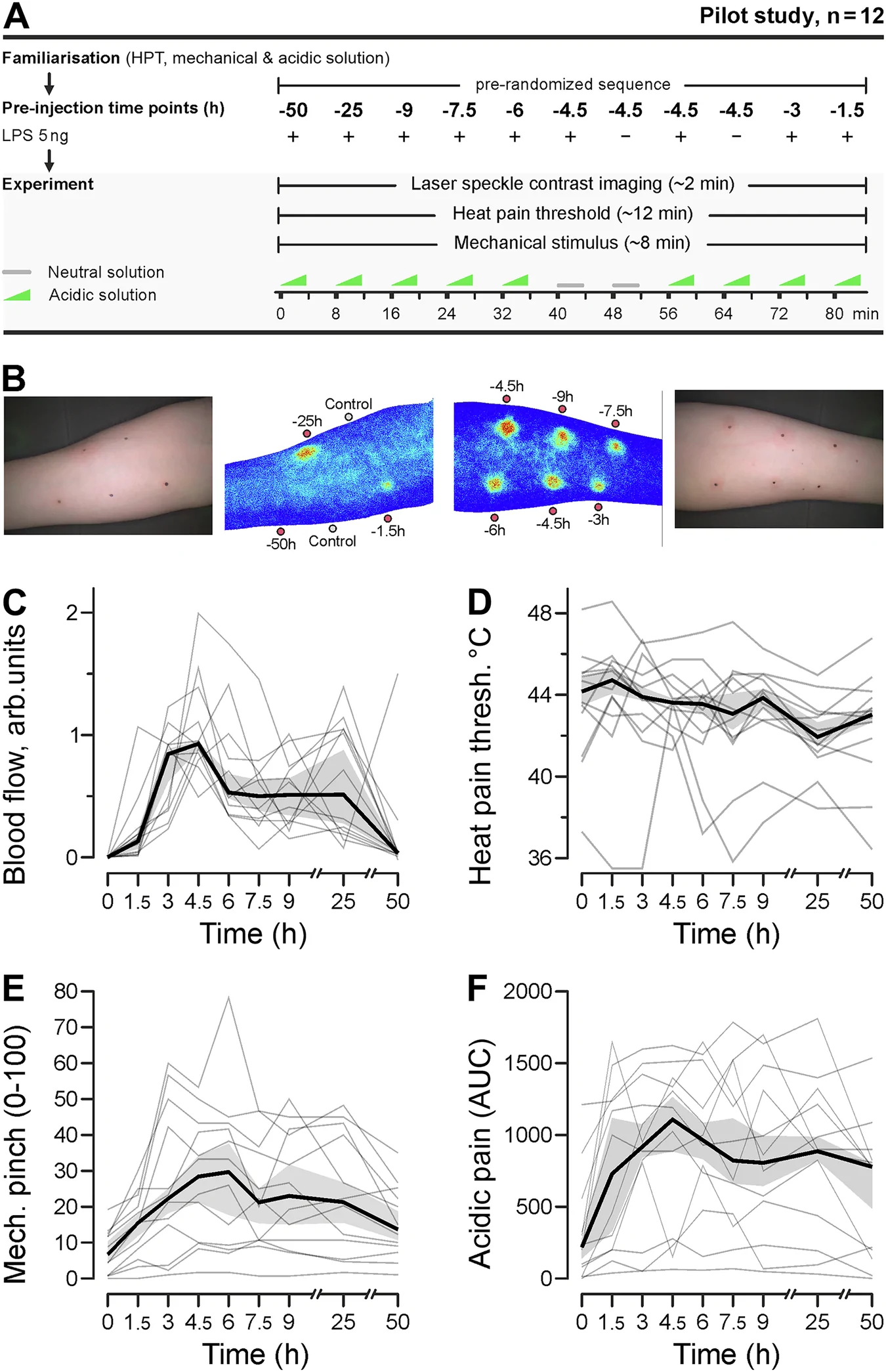
Time course of blood flow and pain sensitivity in the LPS inflammation model. **A** Protocol of the pilot study: all 12 participants completed the protocol. **B** Image and laser speckle-based blood flow imaging of a representative subject. LPS was injected 1.5–50 h earlier as indicated. Blood flow was acquired before subsequent testing. Due to injections with and without acid thereafter, the blood flow without LPS treatment and the blood flow 4.5 h after LPS were measured twice in each of the 12 subjects. **C** Hyperaemia was detectable at 1.5 h, peaked at 4.5 h after LPS injection, and recovered within 50 h to baseline. **D** Heat pain threshold was not systematically altered by LPS-induced inflammation. **E** Numerical rating of the pain intensity induced by a 2560 mN mechanical pinch stimulus application reached a peak at 6 h. **F** AUC pain induced by increasingly acidic injections. In (**C**–**F**), individual traces of all 12 participants are shown by grey lines, the median in black, and 68% CI is shaded in grey. Data for the two spots without LPS (i.e., time point = 0) and the two spots 4.5 h after LPS application were averaged. Source data are provided as a Source Data file.

The time course of blood flow and pain sensitivity, triggered by intradermal LPS application, was assessed in 6 female and 6 male volunteers with a median age of 23 years and a range of 20–29 years (Fig. 1A). Both blood flow, measured by laser speckle imaging, and sensitivity to increasingly acidic intradermal injection, rated on a 0–100 numerical scale, peaked at 4.5 h after LPS injection (Fig. 1B, C, F). Hypersensitivity to mechanical pinch was close to the maximum after 4.5 h, but had its peak at 6 h after LPS injection (Fig. 1E). Thus, the 4.5 h time point of the pilot study appeared most suitable for the main study. The heat pain threshold was surprisingly not altered to a relevant extent by inflammation (Fig. 1D) and was therefore omitted for the main study. The increased sensitivity to increasingly acidic injections due to LPS-induced inflammation receded more slowly than other modalities (Fig. 1F, Supplementary Fig. 1). After 50 h, the hyperaemia had subsided. However, compared to the peak sensitivity, after 50 h, about half the mechanical hypersensitivity and the majority of the hypersensitivity to acid remained.

### LPS-induced hyperaemia depends on RAGE, but not TLR4

**Fig. 2:**
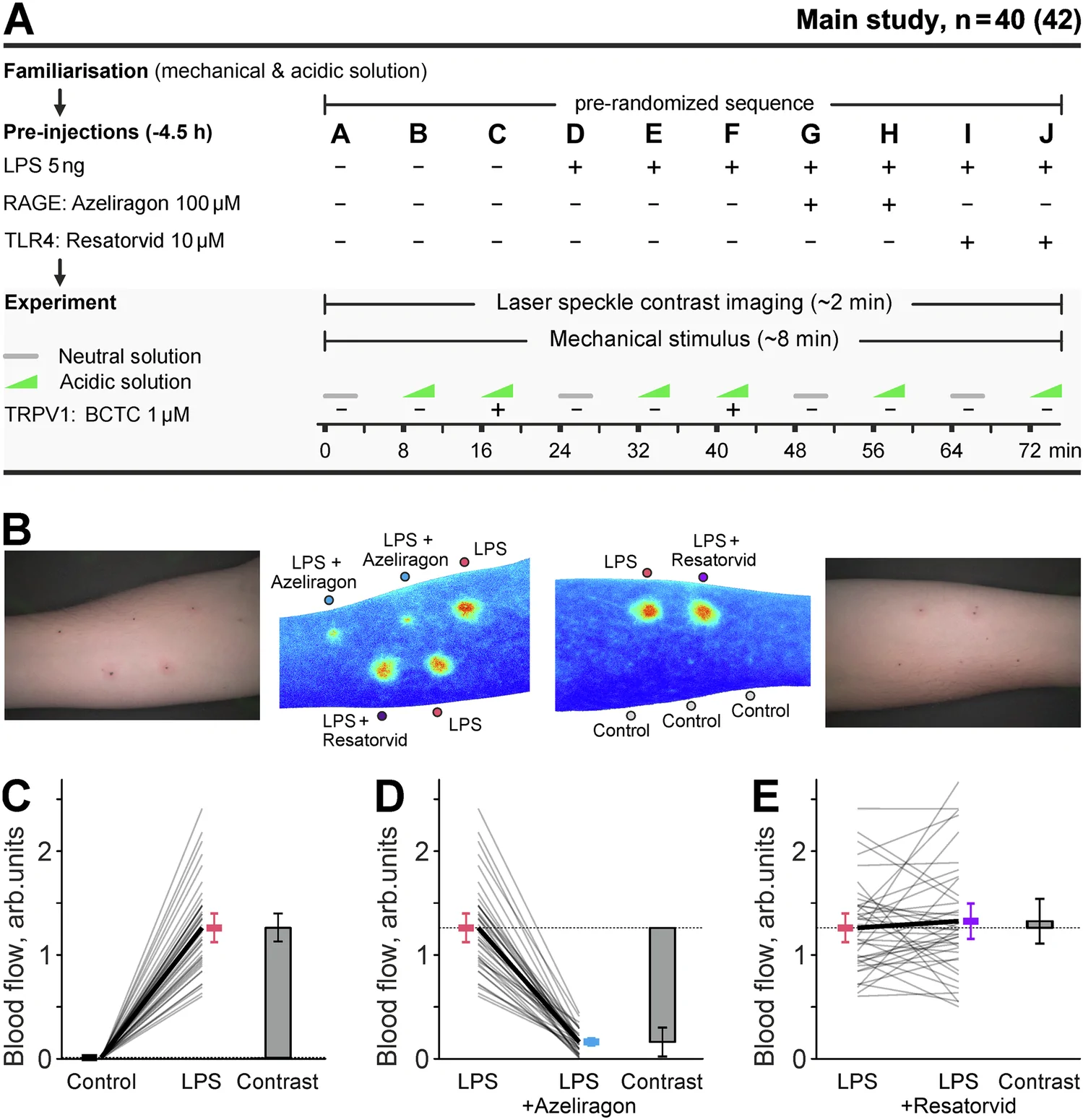
Hyperaemia in the LPS inflammation model largely depends on RAGE, but not TLR4. **A** Protocol of the main study with 42 participants, 40 completed the protocol. **B** Representative subject for laser speckle-based blood flow imaging at 4.5 h after control injections without LPS and LPS injections, both measured in three spots. RAGE antagonist azeliragon and TLR4 antagonist resatorvid were coapplied with LPS in two spots each. **C** Blood flow was increased in all LPS-injected spots in all 40 subjects. Mean estimates are provided with 95% CIs and the contrast between the two groups. **D** In comparison to LPS-induced hyperaemia, coapplication of azeliragon 100 µM reduced blood flow by 87% (estimated percent change, CI 84–89%, *n* = 40), which was the primary endpoint of the main study. **E** In contrast, the mean and confidence interval after coapplication of resatorvid 10 µM indicates no relevant dependence of blood flow on TLR4. Spaghetti plots indicate the pairwise comparison within each of the 40 subjects. Source data are provided as a Source Data file.

The main study had 42 participants. In two participants, the protocol was not completed (data not further considered), when it became apparent that the order of the prespecified sequence had not been followed correctly. 20 female and 20 male subjects, with a median age of 26 years and a range of 20–40 years, completed the protocol (Fig. 2A). In two participants, the protocol was not completed (data not further considered), when it became apparent that the order of the prespecified sequence had not been followed correctly. The blood flow 4.5 h after control injection was 0.008 arbitrary units (CI 0.005–0.011), which is similar to non-injected skin. LPS increased the blood flow after 4.5 h to 1.26 (CI 1.12–1.40, Fig. 2B, C), which is an increase similar to the pilot study. The primary endpoint of the main study was the comparison of blood flow in skin spots injected with LPS alone vs. RAGE antagonist azeliragon co-injected with LPS. Compared to LPS alone, the addition of azeliragon reduced skin hyperaemia by 87% to 0.16 (CI 0.13–0.20, *p* < 0.0001, Fig. 2D). In contrast, the TLR4 antagonist resatorvid did not alter skin hyperaemia (105% of LPS alone, mean estimate 1.32, CI 1.15–1.50, Fig. 2E). Exploratory addition of sex to the mixed model did not indicate sex-dependent differences in blood flow (‘sex’*’injection type’ *p* = 0.44).

### LPS-induced inflammation sensitises acid-induced pain

**Fig. 3:**
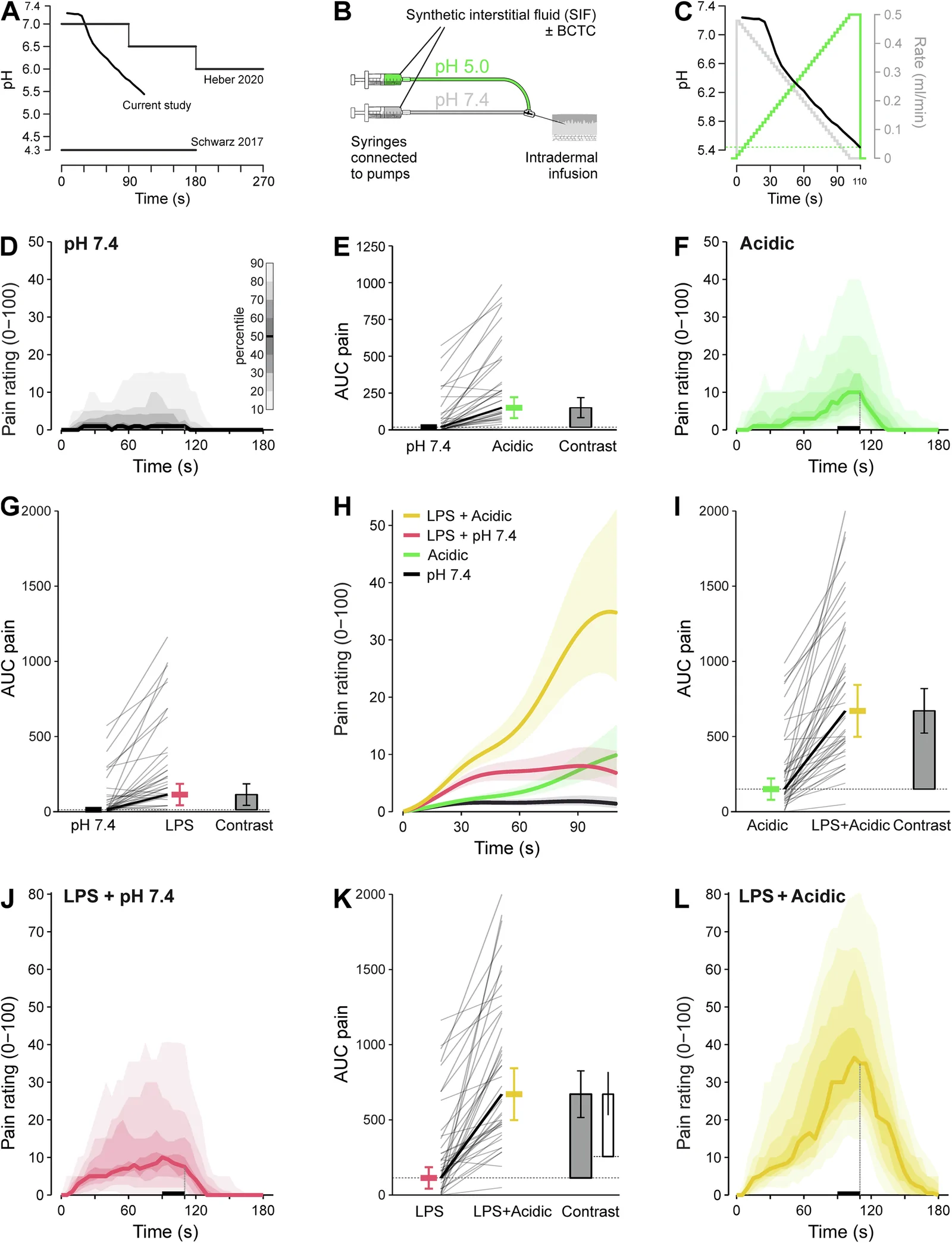
LPS-induced inflammation increases acid-induced pain. **A** Acid stimulation in prior studies, from a fixed acidic pH 4.3, to three acidic levels, to the pH ramp of the current study. **B** Improved acid-induced pain model with increasingly acidic pH, mixed from a pH 7.4 and 5.0 fluid. **C** Adaptation of the rates of two pumps resulted in a largely linear pH ramp (mean of three measurements). The time is aligned with the panel below. **D**, **F**, **J**, **L** Time courses of pain ratings, the distribution is visualised with the median as a solid line and decreasing colour shades for percentiles more distant from the median in 10% percentile steps, as indicated by the scale bar in (**D**). The injection lasted 110 s, and the end is visualised by a vertical dashed line. AUC pain was calculated for the period 90–110 s, marked by a bar on the x-axis. **E**, **G**, **I**, **K**. Mean estimates with 95% CIs for the AUC pain, spaghetti plot and contrast between the two groups for all 40 subjects, for which the time course is given in the two adjacent diagrams. (**K**) also visualises the contrast (open bar) for the prespecified hypothesis that pain induced by acid, in addition to mechanically induced pain, is greater after LPS injection than after control injection. The distance between the dotted lines equals the contrast in (**E**). **H** Overlay of a generalised additive model fitted to the data with estimated geometric mean and pointwise determined 95% confidence interval bands. Source data are provided as a Source Data file.

The acid-induced pain model was refined from the previous models, which had used a constant pH^25^ or three acidic levels^23^, to a pH ramp with a largely linear decrease from a pH of 7.24–5.44 in the current model (Fig. 3A–C). Pairwise comparison shows that in naïve skin increasingly acidic injections were more painful than pH 7.4 injections (Fig. 3D–F), pH 7.4 injections were more painful in inflamed skin compared to naïve skin (Fig. 3D, G, J), and increasingly acidic injections were more painful in inflamed skin than in naïve skin (Fig. 3F, I, L), and also compared to pH 7.4 injections in inflamed skin (Fig. 3J–L). Addressing the exploratory hypothesis, the difference in pain between increasingly acidic and pH 7.4 injections was indeed greater in inflamed vs. naïve skin (interaction’acid’*’LPS’ *p* < 0.001).

### RAGE is involved in LPS-induced sensitisation to tissue acidosis

**Fig. 4:**
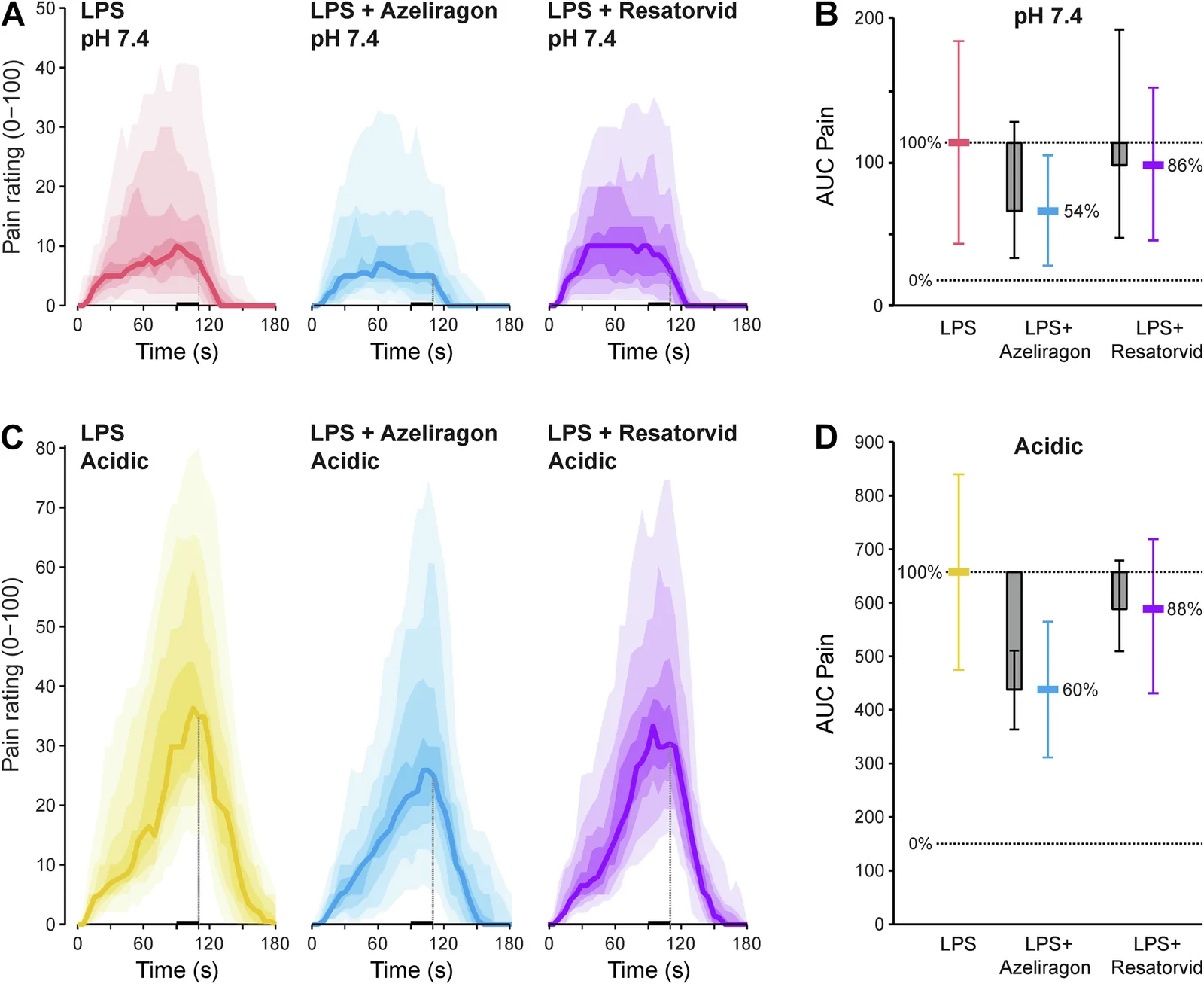
Acid hypersensitivity depends on RAGE, but not TLR4. **A** Time courses of pain ratings for injections with constant pH 7.4, measured 4.5 h after LPS injection. The distribution is visualised with the median as a solid line and decreasing colour shades for percentiles more distant from the median (in 10% percentile steps). The dotted vertical line indicates the end of the 110 s intradermal injection. **B** Mean estimates of pain in the period 90–110 s (AUC pain) with 95% CIs. Bars show the contrasts to only LPS for azeliragon and resatorvid with 95% CIs. A percent scale indicates the results without (0%) and with (100%) LPS pretreatment. **C** Time courses of pain ratings for injections with increasingly acidic pH 7.2–5.4, visualised as in (**A**). Results without antagonists in (**A**) and (**C**) are identical to Fig. 3J, L. **D** Mean estimates of pain in the period 90–110 s (AUC pain) with 95% CIs and contrasts for azeliragon and resatorvid. Percentages are provided on a scale without (0%) and with (100%) LPS pretreatment. Source data are provided as a Source Data file.

In experiments with pH 7.4 injections, co-injection of azeliragon with LPS resulted in a nominal reduction of the AUC Pain by 47 (CI -20–114, *p* = 0.11), corresponding to 54% remaining pain (control 0%, LPS 100%, Fig. 4A, B). To test the secondary hypothesis 1, acid-induced pain was compared between skin spots injected with LPS vs. LPS and azeliragon. Compared to LPS alone, the addition of azeliragon reduced the AUC pain by 207 (CI 100–315, *p* < 0.001), which corresponds to 60% remaining pain (control 0%, LPS 100%, Fig. 4C, D). There was no evidence for an effect of resatorvid co-injected with LPS, neither in pH 7.4 injections, nor in acidic ones (*p* = 0.67 and 0.13). Exploratory addition of sex to the mixed model did not indicate sex-dependent differences by the intradermal injections (‘sex’*’injection type’ *p* = 0.12).

### RAGE mediates the mechanical sensitisation by LPS

**Fig. 5:**
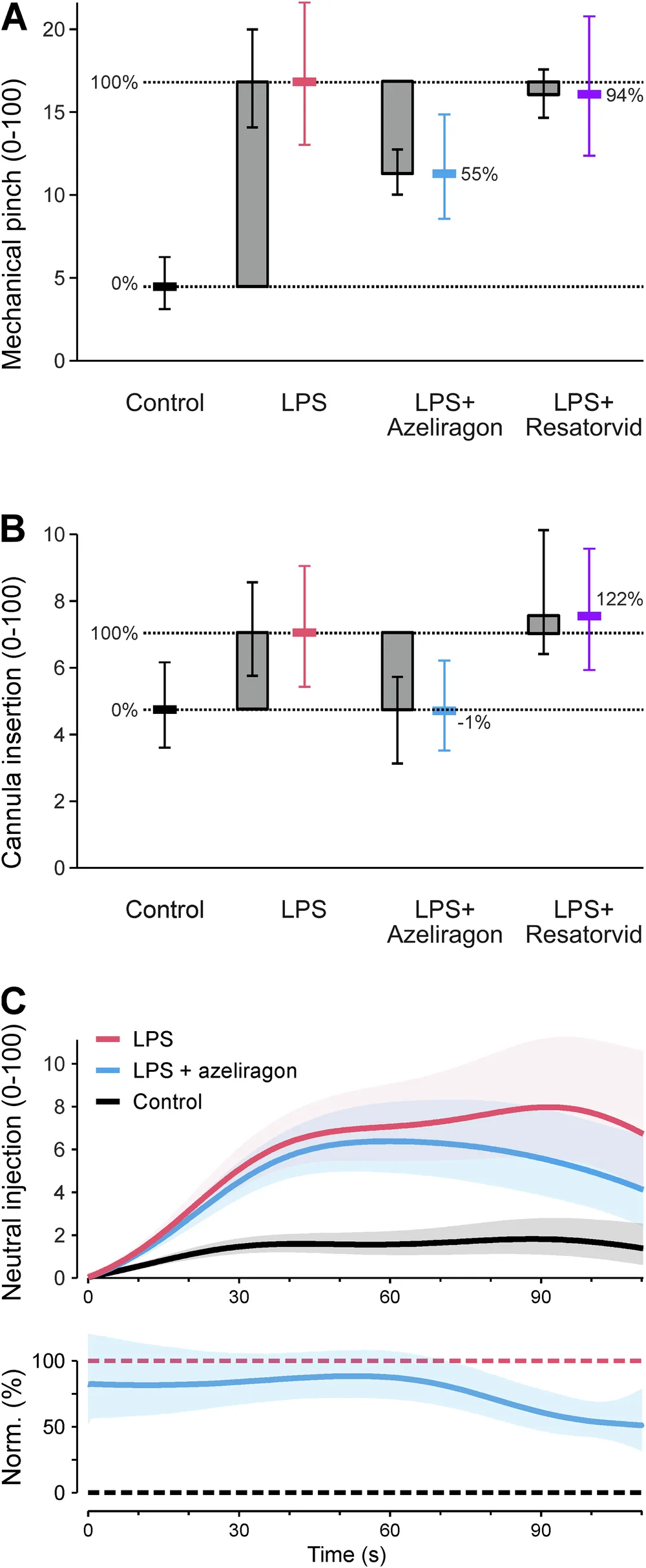
Mechanical hypersensitivity depends on RAGE, but not on TLR4. **A** Mean estimates and 95% CIs of mechanical pinch pain ratings, with an increase in mechanical pain 4.5 h after LPS-induced inflammation. Normalised to this sensitisation, coapplication of azeliragon with LPS reduced the mechanical pain to 55%. Resatorvid coapplication with LPS did not alter mechanical pain. Percentages of mean estimates are provided on a scale without (0%) and with (100%) LPS pretreatment. **B** Mean estimates and 95% CIs of cannula insertion pain ratings, again with an increase in mechanical pain 4.5 h after LPS-induced inflammation. Normalised to this sensitisation, coapplication of azeliragon with LPS reduced the mechanical pain to −1%. Resatorvid coapplication with LPS did not alter mechanical pain. **C** Generalised additive model for the experimental groups with pH 7.4 intradermal injection. Estimated geometric mean with pointwise determined 95% confidence intervals. Toward the end of the injection, there was a difference due to azeliragon pretreatment, which is more apparent when the ratings of pH 7.4 injections are normalised to control (0%) and LPS (100%, bottom panel, pointwise posterior sampling, reported with median and 95% confidence intervals). Resatorvid had no relevant effect and was omitted for readability. Source data are provided as a Source Data file.

With the chosen self-closing forceps, a mechanical pinch stimulus was established, which is mildly painful in naïve skin, but clearly sensitised in inflamed skin. The pain rating of 4.5 (CI 3.1–6.2) induced by mechanical pinch in naïve skin was increased to 17 (CI 13–22) in inflamed skin (Fig. 5A). For the secondary hypothesis 2, the mechanically induced pain was compared between skin spots injected with LPS alone and spots co-injected with azeliragon and LPS. The increased pain due to mechanical pinch in inflamed skin was reduced to 55% (11.3, CI 8.6–14.9) by the addition of azeliragon. In contrast, addition of resatorvid did not alter sensitivity to mechanical pinching (16.1, CI 12.4–20.8). For the mechanical pinch stimulus, the variance of repeated stimuli was assessed (Supplementary Fig. 2).

Cannula insertion pain is also mechanically induced, and results are in line with mechanical pinch pain. LPS elevated the cannula insertion pain from 4.7 (CI 3.6–6.2) in naïve skin to 7 (CI 5.4–9.0) in inflamed skin (Fig. 5B). The increased cannula insertion pain in inflamed skin was abolished by the addition of azeliragon, but was unaffected by the addition of resatorvid (4.7, CI 3.6–6.2 and 7.6, CI 5.9–9.6, respectively).

The pH 7.4 injections generate a slight local distension, which caused substantially more pain after induction of inflammation by LPS (Fig. 5C). A generalised additive model was used to quantify the effect of azeliragon, which reduced the LPS-elevated pain toward the end of an injection. Resatorvid had no relevant effect.

Exploratory addition of sex to the mixed model did not indicate sex-dependent differences in pain due to mechanical pinch (‘sex’*’injection type’ *p* = 0.17) or due to cannula insertion into the skin (‘sex’*’injection type’ *p* = 0.29).

### TRPV1 mediates acid-induced pain in LPS-induced inflammation

As an exploratory analysis, the contribution of TRPV1 to acid-induced pain in inflammation was investigated, considering that this is the principal detector in naïve human skin^23^.

**Fig. 6:**
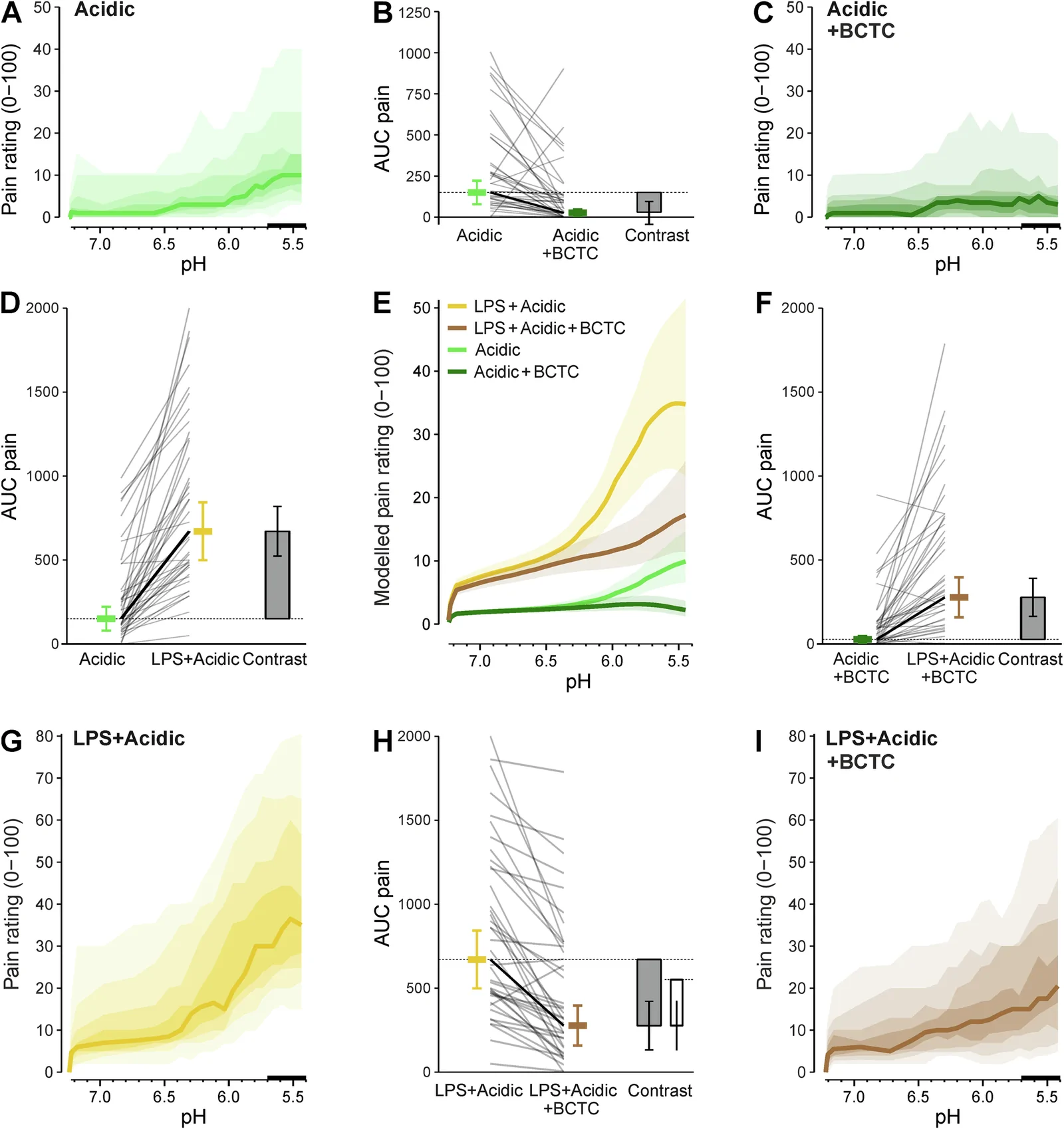
TRPV1-dependence of acid-sensing in inflammation. **A**, **C**, **G**, **I** PH-Dependence of the reported pain, the distribution is visualised with the median as a solid line and decreasing colour shades for percentiles more distant from the median (in 10% percentile steps, as indicated by the scale bar). This is based on the 110 s period of increasingly acidic injection, the time-dependent ratings are provided in Supplementary Fig. 3. Data in (**A**) and (**G**) are identical to Fig. 3. **B**, **D**, **F**, **H** Mean estimate with 95% CIs for the AUC pain, calculated for the range of pH 5.7–5.4 (period 90–110 s, marked by a bar on the x-axis), spaghetti plot for all 40 subjects and contrast between the two groups with adjacent diagrams. (**H**) also visualises the contrast (open bar) for the prespecified hypothesis that the inhibition of acid-induced pain by adding BCTC to the injection solution is greater after LPS injection than after control injection. The distance between the dotted lines equals the contrast in (**B**). **E** Overlay of a generalised additive model fitted to the data with estimated geometric mean and a 95% confidence interval. Source data are provided as a Source Data file.

The present study confirms the prior results, with TRPV1 antagonist BCTC largely reducing the acid-induced pain (−123, CI −189 to −57, Fig. 6A–C). Also in LPS-induced inflammation, the majority of the reported pain was dependent on TRPV1 (−393.2, CI −538.0 to −248.4, Fig. 6G-I). The fraction of acid-induced pain not inhibited by BCTC was 11% (bootstrapped CI −1 to 31%) in naïve skin and 29% (bootstrapped CI 11–51%) in inflamed skin, which was not significantly different (*p* = 0.16, Supplementary Fig. 3A).

Based on the generalised additive model used in the preceding analyses, posterior sampling was employed to estimate the pH required to produce a given difference in pain rating between two injection types. The pH required to increase the pain rating by, e.g., 1 unit on a 0–100 scale was 6.5 in non-inflamed control spots, but this shifted to pH 7.2 in inflamed spots (Supplementary Fig. 3B, C). Although TRPV1 explains the majority of the acid-induced pain, it is not the most sensitive pH sensor in the presented model. In naïve skin, lowering the pH below 6.5 raised the pain by 1 unit, whereas a reduction of pain by 1 unit due to TRPV1 blockade only occurred below pH 6.1 (Supplementary Fig. 3C, D). Consequently, the acid-induced pain observed between pH 6.5 and 6.1 cannot be attributed to TRPV1 and therefore remains unexplained.

Similarly, in inflamed skin, the pH threshold defined as an increase of 1 pain unit was about 7.2 or higher (with the range above not well addressed by the chosen pH). TRPV1, however, only explained a difference of 1 pain unit below pH 7.0 (Supplementary Fig. 3C, D). This means that what has been observed in non-inflamed skin also applies to inflammation: there is a pH range in which protons already activate nociceptors, but independent of TRPV1.

Direct overlay illustrates the difference between the RAGE-dependent general reduction of inflammation and the TRPV1 component identified by BCTC (Supplementary Fig. 4). Blocking RAGE reversed about half of the LPS-induced hyperalgesia across the entire examined pH range. In contrast, BCTC attenuated pain below the respective threshold of TRPV1, but reached a greater degree of pain inhibition than RAGE blockade. The full-time course of pain ratings for the injection-based experiments is provided for all participants (Supplementary Fig. 5).

Finally, associations of the primary and secondary outcome parameters were tested. There was no correlation between LPS-induced change in blood flow and acid sensitivity (partial Pearson *r* = −0.02, 95% CI [−0.21, 0.22]). There was also no correlation between LPS-induced change in blood flow and mechanical sensitivity (partial Pearson *r* −= −0.20, 95% CI [−0.39, 0.05]). To avoid false-positive correlation due to mediating parameters, data were adjusted (residualised) for acid-induced pain without LPS-induced inflammation or for mechanical pinch pain without LPS-induced inflammation, respectively. Individual subjective ratings of different painful stimuli should be expected to be correlated. To avoid this generating a false-positive correlation, data were adjusted for both pain ratings provided at the skin sites without LPS-induced inflammation (mechanical pinch and acid-induced pain). Importantly, the adjusted LPS-induced mechanical and acid sensitivity were positively correlated (partial Pearson *r* = 0.40, 95% CI [0.17, 0.61]).

All participants were contacted 72 h after completion of the study and reported no adverse effects. They also reported not taking anti-inflammatory drugs.

## Discussion

The time course of blood flow and pain sensitisation was quantified in a human LPS skin inflammation pain model. In this model, LPS-induced hyperaemia and pain hypersensitivity depend on RAGE, but not TLR4.

The most common bacteria causing skin and soft tissue infections are by far gram-positive *Staphylococcus aureus* and *Streptococcus pyogenes*, and there are experimental models for gram-positive infection, e.g., by intradermal injection of UV-killed *Streptococcus pneumoniae*^26^. Nevertheless, gram-negative bacteria, like Enterobactarales and *Pseudomonas aeruginosa*, are prevalent in up to 14%^27,28^, and mixed infections in up to 24% of culture-positive cases^28^. LPS makes up several percent of the total dry mass of gram-negative bacteria^29^. Being highly proinflammatory, it is established in cellular models^30^, animal models^31^ and also as an endotoxemia model in healthy subjects^32^. Comparison with other established inflammatory pain models, e.g., intraplantar injection of carrageenan in rodents, shows important differences. There are signs of inflammation, such as increased paw thickness and temperature, that peak at 2.5–4 h post-injection in rats^33^, just like hyperaemia does in the human LPS model. However, thermal and mechanical hypersensitivity mirror the time course of edema in the carrageenan model. This is clearly different in the presented human LPS model, where both mechanical and acid hypersensitivity recover more slowly than hyperaemia.

TLR4 is the established main target of LPS both in cellular and animal models^34,35^. TLR4 contributed to the activation of sensory neurons and to sensitisation of capsaicin responses^11,36^. In addition to TLR4, LPS binds directly to RAGE^13^, leaving the share of both TLR4 and RAGE and the relationship between them open^37^, and more so in human disease. Although direct effects have been reported for LPS 1 µg/ml and above^38^, there is little evidence for direct sensitisation of sensory neurons by LPS in a pathophysiologically relevant concentration. Consequently, a delayed response involving other cells, such as immune cells, appears to be essential.

LPS binding to RAGE might be facilitated by the nuclear factor HMGB1^39^, which can also be actively secreted or passively released by activated immune cells, like monocytes/macrophages^40,41^, or dendritic cells^42^. Serum HMGB1 levels were elevated in endotoxin-challenged mice and sepsis patients, and administration of antibodies against HMGB1 attenuated endotoxin lethality in mice^41^. These different pathways downstream of RAGE might explain the difference in the time course of hyperaemia and hyperalgesia.

Unexpectedly, there is no contribution of TLR4 in the LPS skin inflammation model, not to hyperaemia, not to mechanical pain hypersensitivity and not to acid-induced pain hypersensitivity. However, there is a substantial RAGE-dependent fraction, which varies across the readouts. Hyperaemia is almost completely RAGE-dependent, but less than half of acid-induced pain. The mechanism of LPS-induced sensitisation leading to the remaining fraction of mechanical and acid-induced pain is unclear. For example, the role of caspase-4/5 and a downstream pathway leading to NLPR3 inflammasome activation and IL-1beta/IL-18 secretion might be considered^43^.

For pharmacological inhibition of TLR4 and RAGE, substances that reached at least clinical phase II trials were considered, as this implies comprehensive preclinical toxicology studies and a phase I safety study. Paridiprubart (NI-0101), an anti-TLR4 monoclonal antibody, has reached clinical phase II trials. Eritoran (E5564), a synthetic lipid A analogue that antagonises the TLR4/MD2 complex, failed to show efficacy in phase III, just like resatorvid. Resatorvid was chosen because it is well established in preclinical research and because it is a small molecule. At present, azeliragon (TTP-488/PF-04494700) is the only RAGE antagonist having reached phase III clinical trials.

When considering the translation to other body sites, exposure to LPS ranges from almost none in the lungs to a massive commensal LPS load in the gut. Complementary to this, there is a strong inflammatory response to LPS in the lung, but a state of controlled tolerance in the gut. The skin represents an intermediate environment, with its commensal flora rich in Gram-negative bacteria. However, compared to mucosal surfaces, the role of skin-specific cell types needs to be considered. Keratinocytes can respond to LPS, releasing neutrophil attractant IL-8, monocyte and macrophage attractant MCP-1 and CCL2, IL-6 and TGF-beta^44,45^. In epidermal Langerhans cells, the combination of TNF/IL-1β and LPS supra-additively drives development of a mature immunogenic state^46^. Also, human dermal fibroblasts contribute to the LPS response by a non-canonical pathway^47^. Therefore, body site-specific cell types and signalling pathways could allow a context-sensitive response and require validating findings from skin inflammation at other sites.

With respect to the relative contribution of TRPV1 to acid-induced pain, in our prior study investigating the contribution of different ion channels to acid-induced pain, there was no inhibition by ASIC antagonist amiloride and TRPA1 antagonist A-967079. In contrast, application of TRPV1 antagonist BCTC largely reduced pain (70%, CI 53-84%)^23^. The present study shows that also in inflammatory conditions, acid-induced pain largely depends on TRPV1. However, there is a TRPV1-independent component, which remains mechanistically unresolved. Other receptors, such as TRPA1 or ASICs, were not investigated in the present inflammation-focussed study as the number of injections per participant visit was already high. Including additional hypotheses would have increased the required sample size due to adjustment for multiple testing. A potential future study addressing the small TRPV1-independent fraction of acid-induced pain would require a large sample size. Nevertheless, inhibiting TRPV1 has the potential to reduce pain in pathophysiological conditions involving tissue acidosis.

These findings raise the question of how inflammation affects the pH sensitivity and the activation threshold of TRPV1. TRPV1 has a threshold for activation at about pH 6.0^48,49^, and considering synergistic activation, pH 6.4 clearly sensitises against capsaicin or temperature activation^49^. A key feature of TRPV1 is the extensive, rapid and reversible sensitisation^50–52^. Also, pH sensitivity was demonstrated to be increased by, e.g., ATP, in ischaemia, or after induction of inflammation in animals^53–55^. However, the extent of a threshold shift was not thoroughly quantified in vitro. Unfortunately, studies reporting pH levels at which there is an increased pH response were not designed to detect a threshold, had widely spaced pH levels and lacked the gradual stimulus increase, all contributing to an underestimation of the threshold. The pain rating differences in the generalised additive model allowed quantifying the pH threshold shift in the LPS-induced human skin inflammation model. For example, the pain rating increased by a value of 1 on the 0–100 scale at a pH of 6.5 compared to pH 7.4 in naïve skin, but already at a pH of 7.2 in inflamed skin. The shift in pH sensitivity occurs for the TRPV1-dependent as well as for the TRPV1-independent fraction of pain. The observed level of increased pH sensitivity exceeds prior cellular data and reaches a level of pathophysiological relevance. Frequently occurring and mild reductions of pH can be expected to reach pH 7.2 and therefore activate nociceptors and contribute to pain in humans.

It is equally important to understand how inflammation modulates mechanical sensitivity, the second key sensory modality in this model. The assessment of suprathreshold pain was the main goal of the present study. The standard approach of the quantitative sensory testing battery^56^ to assess suprathreshold mechanical pain uses pinpricks. However, the strongest 512 mN pinprick stimulator of the quantitative sensory testing protocol generated low pain ratings in naïve skin and also did not reflect the marked mechanical hypersensitivity due to LPS-induced inflammation well in pilot experiments. In particular, lateral shear stress, e.g., even a mild stroke with a fingernail across the site, generated a pronounced difference in sensation between inflamed and naïve skin. The search for an adequate stimulus resulted in a mechanical pinch, delivered by self-closing forceps. In the study, this mechanical pinch generated no or marginal pain without inflammation in the majority of subjects, but was clearly painful in inflammation. To improve precision, this was measured three times; a higher number of tests was avoided, considering wind-up paradigms. The sequential ratings do suggest neither a sensitisation nor a desensitisation across the triplicate measurement. Azeliragon reduced the pain induced by pH 7.4 injection, which can only be attributed to mechanical phenomena. The needle insertion pain was analysed as an exploratory outcome. Nevertheless, it provided convergent evidence for the mechanical pinch results, as the needle insertion pain was also sensitised by LPS, and reduced by azeliragon but not by resatorvid.

Inflammatory sensitisation to heat is well established from cellular assays to human models, even including UV-based inflammation^57^. A TRPV1 contribution to the heat pain threshold could also be expected, due to the observed shift in TRPV1 sensitivity for acid detection. However, the pilot study surprisingly showed a lack of heat sensitisation in LPS-induced inflammation. A possible explanation is a modality-specific sensitisation, which was described to be largely dependent on a threonine at position 704 for sensitisation to heat, but sensitisation to acid relies on a serine at position 800^58^. Position 704 is mainly phosphorylated by PKCβII^59^, which requires a calcium elevation^60^. In contrast, amino acids 501 and 800 are mainly phosphorylated by the calcium-independent PKCε^61^. Thus, a limited calcium signal could lead to a selective phosphorylation, causing sensitisation to acid without a sensitisation to heat. Regulation of expression of protein kinases and altered signalling, e.g., by cytokines in the inflammatory response, should also be considered. The area of increased blood flow detected by laser speckle imaging 4.5 h after LPS injection corresponds to a circle with a diameter of 18 mm. The thermode stimulates 7.4 × 24.2 mm, so also surrounding skin. In the event of substantial sensitisation, it is expected that this will dominate heat sensitivity.

Considering safety of the presented model, the doses of LPS are low compared to the established intravenous LPS challenges with a dose range 0.06–4 ng/kg, reaching total systemic doses of up to 300 ng. Intradermally, 5–15 ng LPS were applied per injection with total doses per subject of up to 45 ng, leading to small-diameter inflammatory lesions that completely subsided within 48 h. Importantly, in these studies, no systemic signs or symptoms were observed^7–10^, which also applies to our study. LPS is slowly redistributed from the injection spot. It takes several days until 50% are removed. When it has reached the circulation, it is removed within minutes by the liver^62^. Therefore, after a peripheral LPS injection, at no point was more than 1% of the injected dose found in the plasma^63^. Both reproducibility and safety benefits from the highly standardised LPS production. Despite only a few investigations, the local intradermal LPS challenge can be considered an established and safe model of inflammation.

Limitations: It cannot be excluded that the relative importance of RAGE and TLR4 is different at another site. We aimed to provide a complete time course of the LPS-induced inflammation. The observation interval up to 50 h after LPS injection was based on pilot experiments, and with the assumption of a limited difference between the time course of blood flow and pain sensitivity. In hindsight, the observation period does not cover the whole hypersensitivity period until the end of the recovery process. Although there were no signs of residual inflammation after 72 h in pilot experiments, the respective time has not been acquired in the pilot study. Azeliragon was used at a relatively high local concentration (100 µM) to ensure robust RAGE blockade. This concentration was chosen to largely inhibit RAGE for a considerable period. However, the possibility of effects mediated by other targets cannot be ruled out. For example, p21-activated kinase 1 is inhibited by azeliragon^64^ and has a role in pain models^65^. Target engagement in human subjects, e.g., by displacement of a radioligand in a microdosing study, would be a valuable next step. The cannula insertion pain and the pain ratings in the first seconds of injection are different between naïve and inflamed skin. This could have provided a cue and limited the blinding of the subject. Using only a single molecular component of bacteria might not cover all aspects of a skin infection, as the model lacks other bacterial components. Nevertheless, this is outweighed by the advantage of a biologically well-defined model, triggering a reproducible degree and time course of inflammation.

Taken together, we have established a human pain model based on intradermal LPS administration. RAGE, but not TLR4, is crucial for mediating the resulting inflammatory hyperaemia and pain hypersensitivity in the skin, highlighting RAGE as a promising therapeutic target for inflammatory pain.

## Methods

Ethical approval was granted by the Ethics Committee of the Medical University of Vienna for investigating the time course of inflammation in a pilot study (vote 1569/2023). After completion, a study regarding the involved signalling mechanism and the contribution of TRPV1 was designed and approved (vote 1692/2024). Following comprehensive instruction regarding the nature, significance, impact and risks of this study, the subjects provided written consent to participate in the study. In addition, the participants received a written information sheet in comprehensible language, explaining the nature and purpose of the study and its procedures. The participants were informed that they could withdraw their consent at any time without providing reasons. In female subjects, a pregnancy was excluded by a test prior to any study-related intervention. Completion of the study visit was financially compensated as approved by the ethical committee. Participants had insurance for participation and were contacted 72 h after completion of the study to ask whether any unanticipated adverse effects had appeared and also whether anti-inflammatory drugs had to be taken. To comply with data protection laws, no protected information was digitised. Each participant is represented by a sequential number in all electronic records. The study was conducted in concordance with the principles of the ‘Declaration of Helsinki’ (as amended at the 64th WMA General Assembly, Fortaleza, Brazil, 2013) and with the laws and regulations of Austria. Only appropriately trained personnel were involved in the study. Before inclusion of the first subject, the main study was registered on www.clinicaltrials.gov (NCT07092215) on 11th of June 2025. The pilot study was carried out between 20th of November and 21st of December 2023, the main study between 17th of June and 11th of July 2025 at the Medical University of Vienna, Vienna, Austria.

### Experimental model of skin inflammation

In contrast to systemic LPS administration, dosed in the range 0.06–4 ng/kg^66^, local intradermal LPS application has been established with a dose of 5–15 ng per injection, leading to small-diameter inflammatory lesions that completely subside within 48 h^7–10^. A dose of 5 ng LPS per injection was validated in our own pilot experiments in which also, e.g., a tenfold lower dose caused hyperaemia and hypersensitivity. The pilot study had 9 injections containing LPS, the main study had 7 injections, resulting in a total dose of 45 and 35 ng per subject. After aspiration to ensure that small blood vessels were not punctured, a volume of 50 µl was superficially injected into premarked skin spots using a 30G syringe (BD, Micro-Fine U-100, 0.3 ×8 mm). The injection depth of about 1 mm from the skin surface allows visual control of the needle tip.

### Quantification of blood flow

The blood flow was quantified for both forearms by using the laser speckle contrast imager moorFLPI-2 (Moor Instruments, Axminster, United Kingdom) and the moorFLPI-2 Measurement V3.0 software. The forearm was placed on a black neoprene mat and the built-in aiming laser was used to standardise the 25 cm distance between the camera and the skin surface. The subject was asked not to move the forearm for 10 s to acquire a colour image and five speckle images sampled from 2 s each (20 ms exposure time with a 100 frames temporal filter). The least blurred laser speckle image was extracted as an 8-bit monochrome image with a background threshold of 10 (moorFLPI Review Software V6.0, Moor Instruments, Axminster, United Kingdom). ImageJ v1.54p was used for further analysis. The image was binarised to generate regions of interest marking inflamed skin spots by three ‘erode’ operations, followed by eight ‘dilate’ operations. Surrounding reference regions were generated by ten further ‘dilate’ operations. Pixel areas and mean grey values were then used to calculate a reference-subtracted volume integral (Δblood flow) for every skin spot. Non-inflamed skin spots were barely distinguishable from the background in the speckle image. Thus, using the colour image, a circle with a 3.2 mm radius around the injection spot (premarked by a pen) accompanied by a surrounding reference circle with a 4.9 mm radius was used to calculate the Δblood flow of non-inflamed skin spots.

### Subjective pain rating paradigms

Participants were first familiarised with the respective pain stimuli at naïve skin spots. For all rated stimuli, perceived pain was reported on a 0–100 scale, whereby 0 represents no pain, and 100 represents the worst imaginable pain. The heat pain threshold was determined by instructing participants to press a button when the increasing thermode temperature was first perceived as painful.

### Heat pain threshold

The heat pain threshold was assessed according to the standardised protocol for quantitative sensory testing^56^. The heat pain threshold was determined with a T11 probe operated by a TCSII thermal stimulator running with the software version 14.40.17 (QST.Lab, Strasbourg, France). Skin spots were stimulated with one activated zone (7.4 × 24.2 mm). Starting from 30 °C, a predefined temperature ramp with +1 °C/s was applied. Each skin spot was consecutively tested three times.

### Mechanical pinch

A mechanical pinch stimulus was realised using crossover forceps (Universalkreuzpinzette, 92 94 91, Knipex, Wuppertal, Germany), customised with a 3D-printed inset to have a maximal opening diameter of 6 mm, which fits the total area of erythema arising from the inflammation model used. For stimulation, the levers were fully opened by squeezing, gently placed onto the test site, and closed by releasing. At the resulting opening diameter of about 3 mm, the forceps apply a standardised pinch force of 2.560 mN. Forceps were removed after ~1 s, and the intensity of the perceived pain was rated by the subject. Each skin spot was tested three times in random order.

### Experimental model of acid-induced pain

To apply a defined nonhazardous intradermal acidic stimulus, previously established injection-based pain models^23,25^ were further improved to expose human skin to a largely linear pH ramp. Before each injection, a pH 7.4 and a pH 5.0 solution were loaded in 5 ml syringes (Injekt Luer Lock Solo) and connected to extension lines (Original Perfusor Line, 50 cm, both from B.Braun, Melsungen, Germany). The extension lines were connected to a 30G cannula by a 3-way stopcock (Sterican, 0.30 × 12 mm and Discofix 3SC, both from B.Braun, Melsungen, Germany). The syringes were inserted into two pumps (World Precision Instruments, Sarasota, FL) pre-programmed by using PumpTerm and DOSBox 0.74. The pH 5.0 syringe was flushed until the fluid reached the cannula tip, followed by flushing the 7.4 syringe for 5 s. For pH measurements, the fluid of three runs was collected in fractions of 5 s each (i.e., 22 fractions for a 110 s protocol) and pH was measured at room temperature using a calibrated pH microelectrode (Biotrode, 913 pH Meter, Metrohm, Herisau, Switzerland). This procedure was repeated three times. Over a period of 110 s, the two programmable pumps stepwise adapt their injection rates to generate a linear decrease from pH 7.24 to pH 5.44. The added injection rate from both pumps is at a constant 30 ml/h throughout the protocol, which results in a total volume of 0.92 ml administered per injection. The cannula was intradermally inserted into the respective spot on the volar forearm of the subject and fixed with Leukoplast (BSN medical, Hamburg, Germany). Pain arising from cannula insertion was then rated as a single number from 0 to 100. When the insertion pain had fully subsided, the pump protocol and an analogue stopwatch were started. Pain was rated every 5 s until the subject reported no pain for a period of 30 s. The pain ratings were noted on paper by the experimenter. After removing the cannula, at least two minutes elapsed before testing the next skin site.

### Applied solutions and substances

The buffered interstitial fluid, adapted from Bretag^67^, was used as previously described^68^. It contained (in mmol/L) NaCl 113.8, KCl 3.5, Na_2_HPO_4_ 1.7, MgSO_2_ 0.7, sodium gluconate 9.6, glucose 5.0, sucrose 7.6, CaCl_2_ 1.5, and histidine 22.0, dissolved in ddH_2_0 (Milli-Q system, Millipore, Burlington, MA). The solutions were titrated to a pH of 7.4 or a pH of 5.0, respectively, followed by sterile filtration using a 0.2 µm filter (Steritop, Merck, Darmstadt, Germany). LPS was acquired as a highly purified molecule obtained from *Escherichia coli* type O113:H10 (List Biological Laboratories Inc., Campbell, CA, USA)^69^ with a reported endotoxin activity of 10.3 EU/ng. LPS was stored in a stock solution of 1 mg/ml in ddH_2_0. For the injection solution, LPS was dissolved in interstitial fluid with a pH of 7.4 to a concentration of 100 ng/ml. The RAGE antagonist azeliragon has an IC_50_ of 1 µM^70^ and was orally dosed with 5–50 mg per day in three phase III clinical trials (NCT02080364, NCT02916056 and NCT05815485). Here, azeliragon (TargetMol, Boston, MA, purity 96.7%) was used at a concentration of 100 µM (~100-fold IC_50_), which was diluted from a 10 mM stock solution in DMSO. The total azeliragon dose per subject in the present study was 5.3 µg, corresponding to ~0.1% of the daily oral dose used in the phase III clinical trial. TLR4 antagonist resatorvid (IC_50_: 110 nM)^71^ was investigated in two phase III clinical trials (NCT00143611, NCT00633477; 2.4 mg/kg/d s.c.). Here, resatorvid (MedChemExpress, Monmouth Junction, NJ, purity 98.3%) was used at a concentration of 10 µM (~100-fold IC_50_), which was diluted from a 1 mM stock solution in DMSO. The total dose of resatorvid was 0.4 µg per subject, corresponding to ~0.0002% of the estimated daily s.c. dose used in the clinical trials. All pre-injections at time point 1 in the main study contained 1% DMSO.

TRPV1 antagonist BCTC (Cayman Chemical Company, purity 100%) was added from a 1 mM stock solution in DMSO. BCTC has an IC_50_ of 0.7 nM for acid-induced activation of human TRPV1^72^, and was previously used in injection-based pain studies at a final concentration of 1 µM^23,73,74^. In order to compensate for the adsorption of the lipophilic BCTC to the plastic tubing in the acid-induced pain model, the needle outflow from a regular protocol was collected and analysed by high-performance liquid chromatography (HPLC). BCTC was quantified using RP-C18 columns (150 × 3.0 mm, 2.5 μM; Kinetex, Phenomenex, Torrance, CA), in a Dionex Ultimate 3000 UHPLC (ThermoFisher, Waltham, MA) using Chromeleon 7.3.2. The mobile phase was a gradient from solvent A (water with 0.1% trifluoroacetic acid) towards solvent B (10% water, 0.1% trifluoroacetic acid, 90% acetonitrile, VWR Chemicals, Fountenay-sous-Bois, France) with a flow rate of 0.3 mL/min. The gradient started at 95% A/5% B and was linearly raised to 80% B over a period of 25 min. Absorbance was monitored at 254 nm. Results indicated a substantial loss of approximately 1/3 due to adsorption to tubing, which was compensated for by adjusting the concentration of the prepared solution by a factor of 1.5. The HPLC-validated final concentration was 102% of the target concentration. All injections at time point 2 in the main study contained DMSO 0.15%. All antagonist concentrations were chosen considering local dilution and redistribution.

Solutions used for pre-injections were aliquoted in 2 ml glass screw top vials with pierceable caps (polytetrafluorethylen/silicon, Agilent, Santa Clara, CA). Solutions used for the acid-induced pain model injections were aliquoted in 12 ml glass screw top vials with pierceable caps (ND20, Butyl/PTFE, DWK Life Sciences, Wertheim, Germany). All solutions were prepared, sterile filtered and aliquoted in June 2023 and stored at −20°C until the experimentation day (pilot study in November 2023, main study in June 2025). Substance stability was validated by HPLC in 2026. Each injection in the acid-induced pain model required two separate syringes; therefore, vial pairs were prepared and labelled ‘7.4’ and ‘5.0’, respectively. For non-acidic control injections, both vials contained pH 7.4 solutions, although the ‘5.0’ label was retained to blind the experimenter.

### Pilot study: Temporal profiling of LPS-evoked hyperaemia and hypersensitivity in human skin

The objective was to describe the time courses of LPS-induced skin blood flow, heat pain threshold, mechanical sensitivity and acid sensitivity. This first study was designed to provide the basis for sample size calculations for the subsequent confirmatory study, and to identify which measures have a relevant difference to the baseline within the observed 50 h period. The pilot study was prespecified not to investigate a hypothesis.

### Hypothesis-testing study: RAGE versus LPS-induced cutaneous sensitisation

The objective of this study was to elucidate the mechanisms of LPS-induced hypersensitivity. This objective comprises three independent, prespecified research questions. The mediation of proinflammatory effects of LPS via TLR4 has long been established. The question remained whether parts of the proinflammatory effects of LPS are also mediated through an alternative RAGE-dependent signalling pathway.

Primary hypothesis: ‘The blood flow is different in LPS-injected skin spots compared to LPS-injected skin spots where azeliragon is co-injected’.

Secondary hypothesis 1: ‘Acid-induced pain is different in LPS-injected skin spots compared to LPS-injected skin spots where azeliragon is co-injected’.

Secondary hypothesis 2: ‘Mechanical pinch-induced pain is different in LPS-injected skin spots compared to LPS-injected skin spots where azeliragon is co-injected’.

As a reference, the contribution of TLR4 was measured using the antagonist resatorvid. In addition, there were two prespecified, yet exploratory research questions. The first question addressed the sensitisation in the LPS inflammation model with the hypothesis ‘The pain induced by acid in addition to mechanically induced pain (by injection into the skin) is greater after LPS injection than after control injection’. This assessed the pain induced by injection of increasingly acidic (pH = 7.2–5.4) or neutral (pH = 7.4) solutions, allowing differentiation between purely mechanical hypersensitivity and additional hypersensitivity to acid. The second question addressed the role of TRPV1 in acid-induced pain in the LPS inflammation model with the hypothesis: ‘The inhibition of acid-induced pain by adding BCTC to the injection solution is different after LPS injection than after control injection’. This assessed the proportion of acid-induced pain attributable to TRPV1 activation changes due to LPS-induced inflammation.

### Inclusion and exclusion criteria

For both studies, the inclusion criteria were an age between 18 and 70 years and full legal capacity. Exclusion criteria were participation in another study, ongoing or within the last four weeks, current medication intake except hormonal contraception, drug abuse, body temperature above 38°C, known allergic diseases, in particular asthmatic disorders and skin diseases, a sensory deficit or haematoma of unknown origin in the physical examination of the test site, or symptoms indicating respiratory tract infection. Female subjects currently breastfeeding or with a positive pregnancy test provided at the beginning of the study visit were excluded. To ensure an equal number of each sex in the study population, once one sex reached half of the target sample size, only the other sex was included.

### Sample size

Twelve individuals were included in the pilot study, which allows estimation of the variance^75^. For the hypothesis-testing study, a Williams balanced Latin square design was chosen to minimise psychological carryover effects between injections and to ensure complete counterbalancing of treatment sequences. Ten different injection types were compared; therefore, this design required ten unique sequence combinations and a multiple of ten for the number of participants^76^. For each participant, one of the prespecified sequences was randomly selected and removed from the pool. For the primary hypothesis, the mean blood flow of LPS groups (D–F) is compared with the mean blood flow of LPS + azeliragon injected spots (G,H). The sample size calculation using pilot study data yielded a sample size of 10 participants for a relevant effect size of 35%, α = 0.05 and 1 − β = 0.8. For the secondary hypothesis 1, the acid-induced pain rating AUC Pain of LPS and LPS + azeliragon is compared (E vs. H). The sample size calculation using pilot study data yielded a sample size of 40 participants for a relevant effect size of 35%, α = 0.025 and 1 − β = 0.8. For the secondary hypothesis 2, the mean mechanical pinch pain rating of LPS groups (D,E,F) is compared with the mean mechanical pinch pain rating of LPS + azeliragon groups (G,H). The sample size calculation using pilot study data yielded a sample size of 20 participants for a relevant effect size of 35%, α = 0.025 and 1 − β = 0.8. To cope with up to 10% of participants not completing the study, ethical approval was applied for and received (EK 1692/2024) for up to 45 subjects.

### Transformation of injection-induced pain ratings

In accordance with the statistical analysis plan, several plausible within-subject covariance structures were evaluated, namely a compound-symmetry structure (constant within-subject correlation), a first-order autoregressive structure, and low-order autoregressive moving-average structures approximating a Toeplitz covariance. For each covariance structure, models with either homogeneous residual variance or injection-type–specific residual variances (one variance parameter per injection type) were fitted, yielding a predefined matrix of candidate models.

Given the marked right-skewness and variance heterogeneity of the raw AUC Pain values, three transformation families were examined: log₁₀(AUC Pain + 1), square-root(AUC Pain), and cubic-root(AUC Pain). Within each transformation family, all covariance structures and variance specifications were combined with the same fixed-effects structure. Candidate models were compared using the Akaike information criterion. Importantly, statistical significance (*p*-values, confidence intervals) was not considered at this stage; model selection was based solely on goodness-of-fit criteria and diagnostic performance. Across transformations, models based on log₁₀(AUC Pain + 1) provided the most favourable balance of residual normality, reduced heteroscedasticity and interpretability on the original scale. Within the log-transformed set, the model combining a compound-symmetry correlation structure with injection-type–specific residual variances yielded the lowest Akaike information criterion and exhibited no systematic residual patterns. This model was therefore selected as the final working model. Estimated means for each injection type were obtained on the log₁₀ scale and subsequently back-transformed to the original AUC Pain scale using the delta-method–based regridding implemented in the emmeans package, yielding geometric means of AUC Pain with 95% Wald confidence intervals.

Model diagnostics were assessed for all models by inspecting the standard nonlinear mixed-effects diagnostic plots (residuals vs. fitted values, Q–Q plots, and leverage diagnostics). Residuals showed no systematic patterns, and variance modelling using group-specific residual variances adequately accounted for heteroscedasticity.

Modelled results of central tendencies are reported throughout the manuscript as ‘mean estimates’, despite recognising that this measure can also be labelled as ‘estimated marginal means’, or as ‘least-squares means’. Further, estimated means from mixed-effects models on log-transformed data should be interpreted geometrically after back-transformation. For readability, this is not termed ‘geometric mean estimates’ but still ‘mean estimates’.

### Analysis of the primary hypothesis

According to the study protocol, the ten injection types (A–J, Fig. 2A) administered to each subject were grouped a priori into four groups: injections A, B and C constituted the group ‘Control’; injections D, E and F the group ‘LPS’; injections G and H the group ‘LPS+RAGE antagonist’; and injections I and J the group ‘LPS+TLR4 antagonist’. For statistical modelling, blood flow values from injections of the same group were averaged on a per-subject basis, yielding one value per group for each subject.

The primary hypothesis concerned the comparison of blood flow between ‘LPS’ and ‘LPS + RAGE antagonist’. For the inferential analysis, a linear mixed-effects model was fitted using restricted maximum likelihood as implemented in the nlme package in R. The within-subject mean of blood flow served as the dependent variable. The model included a four-level fixed factor group (‘Control’, ‘LPS’, ‘LPS+RAGE antagonist’, ‘LPS+TLR4 antagonist’) and a random intercept for each subject to account for the clustering of observations within subjects. Blood flow values were analysed on the original (untransformed) scale, with heteroscedasticity accommodated directly by allowing each condition to have its own residual variance. This was implemented using a variance-by-group structure (varIdent in nlme), which estimates a separate residual variance for each level of the group factor rather than assuming a common variance across all groups.

Estimated means and 95% Wald confidence intervals were obtained using the emmeans package (version 1.11.1) in R. The three contrasts described above were prespecified: the primary contrast (‘LPS’ vs. ‘LPS+RAGE antagonist’) corresponded to the main study hypothesis. As positive controls, two additional predefined contrasts were evaluated: ‘Control’ vs. ‘LPS’, assessing the expected increase in blood flow induced by LPS as established in the pilot study, and ‘LPS’ vs. ‘LPS+TLR4 antagonist’, based on the conventional assumption that LPS elicits inflammation through TLR4 signalling. The additional contrasts were included as confirmatory reference comparisons; therefore, no adjustment for multiple testing was applied.

### Analysis of injection-induced pain ratings

For descriptive inspection of all data involving injection-induced pain, individual time courses of pain ratings (0–100) were plotted for each injection type (A–J). For every subject, the pain ratings induced by the intradermal injection were shown as a separate line. To summarise central tendency and variability, percentile ribbon plots were generated for each injection type, displaying nested 10–90%, 20–80%, 30–70% and 40–60% percentile bands together with the median curve. Similar plots were produced with a pH-scaled x-axis, allowing a visual assessment of pain as a function of the pH decline during injection. Collectively, these descriptive plots provided an overview of the magnitude, time course and variability of injection-induced pain across injection types before statistical modelling.

Analyses involving injection-induced pain used the area under the curve of the pain ratings as a summary outcome. For each subject and injection type, pain was recorded on a 0–100 numeric rating scale at 5 s intervals until six consecutive 0 ratings were obtained. To obtain a standardised quantitative measure of the experienced pain, the area under the curve was calculated over the predefined inclusive interval 90–110 s, corresponding to the last 20 s of the injection. Within each subject and injection type, all pain ratings within this window were numerically integrated using the trapezoidal rule based on pain (0–100) and time (seconds), resulting in one area under the curve value for each injection type per subject. This value served as the outcome variable, area under the curve of pain (‘AUC Pain’) with the unit pain-seconds.

For the inferential analysis of AUC Pain, mixed-effects models were fitted with the individual AUC Pain values as the dependent variable. Injection type (ten levels, A–J) and the crossover period were included as fixed effects, and a random intercept for each subject accounted for the clustering of the ten injections within subjects.

### Analysis of secondary hypothesis 1

The secondary hypothesis 1 concerned the comparison of acid-induced pain between ‘LPS’ and ‘LPS+RAGE antagonist’. For the contrast analysis, all pairwise comparisons between injection types were obtained directly from the final mixed-effects model. On the log₁₀(AUC Pain + 1) scale, contrasts correspond to simple differences between the estimated means, resulting in ratios after back-transformation (i.e., multiplicative effects on the original AUC scale). As multiplicative results are not always intuitive for interpreting injection-related pain, additive differences on the original AUC scale were reported. These additive contrasts and their confidence intervals were computed using the regridding procedure in the emmeans framework, which applies the multivariate delta method to propagate uncertainty through the back-transformation and provides valid additive and multiplicative contrast estimates consistent with the underlying mixed-effects model.

### Analysis of secondary hypothesis 2

The secondary hypothesis 2 concerned the comparison of mechanical pinch-induced pain between ‘LPS’ and ‘LPS+RAGE antagonist’. Pain ratings elicited by the three brief mechanical pinch stimuli (pinch = 1–3) were first explored graphically. For each subject and injection type, the pain ratings of the three stimuli were averaged. Visual inspection of spaghetti plots stratified by injection type, pinch level, and grouped injection categories revealed no systematic trend across the three stimuli.

To obtain group-level estimates, the averaged pain ratings were analysed using a linear mixed-effects model with grouped injection types as fixed effects (‘Control’, ‘LPS’, ‘LPS+RAGE antagonist’, ‘LPS+TLR4 antagonist’), a random intercept per subject, and heterogeneous residual variances across groups. Residuals on the raw scale deviated clearly from normality and were therefore transformed to the log10(Pain + 1) scale. This yielded residuals that were approximately normal and homoscedastic, without systematic patterns. Estimated means and contrasts were obtained on the log scale and subsequently back-transformed to the original pain scale.

### Model-based parametric simulation to estimate the relative effect of TRPV1 inhibition on acid-induced pain

To determine the relative position of BCTC-containing acidic injections relative to acidic injections and pH 7.4 injections, we applied a parametric, model-based simulation that generates model-implied datasets from the fitted mixed model. This approach incorporates all estimated components of the model, including fixed effects, random intercepts, condition-specific residual variances, and the within-subject compound-symmetry correlation structure to obtain a model-consistent sampling distribution of predicted pain responses for each injection type.

For this purpose, 100,000 complete datasets were simulated under the fitted model, holding all parameters at their estimated values while incorporating the model-estimated random and residual variability. In each simulation, the expected log-transformed AUC Pain for every observation was obtained from the fixed-effect component. The subject-specific random intercept was added to preserve the between-subject variability encoded in the model, and residual variation was introduced using condition-specific standard deviations. To reproduce within-subject dependence, the residuals were drawn jointly from a multivariate normal distribution parameterised to match the compound-symmetry structure estimated in the original analysis. In this way, residual noise for the repeated observations of each subject was not generated independently, but in a way that preserved the correlation pattern observed within individuals. Each simulated dataset thus represents a plausible repetition of the experiment based on the fitted mixed model. Within every simulated dataset, pain responses were summarised as geometric means for each injection type, consistent with the log-transformed modelling framework. To express where the injection type lies between two reference injection types, these simulated geometric means were mapped onto a linear 0–100% scale by assigning the lower reference (pH 7.4 injections in control or LPS) a value of 0% and the upper reference (acidic injections in control or LPS) a value of 100%. The BCTC-containing acidic injection was then positioned proportionally between these anchors (in control and LPS, respectively). Repeating this transformation across all 100,000 simulations yielded an empirical distribution of percentages relative to the references. From these simulation-derived distributions, 2.5% and 97.5% quantiles were extracted as simulation-based 95% confidence intervals.

### Modelling the time course of pain using generalised additive mixed models

To estimate injection-type–specific differences in pain intensity continuously over time and to relate these dynamic effects to the concurrently changing pH, a generalised additive mixed model (GAM) was fitted to the raw pain ratings over time. For modelling, pain ratings were analysed on the log-transformed scale log₁₀(Pain + 1) to stabilise variance and accommodate the right-skewed distribution of the raw values, while retaining interpretability on the original scale via back-transformation.

The GAM was specified to allow flexible, injection-type–specific time courses while accounting for between-subject heterogeneity and period effects. Time in seconds after injection onset was used as a continuous predictor, and a separate smooth function of time was estimated for each injection type via a factor*smooth interaction. This was implemented by estimating a separate smooth function of time for each injection type. In mgcv, this is achieved using so-called “by-smooths”: each smooth term contributes only for observations belonging to its respective group and is zero otherwise. Given that the by-variable was defined as time itself for the respective injection type and zero otherwise, the contribution of each smooth is forced to be zero at time 0, such that all injection-specific curves are naturally anchored at the predefined fixed baseline of pain = 0 at time = 0. Each smooth was given a moderate basis dimension (k = 7), which limits the maximum number of effective bends it can exhibit and thus allows the main shape of the trajectories to be captured without overfitting noise. The effective wiggliness is then determined by penalisation, so that the estimated curve uses only as much flexibility as the data support. The adequacy of k = 7 was verified using mgcv’s k-index diagnostic, which showed no evidence that a larger basis (and thus a higher maximum permissible wiggliness of the smooth) would be required.

To represent subject-specific deviations from the population mean curve, a random-effect term was included as a smooth of subject identifier with a numeric-by-time structure, which behaves like a random slope over time for each subject. Each subject was allowed to have their own linear trend (a deviation in slope), while no subject-specific intercept or higher-order wiggles are estimated because the by-variable constrains the smooth to be zero at time 0. This model incorporates individual differences in rate of change over time, but not full subject-specific smooth curves.

In addition, a factor*smooth interaction between time and crossover period was included as a nuisance term to adjust for potential systematic differences in pain dynamics across periods. This means that for each crossover period, the model allowed the time course to assume a slightly different shape, ensuring that any period-specific variation in pain dynamics was not inadvertently attributed to the injection types. The model was fitted using restricted maximum likelihood as implemented in the mgcv package in R.

For inference on the population-level time courses, injection-type–specific least-squares mean curves (population smooths) were derived on a dense time grid covering 0–110 s in 0.5 s steps. These curves were obtained from the fitted GAM using its linear predictor matrix (the “lpmatrix” in mgcv), that is, the design matrix that maps the model coefficients to fitted values at the chosen grid points. In this step, subject-specific random effects were set to zero so that the resulting curves represent averages over subjects, and predictions were averaged over all period levels at the design-matrix level to obtain period-adjusted trajectories. Pointwise standard errors and 95% confidence intervals were then computed using the full covariance matrix of the GAM coefficients, ensuring that the uncertainty in each curve reflects the joint uncertainty in all model parameters. Finally, these predicted log_10_(Pain + 1) values and their confidence limits were back-transformed to the original 0–100 scale, yielding injection-type–specific, period-adjusted LS-mean pain trajectories with 95% confidence bands over time.

To express pain as a direct function of the injected pH level, the population time courses were subsequently projected onto a pH-scaled x-axis. For this purpose, a smooth calibration function relating time to injected pH from the pooled pH measurements obtained during acidic injection protocols was constructed (monotone Hyman spline for the range 5–110 s). Each time point on the prediction grid was then translated into a pH value using this calibration, which allows LS-mean pain trajectories and confidence bands as a function of pH instead of time. This yielded injection-type–specific LS-mean curves of pain versus pH with corresponding 95% confidence intervals on the original pain scale.

Model adequacy was evaluated by inspecting standard GAM diagnostics. Plots of standardised residuals versus fitted values and quantile–quantile plots of deviance residuals on the log-transformed scale did not reveal major deviations from model assumptions or residual patterns suggesting lack of fit.

Finally, to quantify differences in pain dynamics between selected injection types and to assess contrasts across the pH range, we derived pairwise and interaction contrasts directly from the fitted GAM. For each contrast of interest, appropriate linear combinations of the GAM coefficients were constructed on a common pH grid using the linear predictor matrix. Differences in estimated means of pain between conditions, as well as their interaction contrasts, were then expressed on the original pain scale using a multivariate delta-method approximation to propagate the coefficient uncertainty through the back-transformation from log_10_(Pain + 1).

### Posterior sampling of pH thresholds for prespecified pain differences

To identify the pH level at which two injection types differ by a given amount of pain, a threshold-based analysis was carried out on posterior draws from the GAM. Specifically, this determined the pH value at which the model-implied difference in expected pain between two injection types first reached a threshold (e.g., 1 pain unit), together with corresponding uncertainty intervals.

Based on the fitted GAM for log_10_(Pain + 1), first, the joint sampling distribution of all model coefficients was approximated by a multivariate normal distribution with mean equal to the restricted maximum likelihood estimates and a covariance given by the unconditional coefficient covariance matrix returned by mgcv. From this distribution, 10,000 coefficient vectors were drawn, each representing one plausible realisation of the underlying population-level model. For each draw, and for each injection type, period-averaged population predictions of pain over a dense time (0.5 s) grid in the range 0–110 s were constructed. This yielded, for every posterior draw, a set of smooth, noise-free time courses of expected pain for all injection types.

For contrasts of interest (‘Acidic’ vs. ‘Control’, ‘Acid’ vs. ‘Acid + TRPV1 antagonist’, ‘LPS + Acidic’ vs. ‘LPS’, ‘LPS + Acid’ vs. ‘LPS + Acid + TRPV1 antagonist’) within each draw, the difference in expected pain between the two injection types was calculated as a function of time. For each posterior draw and each pain-difference threshold, the earliest time at which the modelled difference reached the threshold was identified by scanning through the predicted time points. If the threshold lay between two adjacent time points, the exact crossing time was obtained by linear interpolation between those neighbouring points. For each threshold and each contrast, the collection of ‘first-exceedance times’ across the 10,000 draws forms an empirical sampling distribution for the time at which the model-implied pain difference first attains the specified magnitude. From these distributions, the median as a central estimate and the 2.5th and 97.5th percentiles as 95% simulation-based confidence intervals were extracted. To avoid reporting thresholds for which the corresponding posterior differences were not consistently reached, we restricted the analysis to those pain-difference values that were exceeded by all posterior draws within the observed time window. For higher thresholds, some posterior difference curves never reached the required magnitude (yielding undefined first-exceedance times), which precludes the computation of valid quantiles and would imply extrapolation beyond the data-supported region.

To express these thresholds on the pH scale, the first-exceedance times were mapped to pH using the previously constructed calibration function. Applying this calibration to the threshold crossing times yielded, for each contrast and each pain threshold, a posterior sample of pH values at which the model-implied pain difference first reached the specified magnitude. Again, median values and 95% simulation-based confidence intervals were reported, providing pH-resolved estimates and uncertainty bands for the shift in pH value, generating a difference of 1–3 pain units between injection types. The difference in pH thresholds between inflamed and non-inflamed skin quantified the shift in the pH–pain relationship by inflammation.

### Analysis of pain induced by cannula insertion

Pain ratings elicited by cannula insertion were first averaged within the four groups (‘Control’, ‘LPS’, ‘LPS+RAGE antagonist’, ‘LPS+TLR4 antagonist’), yielding one value per group for each subject. These means were analysed with a linear mixed-effects model on the log₁₀(Pain + 1) scale, with group as a fixed factor, a random intercept for subjects, and a variance-by-condition structure to allow for heteroscedastic residuals. The model was fitted by restricted maximum likelihood using the nlme package in R.

Estimated means on the log₁₀ scale were obtained using emmeans, back-transformed to the original 0–100 scale, and used to form prespecified contrasts (‘Control’ vs. ‘LPS’, ‘LPS’ vs. ‘LPS+RAGE antagonist’, ‘LPS’ vs. ‘LPS+TLR4 antagonist’). The antagonist effects were normalised onto a scale with ‘Control’ as the 0% reference and ‘LPS’ as the 100% reference. For the groups containing the RAGE and the TLR4 antagonist, the fraction of the LPS-induced increase in insertion pain was calculated. This was done by comparing how much higher the back-transformed LS-mean was than in the ‘Control’ group, relative to the corresponding difference between the ‘LPS’ and ‘Control’ groups. The four estimated means are correlated, and this percentage transformation is nonlinear; therefore, confidence intervals for these percentages were obtained by parametric resampling from the multivariate normal distribution of the fixed effects, recomputing estimated means and percentages for each draw. The resulting empirical distributions were summarised by their medians and 95% simulation-based confidence intervals, which reflect the joint parameter uncertainty.

### Analysis of sex-dependent effects

For all prespecified analyses, a subanalysis was performed to investigate potential differences between female and male subjects by including ‘sex’ and its interaction with the respective factor of interest in the model.

### Reporting summary

Further information on research design is available in the Nature Portfolio Reporting Summary linked to this article.

## Supplementary information

Supplementary Information

Reporting Summary

Transparent Peer Review file

## Source data

Source Data

## Data Availability

All data collected from subjects during the study, including blood flow and pain ratings, are provided in the Source data file. Data has been de-identified, and subjects are represented by numbers. The approved Study Protocol and CONSORT form accompany this study. Source data are provided with this paper.
