# Supplementary material for: RAGE mediates LPS-induced inflammatory pain in human skin – a double-blind, randomised, placebo-controlled full crossover study
Source: Nat Commun. 2026 Sep 2;17:10461. doi: 10.1038/s41467-026-76771-2 (PMC13635236; doi:10.1038/s41467-026-76771-2)
Supplement: Supplementary file 1 [file 41467_2026_76771_MOESM1_ESM.pdf]

Supplementary Information

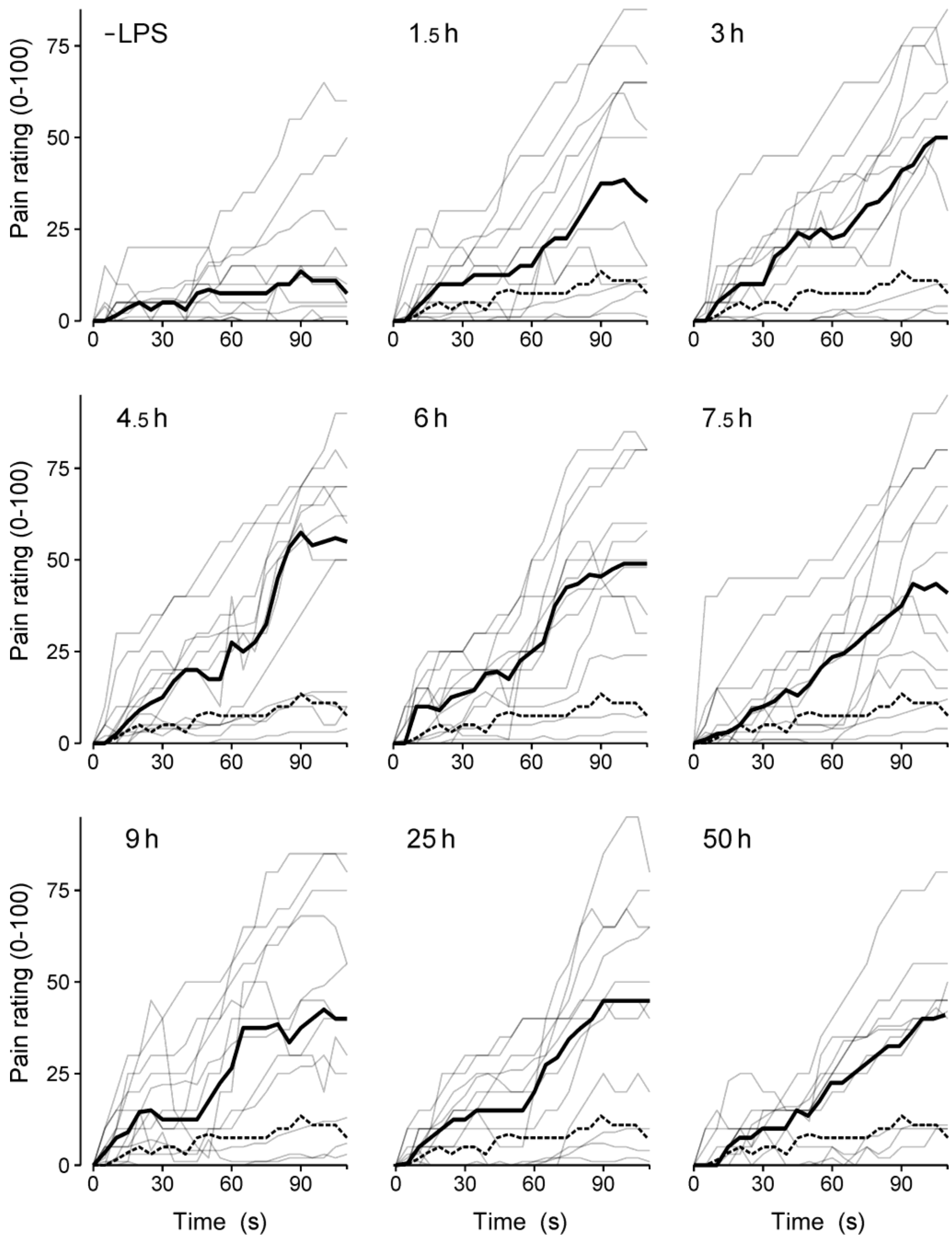

Supplementary Figure 1. Time course of LPS-induced acid sensitisation. For all 12 participants of the pilot study, the pain due to the increasingly acidic injection is visualized. Compared to the observations without LPS (-LPS), the period after intradermal injection of 5 ng LPS is provided. All panels visualize the median (black line) and the median obtained without LPS provide a reference (dotted line). Note that there is a gradual increase until 4.5 h after LPS, and a rather stable level in the period 4.5–9 h after LPS. Source data are provided as a Source Data file.

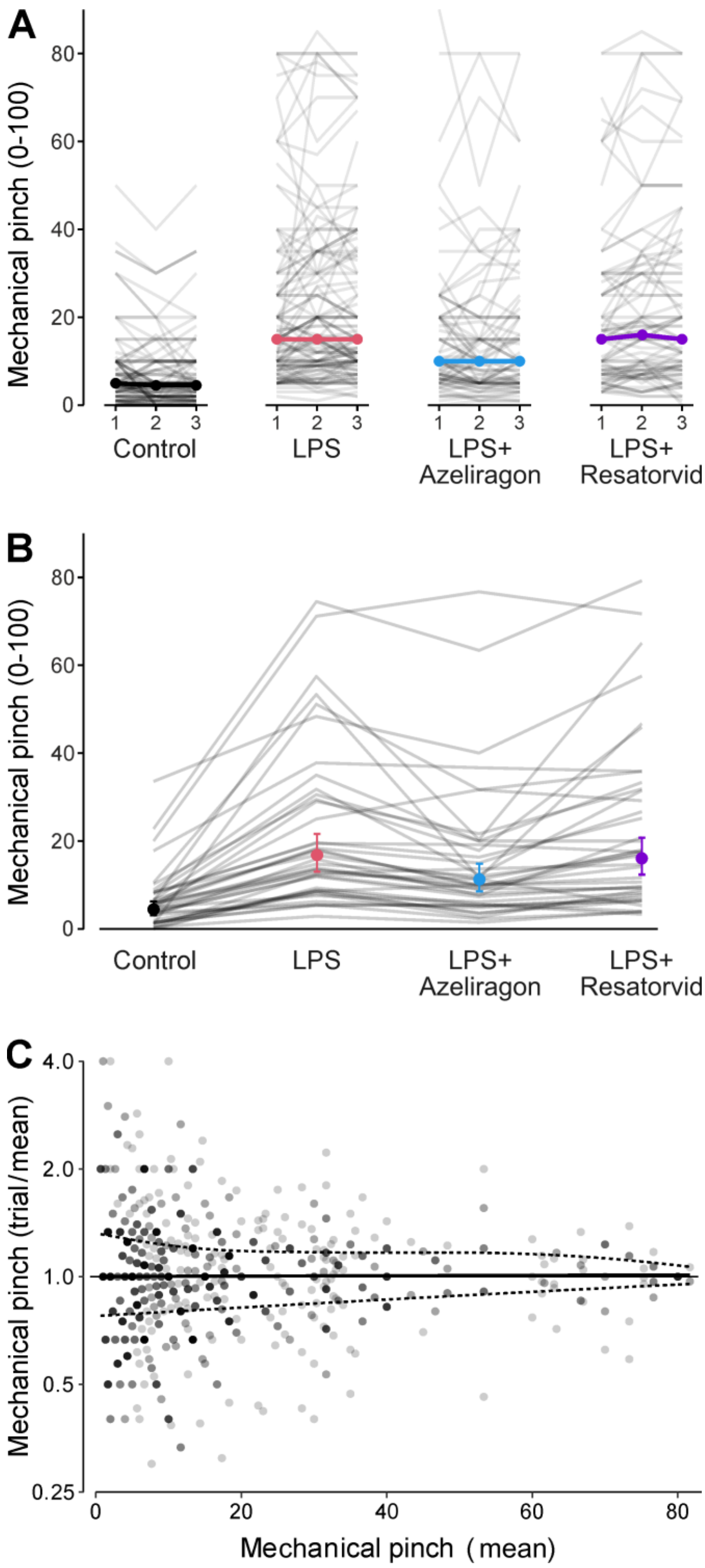

Supplementary Figure 2. A) Ratings for the three subsequent pinches for all 40 subjects were consistent and without a trend. Thick lines indicate the median rating. **B)** Per subject mechanical pinch pain ratings for the four different injection types, applied 4.5 h before testing. The intraclass correlations at the subject level was 0.69, indicating the ratings are a largely stable individual trait. Mean estimates and 95% CIs are overlayed. **C)** Distribution of individual mechanical pinch pain ratings, normalized to and plotted against the mean of three repetitions. Data points are 80% transparent, darker points result from overlays of multiple points. Half of the data are between the dotted lines, with higher variance for lower pain ratings. Source data are provided as a Source Data file.

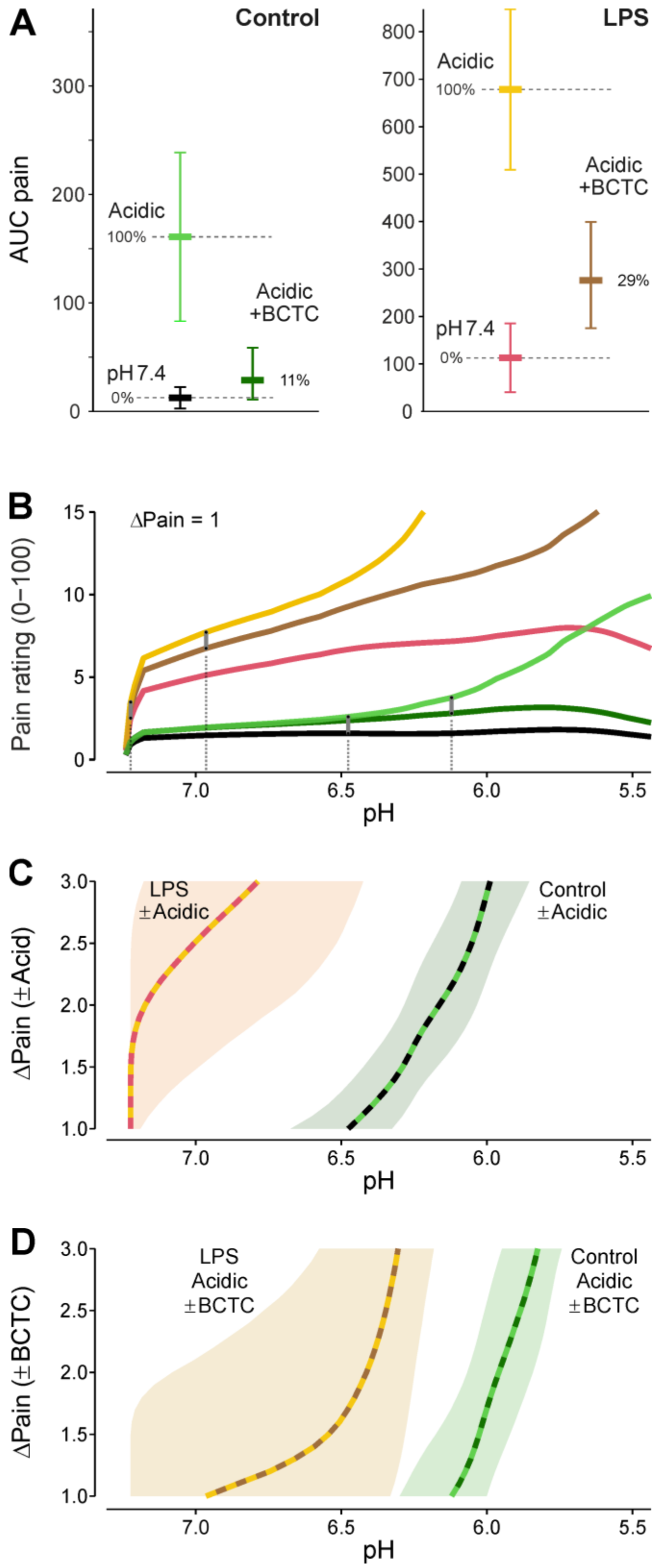

Supplementary Figure 3. TRPV1-dependence of acid-induced pain in naïve and inflamed skin. A) Mean estimates and 95% CIs from figures 3 and 6 for direct comparison. In naïve skin, the fraction of pain remaining in the presence of BCTC is 11%, the confidence interval includes complete inhibition. At 4.5 h after induction of inflammation by LPS, but the fraction remaining in the presence of BCTC is estimated at 29% on the added scale without (0%) and with LPS pretreatment (100%). **B)** Overlay of

generalized additive models to visualize the pH at which the pain rating differed by 1 unit. For the groups with pH 7.4, the time of the rating was projected onto the corresponding pH of the increasingly acidic groups. **C)** Without inflammation, increasingly acidic compared to pH 7.4 stimulation increased the rating by about 1 at a pH of 6.5 (dashed green-black, as green is used for acid and black for the control). In LPS-induced inflammation, a difference of 1 was present from pH 7.2. Also for higher increases of the pain rating, a 0.7–1.0 higher pH was sufficient in inflammation for a similar increase in pain compared to non-inflamed control. Data are visualized from 1 as a meaningful difference up to a delta of 3, which is available for all posterior samples. For this range of ΔPain values, the pointwise determined 95% confidence interval bands for the pH levels are provided. **D)** Without inflammation, the TRPV1-dependent acidic pain is indicated by comparing the groups with and without BCTC, which show a rating difference of about 1 at a pH of 6.1. In LPS-induced inflammation, the same difference of 1 was present pH 7.0, demonstrating inflammatory TRPV1 sensitisation. Source data are provided as a Source Data file.

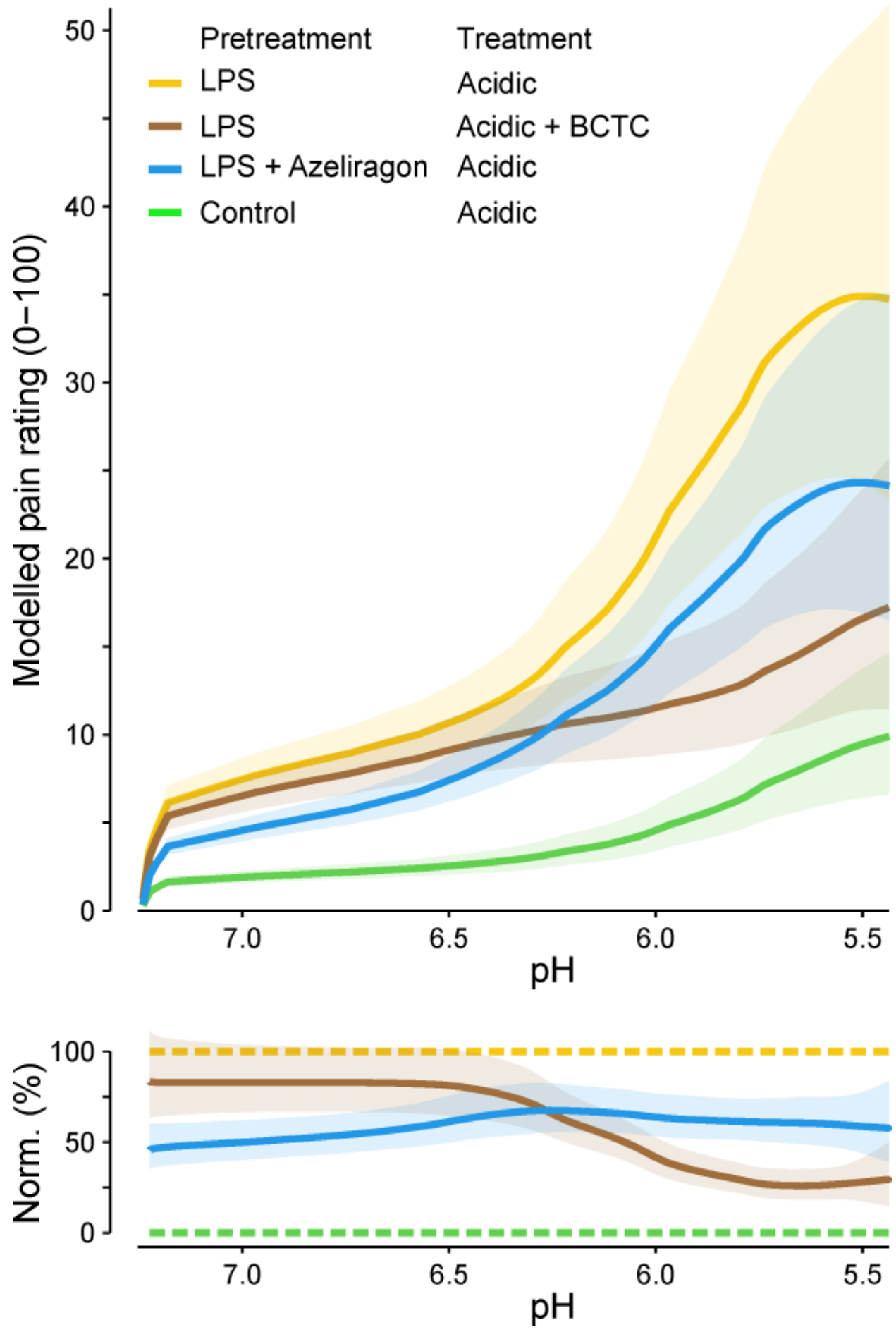

Supplementary Figure 4. TRPV1 and RAGE contribute differently to inflammatory sensitisation. Pain ratings were modelled by a zero-anchored general additive model, which provided 95% CI bands of the mean estimates. The pH-dependence of pain in inflamed skin with vs. without azeliragon pretreatment shows a difference throughout the whole pH range. This is apparent in the bottom panel, where the ratings are normalized to acid-induced pain 4.5 h after control (0%) and LPS (100%), suggesting a rather uniform action of azeliragon against inflammatory sensitisation. In contrast, the addition of BCTC only acts below a pH threshold against the sensitized TRPV1 receptor. Source data are provided as a Source Data file.

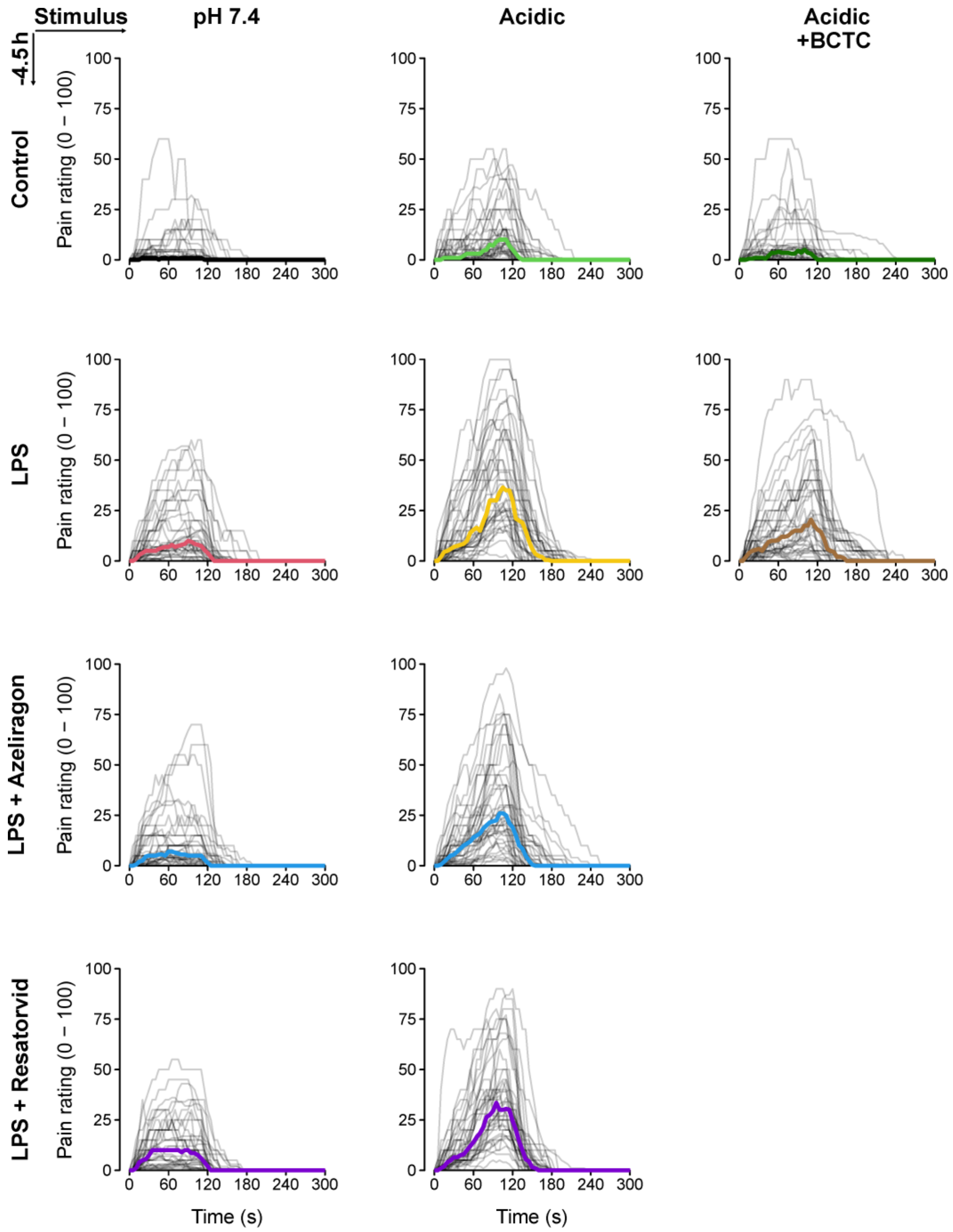

Supplementary Figure 5. Raw data and medians for all injection pain ratings of the main study. Full time courses of pain ratings of all experimental groups. All injections lasted 110 s. The individual traces of the 40 participants are provided in light grey, the median as a solid line. Rows have the same pretreatment 4.5 h before testing, columns have the same pain-inducing injection. Source data are provided as a Source Data file.

CONSORT 2025 checklist of information to include when reporting a randomised trial*

Section / Topic	No	CONSORT 2025 checklist item description	Reported on page no.
**Title and abstract**			
Title and structured abstract	1a	Identification as a randomised trial	2
	1b	Structured summary of the trial design, methods, results, and conclusions	2
**Open science**			
Trial registration	2	Name of trial registry, identifying number (with URL) and date of registration	15
Protocol and statistical analysis plan	3	Where the trial protocol and statistical analysis plan can be accessed	31 Supplement
Data sharing	4	Where and how the individual de-identified participant data (including data dictionary), statistical code and any other materials can be accessed	31 Supplement
Funding and conflicts of interest	5a	Sources of funding and other support (e.g., supply of drugs), and role of funders in the design, conduct, analysis and reporting of the trial	38
	5b	Financial and other conflicts of interest of the manuscript authors	38
**Introduction**			
Background and rationale	6	Scientific background and rationale	3-4
Objectives	7	Specific objectives related to benefits and harms	N/A
**Methods**			
Patient and public involvement	8	Details of patient or public involvement in the design, conduct and reporting of the trial	N/A
Trial design	9	Description of trial design including type of trial (e.g., parallel group, crossover), allocation ratio, and framework (e.g., superiority, equivalence, non-inferiority, exploratory)	2,21
Changes to trial protocol	10	Important changes to the trial after it commenced including any outcomes or analyses that were not prespecified, with reason	None
Trial setting	11	Settings (e.g., community, hospital) and locations (e.g., countries, sites) where the trial was conducted	15
Eligibility criteria	12a	Eligibility criteria for participants	21
	12b	If applicable, eligibility criteria for sites and for individuals delivering the interventions (e.g., surgeons, physiotherapists)	N/A

Intervention and comparator	13	Intervention and comparator with sufficient details to allow replication. If relevant, where additional materials describing the intervention and comparator (e.g., intervention manual) can be accessed	15-19
Outcomes	14	Pre-specified primary and secondary outcomes, including the specific measurement variable (e.g., systolic blood pressure), analysis metric (e.g., change from baseline, final value, time to event), method of aggregation (e.g., median, proportion), and time point for each outcome	16,24
Harms	15	How harms were defined and assessed (e.g., systematically, non-systematically)	15
Sample size	16a	How sample size was determined, including all assumptions supporting the sample size calculation	21
	16b	Explanation of any interim analyses and stopping guidelines	N/A
Randomisation:			
Sequence generation	17a	Who generated the random allocation sequence and the method used	21
	17b	Type of randomisation and details of any restriction (e.g., stratification, blocking and block size)	21
Allocation concealment mechanism	18	Mechanism used to implement the random allocation sequence (e.g., central computer/telephone; sequentially numbered, opaque, sealed containers), describing any steps to conceal the sequence until interventions were assigned	21
Implementation	19	Whether the personnel who enrolled and those who assigned participants to the interventions had access to the random allocation sequence	21
Blinding	20a	Who was blinded after assignment to interventions (e.g., participants, care providers, outcome assessors, data analysts)	19
	20b	If blinded, how blinding was achieved and description of the similarity of interventions	19
Statistical methods	21a	Statistical methods used to compare groups for primary and secondary outcomes, including harms	24-25
	21b	Definition of who is included in each analysis (e.g., all randomised participants), and in which group	N/A
	21c	How missing data were handled in the analysis	N/A
	21d	Methods for any additional analyses (e.g., subgroup and sensitivity analyses), distinguishing prespecified from post-hoc	25-31
**Results**			
Participant flow, including flow diagram	22a	For each group, the numbers of participants who were randomly assigned, received intended intervention, and were analysed for the primary outcome	5
	22b	For each group, losses and exclusions after randomisation, together with reasons	5
Recruitment	23a	Dates defining the periods of recruitment and follow-up for outcomes of benefits and harms	15
	23b	If relevant, why the trial ended or was stopped	N/A

Intervention and comparator delivery	24a	Intervention and comparator as they were actually administered (e.g., where appropriate, who delivered the intervention/comparator, how participants adhered, whether they were delivered as intended [fidelity])	15-19
	24b	Concomitant care received during the trial for each group	N/A
Baseline data	25	A table showing baseline demographic and clinical characteristics for each group	5
Numbers analysed, outcomes and estimation	26	For each primary and secondary outcome, by group:<br>● the number of participants included in the analysis<br>● the number of participants with available data at the outcome time point<br>● result for each group, and the estimated effect size and its precision (such as 95% confidence interval)<br>● for binary outcomes, presentation of both absolute and relative effect size	5
Harms	27	All harms or unintended events in each group	9
Ancillary analyses	28	Any other analyses performed, including subgroup and sensitivity analyses, distinguishing pre-specified from post-hoc	6-8
**Discussion**			
Interpretation	29	Interpretation consistent with results, balancing benefits and harms, and considering other relevant evidence	9-13
Limitations	30	Trial limitations, addressing sources of potential bias, imprecision, generalisability, and, if relevant, multiplicity of analyses	13-14

*We strongly recommend reading this statement in conjunction with the CONSORT 2025 Explanation and Elaboration and/or the CONSORT 2025 Expanded Checklist for important clarifications on all the items. We also recommend reading relevant CONSORT extensions. See www.consort-spirit.org.

Citation: Hopewell S, Chan AW, Collins GS, Hróbjartsson A, Moher D, Schulz KF, et al. CONSORT 2025 Statement: updated guideline for reporting randomised trials. BMJ. 2025; 388:e081123. https://dx.doi.org/10.1136/bmj-2024-081123.

© 2025 Hopewell et al. This is an Open Access article distributed under the terms of the Creative Commons Attribution License (https://creativecommons.org/licenses/by/4.0/), which permits unrestricted use, distribution, and reproduction in any medium, provided the original work is properly cited.

STUDY PROTOCOL

Mechanisms of pain hypersensitivity in a human skin inflammation model

a double-blind, randomized, placebo-controlled crossover study

Applicant and sponsor

Univ.-Prof. Dr. med. habil. Dr. med. Michael Fischer

Protocol authors

Univ.-Prof. Dr. med. habil. Dr. med. Michael Fischer

Dr.med.univ. Felix J. Resch

Priv.-Doz. Dr.med.univ. Dr.rer.nat. Stefan Heber

Dr.med.univ. Sybille Pramhas

Principal investigator

Ao.Univ.-Prof. Dr.med.univ. Sabine Sator

Table of Contents

Background and Objective
Study objective and hypotheses
Study design
Study population
Methodology and experimental procedures
Statistical methodology and analysis
Ethical and legal aspects

Background and Objective

A human model of skin inflammation

Inflammation can be considered one of the most important causes of acute and chronic pain conditions. To develop new treatment strategies for inflammatory pain, the underlying mechanisms have to be studied using appropriate models. Accessibility is one of the main preconditions in the study of pain in humans, making the skin most appealing. Therefore, the purpose of this pilot study is to establish a human model reflecting the proinflammatory aspects of skin infection and characterize it in terms of inflammation-induced allodynia/hyperalgesia using multiple painful stimuli.

Skin infections can clinically be assigned to the spectrum of skin and soft tissue infections (SSTIs). A variety of gram-positive and gram-negative bacteria are known to cause SSTIs, often multiple pathogens are isolated from the same site. SSTIs cover a wide spectrum of infections, including mostly harmless superficial infections (e.g. furuncles) or possibly life-threatening deep infections (e.g. necrotizing fasciitis). Lipopolysaccharide (LPS) is a highly immunogenic component of gram-negative bacteria, spanning their cell walls. In fact, for mimicking infection with gram-negative bacteria, LPS is established to trigger an inflammatory response in healthy subjects (Bahador 2007). Whereas most published studies investigated intravenous LPS challenges (dose range 0.06 - 4 ng/kg, which culminate in total systemic doses of up to ~300 ng), LPS has also been applied intradermally with doses of 5-15 ng per injection (total doses of up to 45 ng), leading to small-diameter inflammatory lesions that completely subside within 48 h (Figure 1). Importantly, in these studies, no systemic signs or symptoms were observed (Basran 2013, Buters 2022a, Buters 2022b). Importantly, LPS redistribution has been investigated. LPS is extraordinarily slowly redistributed from an injection spot. It takes several days until 50% are removed. When it has reached the circulation, it is removed by the liver. Therefore, after a peripheral LPS injection at 'no point was more than 1% of the injected dose found in the plasma' (Lu 2011). Thus, the topical LPS challenge can be considered an established and safe model of inflammation and is suitable for applying painful stimuli. The main receptor for LPS is Toll-like receptor 4 (TLR4) expressed on cells of the innate immune system. Binding of LPS to the receptor for advanced glycation end products (RAGE) has also been suggested to contribute to some of the immunogenic effects (Yamamoto 2011) and it has been shown that LPS can directly sensitize sensory neurons through RAGE *in vitro* (Nair 2021). The RAGE antagonist Azeliragon, which has also undergone Phase III trials, will be used to probe the involvement of this pathway. Resolving the significance of the investigated signalling pathways could have an influence on the selection of therapeutic agents in the future.

An exploratory pilot study was recently conducted by us to illustrate the time course of hypersensitivity, to elaborate the maximum thereof and to provide the basis for calculating the sample size for this study. The main purpose of this study is to elucidate whether the proinflammatory effects of LPS are also partially mediated through a RAGE-dependent signaling pathway and thus contribute to hyperalgesia.

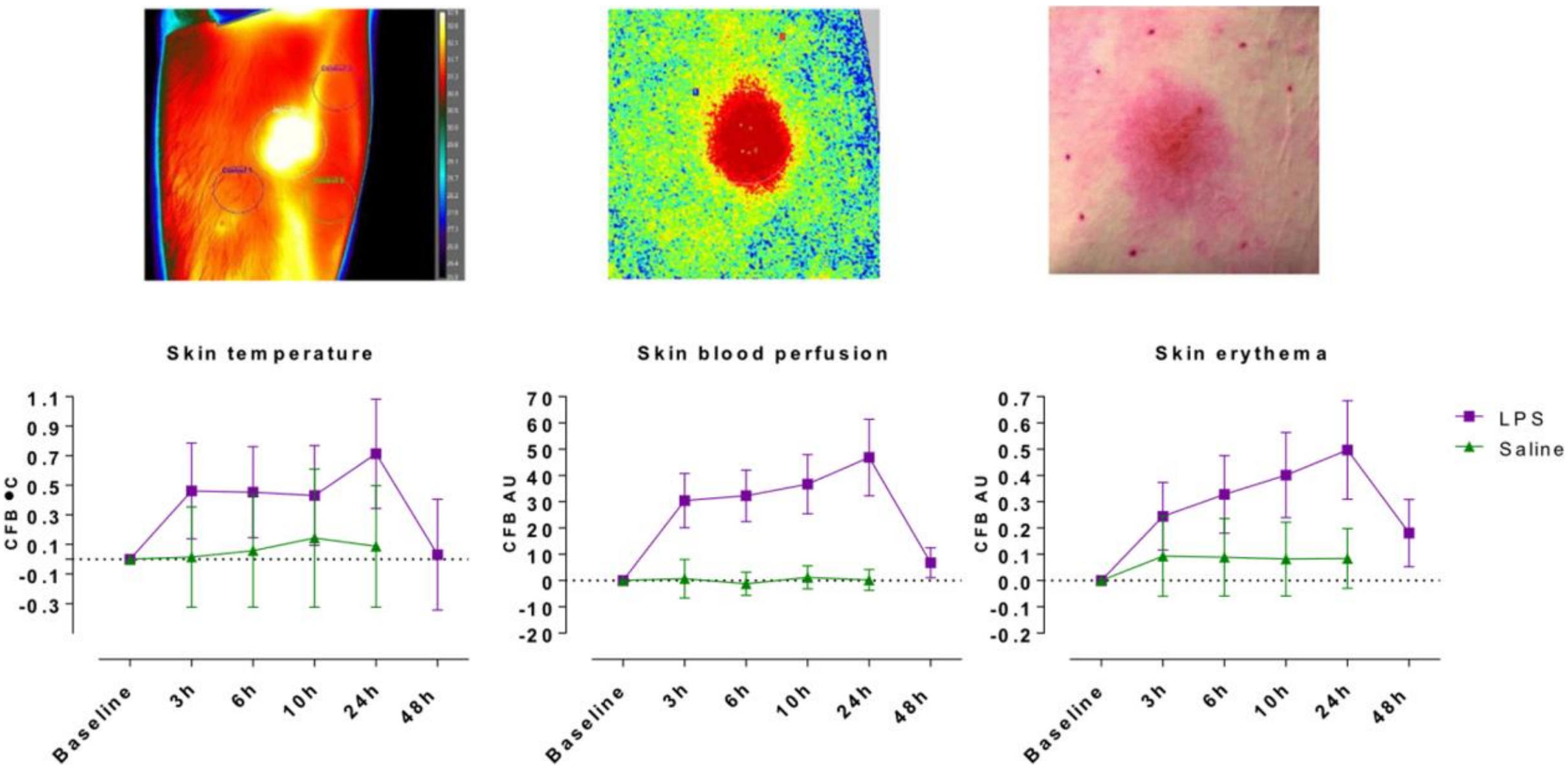

Figure 1: Skin inflammation induced by LPS. Upper panel: Representative images and quantification of 5 ng LPS induced inflammatory response using thermography, laser speckle contrast imaging and erythema photo analysis. Lower panel: Quantification of the respective time courses. Adapted from Buters et al., Br. J. Clin. Pharmacol., 2021.

Human pain models

During inflammatory conditions allodynia/hyperalgesia can occur in response to certain stimuli. Upon inflammation the skin can get allodynic to thermal stimuli (e.g. taking a warm shower when having a solar erythema) and mechanical stimuli (e.g. slightly touching a finger affected by paronychia). In two of our previous studies, we investigated the role of acidosis in inducing pain in native, non-inflamed skin (Schwarz 2017, Heber 2020). However, it remains to be elucidated whether, and if so how, an acidic stimulus leads to aggravation of the perceived pain in inflamed skin. The following chapters are intended for introducing some considerations about the pain models planned to be used in this study.

Model of mechanical allodynia

Another well-known characteristic of inflamed skin is mechanical allodynia, which describes a painful sensation upon applying a normally not painful mechanical stimulus. The standardized protocol for QST also includes the determination of the mechanical pain threshold, i.e. the

threshold at which a defined mechanical stimulus applied to the skin elicits pain. This is mostly done by using multiple pin-prick mechanical stimulators that exert different amounts of force onto a small skin area (Rolke 2006). However, even the highest force usually being used in the clinical setting by pin-pricks (512 mN) was not painful and did not allow discriminating LPS versus control injection. Therefore, the pin-prick model was considered unsuitable to detect this type of allodynia. In contrast, slight mechanical distension or compression allowed to reliably cause allodynic perception and therefore to identify LPS- versus control-injected skin spots in a blinded setting.

Model of acid induced pain

In inflammatory conditions, tissue acidosis is thought to contribute to the associated pain. Therefore, addressing pathways leading to acid induced pain represents a promising strategy for the treatment of inflammatory pain. There are several ion channels known to be sensitive to acidification in *in vitro* studies or animal models. For humans, we demonstrated that the pain induced by acidification to pH 6 is mediated by TRPV1 (Heber 2020). In these experiments the pH was reduced stepwise, with little increase in pain due to pH 7.0 and 6.5. In contrast, a further decrease to pH 6.0 induced pain (Figure 2A), which was abolished when the injected solution contained BCTC (Figure 2B), suggesting TRPV1 as the primary acid sensor in healthy human skin. It remains to be elucidated, whether under inflammatory conditions an acidic stimulus leads to aggravation of pain and whether TRPV1 is still the main sensor or if other channels contribute as well.

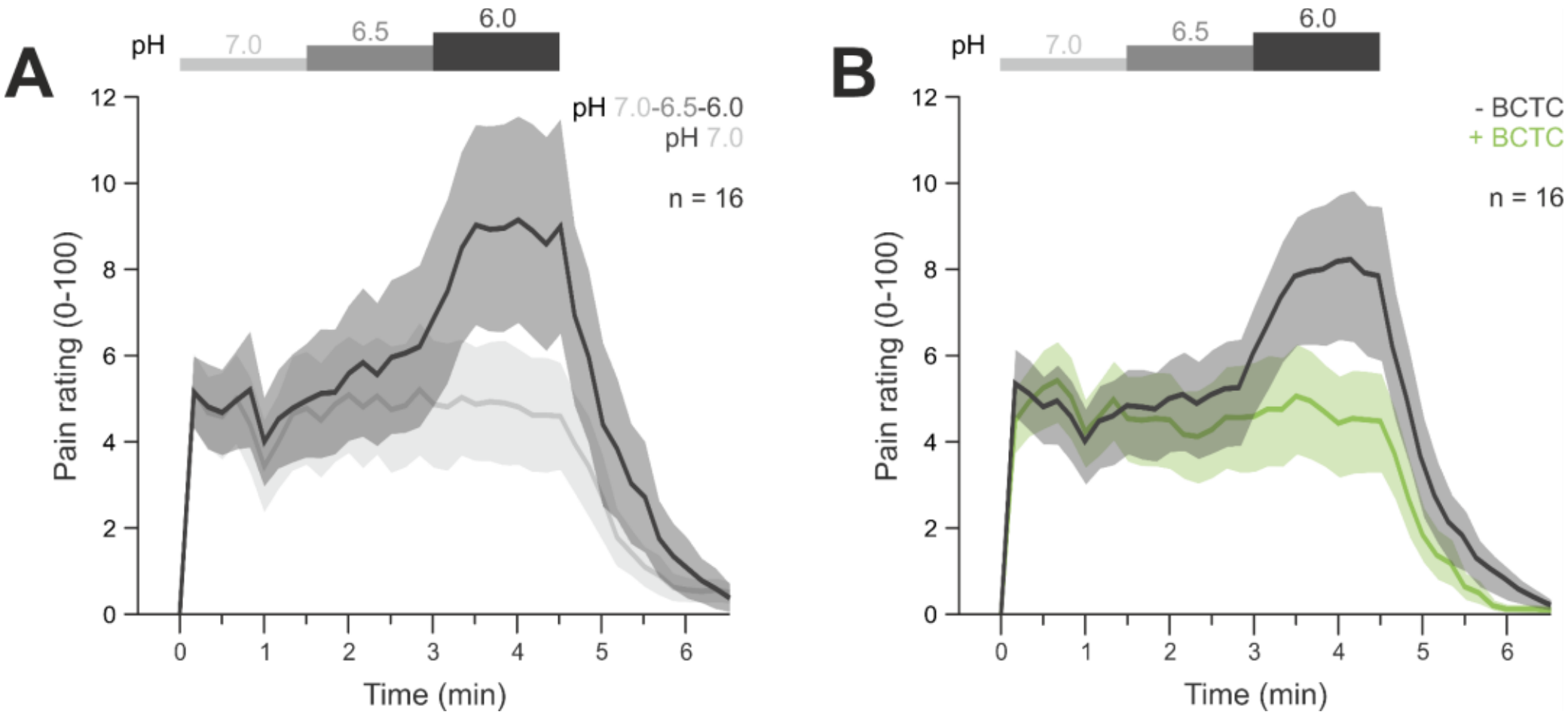

Figure 2: BCTC inhibits pH 6.0-induced pain. (A) Pain rating time courses for intradermal injection into the forearm, with a constant pH 7.0 or with a stepwise decreasing pH. During injection volunteers rated pain every 5 seconds on a numeric pain rating scale (0-100). **(B)** Addition of TRPV1 antagonist BCTC 1 µM to the injection solution abolished pain induced by pH 6.0. Figure adapted from Heber et al., Pain, 2020.

For the current study, the experimental model was improved to generate a largely linear pH ramp. This has the benefit that the stimulus strength is not ordinal but continuously increasing. This generates a stimulus-response on an rational scale, and allows to determine an individual pH threshold and a shift thereof.

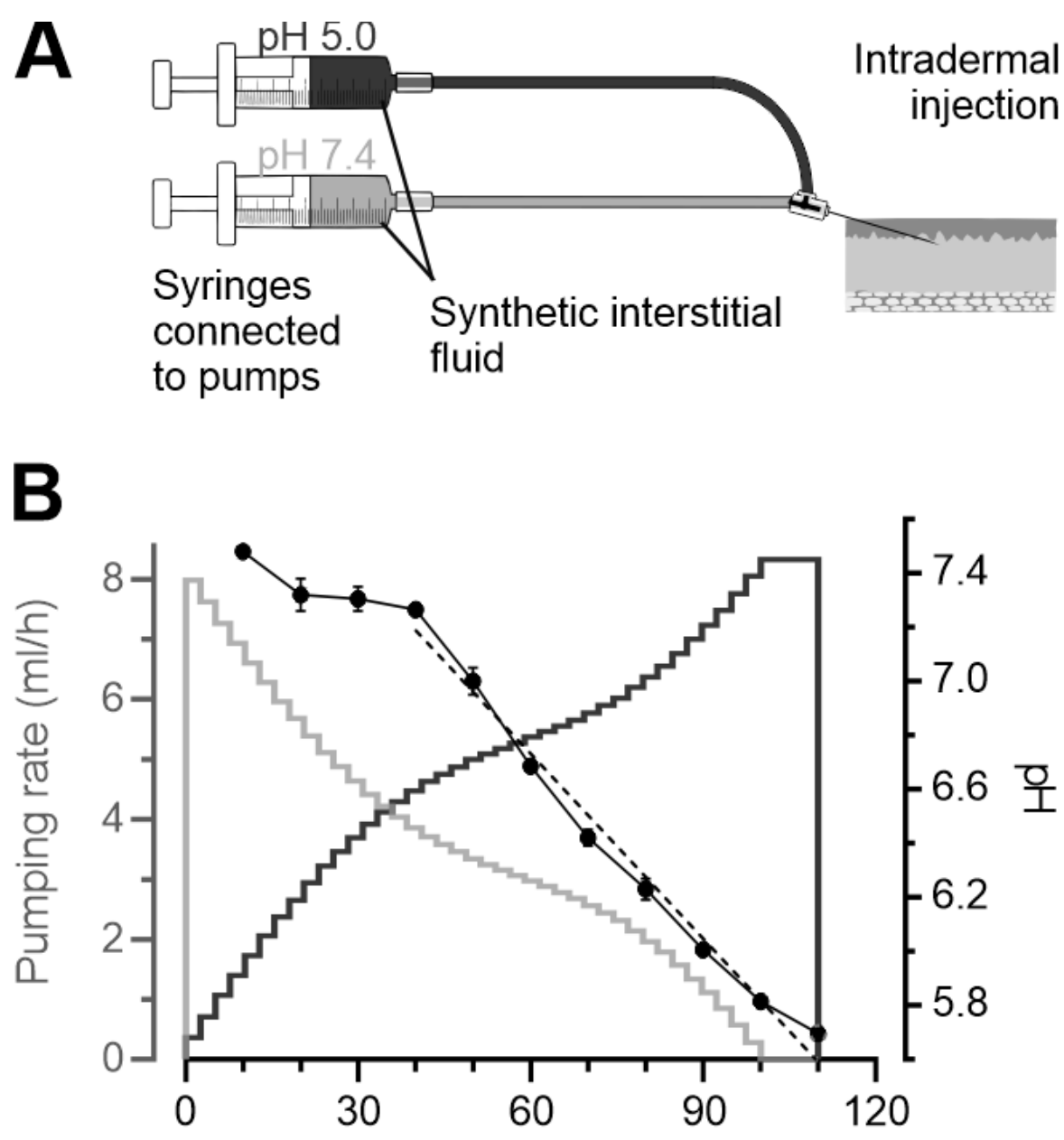

Figure 3: Acidosis ramp pain model. (A) Illustration of the setup. Perfusor lines connect two syringes connected to a 3-way stopcock attached to an intradermally placed 30G cannula. The solutions in the syringes have a pH of 7.4 and 5.0, respectively. **(B)** Stepwise adjustment of pumping rates of two programmable pumps results in an approximately linear decrease of the pH from 7.4 to 5.7, measured at the tip of the cannula (mean of two measurements ± range). This gradual pH change represents an improvement from the three pH steps of the prior study.

Results of the exploratory pilot study

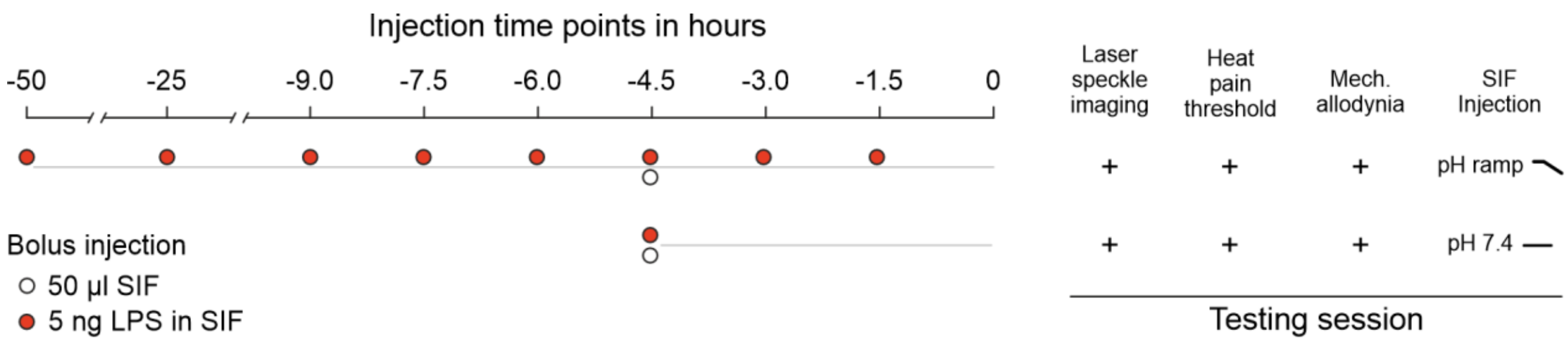

Figure 4: Overview of the protocol for the pilot study, conducted on 6 female and 6 male subjects.

Recently, an exploratory pilot study with 12 subjects was conducted to illustrate the time course of hypersensitivity towards the above-mentioned painful stimuli in the LPS inflammation model (as illustrated in Figure 4). This allowed us to evaluate the time course of

hypersensitivity following LPS injection and additionally provided the basis for calculating the sample size for the proposed amendment of the pilot study. We observed a time course with a peak at about 4.5 h and subsequent decline was observed for blood flow, the mechanical stimulus, and the acidic stimulus (Figure 5). The peak of pain hypersensitivity occurs at 4.5-6 h after LPS injection. Since AUCpain at the 4.5 hour time point do not significantly differ from those at the 6 hour time point, it is reasonable to choose the 4.5 hour time point for this study, as it already includes the necessary controls (Figure 4) and thus serves as the basis for sample size calculation for this study. The painful stimuli (mechanical and mild acidification) remain identical for this study, the heat pain threshold will be omitted.

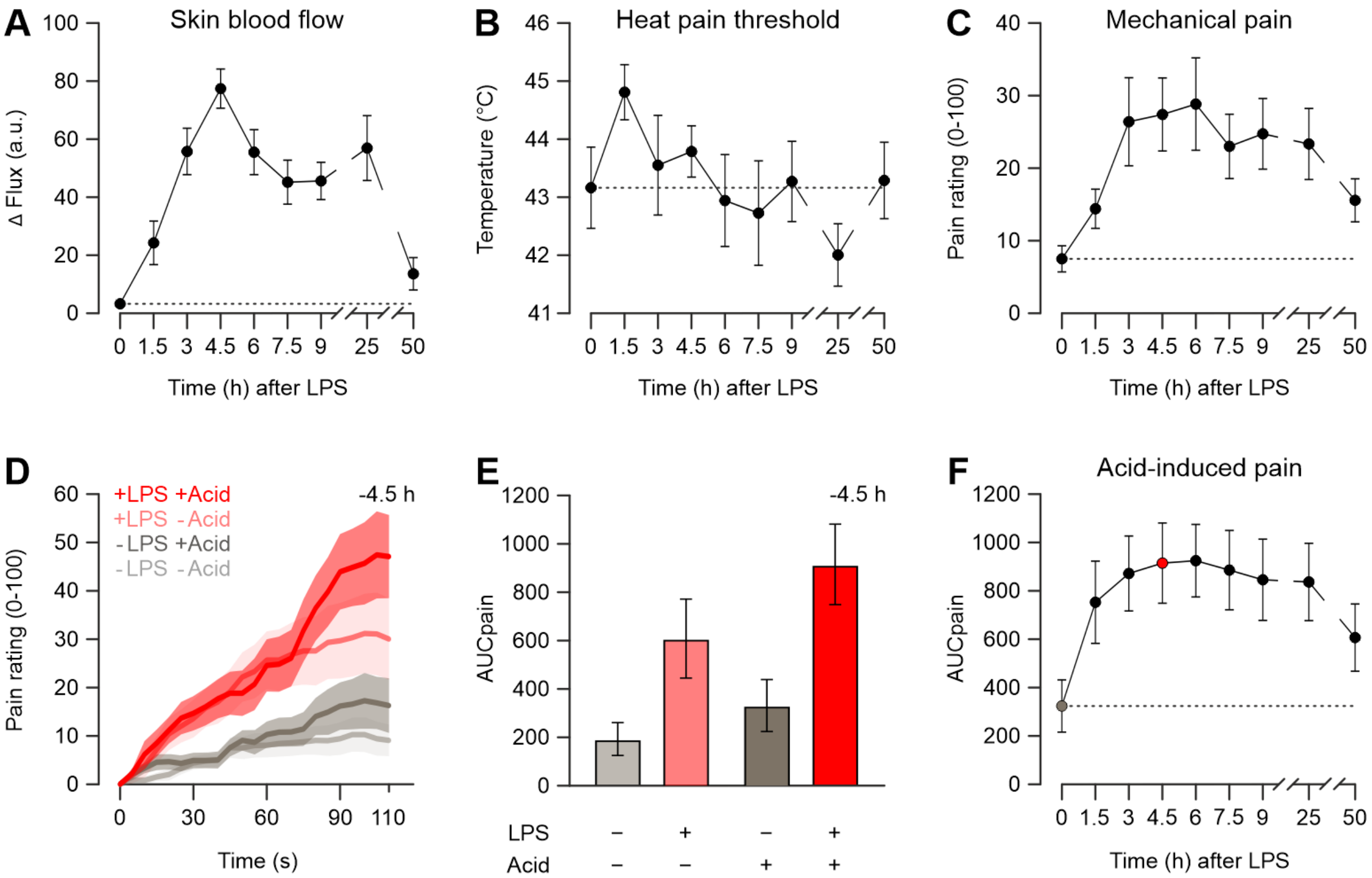

Figure 5: Time course of pain hypersensitivity in the LPS inflammation model. A) Hyperemia peaks at 4.5 h after LPS injection, with almost complete recovery observed at 50 h. **B)** The time course of the heat pain threshold is not suitable for further experimental use. **C)** Pain intensity of the mechanical stimulus application reaches a peak at 4.5-6 h. **D)** Intradermal injections are more painful when 1) the skin sites were pre-injected with LPS ('+LPS') and 2) when the pH decreases over the course of injections (from initial 7.4 to 5.7, '+Acid'). **E)** AUCs of the pain ratings (Panel D) in the interval of 90 to 110 seconds. **F)** For the acidifying injections, the maximum AUC occurs at 4.5-6 h. All data points represent the mean of the 12 subjects ± SEM. For Panels A-C, data for 0 h are obtained from measurements of control-injected skin sites (-4.5 h, averaged value per subject from duplicate measurements). Data for 4.5 h are also averaged values from duplicate measurements.

Study objective and hypotheses

The aim of this study is to elucidate the mechanisms of LPS-induced hyperalgesia, which leads to three independent research questions.

Research Question 1 (Main Research Question): The Role of RAGE in LPS-induced inflammation

The mediation of immunogenic effects of LPS via TLR4 has long been established. The question remains whether parts of the proinflammatory effects of LPS are also mediated through an alternative RAGE-dependent signaling pathway, contributing to hyperalgesia. Antagonizing TLR4 serves as a reference. Blood flow provides an objective readout.

Primary Hypothesis: The degree of hyperemia is different in LPS-injected skin spots (3x) compared to LPS-injected skin spots where Azeliragon is co-injected (2x).

Secondary Hypothesis 1: Acidosis induced pain is different in LPS-injected skin spots (1x) compared to LPS-injected skin spots where Azeliragon is co-injected (1x).

Secondary Hypothesis 2: Mechanically induced pain is different in LPS-injected skin spots (3x) compared to LPS-injected skin spots where Azeliragon is co-injected (2x).

In the respective comparison groups, the contribution of TLR4 is measured with the antagonist Resatorvid as a reference.

Research Question 2 (Exploratory): Sensitization in the LPS Inflammation Model

This question concerns the pain induced by the injection of increasingly acidic (pH = 7.4 to 5.7) or neutral (pH = 7.4) solutions, differentiating between purely mechanical hyperalgesia and additional hyperalgesia due to acidosis. The pilot study with 12 subjects demonstrated that acidosis induced pain in the skin after LPS injection is 176% greater than after control injection ($p = 0.002$, paired t-test). The pilot study also shows that mechanically induced pain on the skin after LPS injection is 214% greater than after control injection ($p = 0.008$, paired t-test). However, it remains unclear whether the difference between mechanically and acidosis induced pain changes after LPS injection.

Hypothesis (Exploratory): The pain induced by acidosis in addition to mechanically induced pain (by injection into the skin) is greater after LPS injection than after control injection.

Research Question 3 (Exploratory): The Role of TRPV1 in acidosis induced Pain in the LPS Inflammation Model

The question is whether the proportion of acidosis-induced pain attributable to TRPV1 activation is altered by LPS-induced inflammation.

Hypothesis (Exploratory): The inhibition of acidosis-induced pain by adding BCTC to the injection solution is greater after LPS injection than after control injection.

Study design

This study is intended for non-therapeutic biomedical research on humans (basic research). It is designed as a double-blind, randomized, placebo-controlled crossover study. The study is performed at the Division of Special Anesthesia and Pain Medicine (AKH 9.i2.04).

Study population

Participants are recruited using the provided document entitled “Proband:innen für Studie gesucht - Schmerzreduktion durch Kaliumkanäle”. The experimental details will be explained to the participants. Subjects, which meet the inclusion criteria, do not meet the exclusion criteria and provide written informed consent on the “TeilnehmerInneninformation und Einwilligungserklärung” will be included.

Inclusion criteria

- Age between 18 and 70 years
- Full legal capacity

To ensure an equal number of each sex in the study population, only volunteers of one sex will be included as soon as the number of subjects with the other sex has reached half of the target sample size of 40 subjects. The total number of participants is 45, which accmodates for up to 10% dropouts. The study ends when 40 subjects have completed the protocol.

Exclusion criteria

- Participant of another study, ongoing or within the last four weeks
- Current medication intake (except hormonal contraception) or drug abuse
- Female subjects: Positive pregnancy test or breastfeeding

- Body temperature above 38°C, diagnostically verified
- Known allergic diseases, in particular asthmatic disorders and skin diseases
- Sensory deficit, skin disease or hematoma of unknown origin in the physical examination of the test site
- Symptoms indicating respiratory tract infection

Study duration

Overall the study is assumed to take about one year.

Withdrawal of subjects

Subjects can voluntarily withdraw from the study at any time, for any reason and without any consequences.

Methodology and experimental procedures

Females of childbearing potential

First, for female participants, pregnancy is excluded by a pregnancy test. In case of a positive test, the participant is excluded and will be compensated for travelling.

Experimental procedures

Familiarization: Before induction of skin inflammation by LPS injection, subjects will be familiarized with the subsequent pain stimuli (mechanical and acid) at two distinct native skin spots. The procedures are fully identical to the test stimulation for mechanical pain and acid-induced pain. The only difference is that it serves only for the subject to get to know the procedure. These stimulations will not be considered in the analysis.

Thereafter, LPS ± substances are injected. After a period of 4.5 h later, noxious stimuli as in the pilot study are applied (Figure 6). The procedure for determining mechanical allodynia and acidosis-induced pain remains unchanged; two of the injection solutions will additionally contain BCTC.

Time point 1: Induction of skin inflammation resembling bacterial infection

To trigger an inflammatory response resembling infection with gram-negative bacteria in the skin of human subjects, 5 ng of LPS dissolved in 50 µl of SIF, will be injected superficially into premarked skin spots of the volar forearm 4.5h before the application of noxious stimuli using a 30G syringe (BD, Micro-Fine U-100, 0,3x8 mm). The dose of 5 ng of LPS is based on literature (https://pubmed.ncbi.nlm.nih.gov/34293819/), which was validated in pilot experiments. Each participant receives 10 injections, with 3 control injections and 7 injections

containing LPS. In two LPS injections, the RAGE antagonist Azeliragon (100µM) is added, and in another two the TLR4 antagonist Resatorvid (10µM).

Time point 2: Quantification of skin perfusion

The degree of hyperemia is quantified by using laser speckle imaging (moorFLPI-2, Moor Instruments Ltd). It can be assumed that the inflamed skin area itself does not systematically vary between spots and subjects. In contrast to that, the distance at which the images are taken may vary between subjects. Therefore, a circular adhesive sticker of a diameter of 2 cm is placed in the center of both forearms as a reference. The subject is then asked not to move the forearm for a few seconds to acquire blood flow and a color image. The same procedure is repeated on the contralateral forearm.

Time point 2: Model of mechanical allodynia

Mechanical stimulation of the skin is realized by the use of self-closing cross-over tweezers. The tweezers are customized to have an maximal opening diameter of 6 mm, which fits the total area of erythema arising from the inflammation model used. For stimulation, the levers are opened by squeezing, gently put onto the test site, and closed by releasing. The self-closing property of the tweezers allows applying a standardized pinch to the skin, intensity of the perceived pain is rated by the subject thereafter. All pain ratings are provided on a scale 0-100, where 0 indicates not pain and 100 the maximal imaginable pain. The subjects are positioned and instructed not to look at the test site. For each spot the mechanical sensitivity is tested three times.

Time point 2: Model of acid induced pain

To expose human subjects to a defined nonhazardous intradermal acidic stimulus, the protocols of our two recent studies (continuous injection of constant pHs of 7.0, 6.5, 6.0 or 4.3, respectively) have been further developed to provide a linear decrease from a pH of 7.4 to a pH of 5.7. This is realized by two syringes (Braun, Injekt Luer Lock Solo, 5 ml) connected via perfusor lines (Braun, Original Perfusor Line, 25 cm) to a 3-way stopcock (Braun, Discofix 3SC), where the solutions are being mixed before intradermal injection through a 30G cannula (Braun, Sterican, 0.3 x 12 mm; Figure 3A). The syringes contain SIF with a pH of 7.4 and 5.0, respectively. Over a period of 110 seconds, two programmable pumps (World precision instruments, Sarasota, FL) stepwise adapt their infusion rates to generate a linear decrease from a pH of 7.4 to a pH of 5.7 (Figure 3B). The summarized total infusion rate is fixed at 30 ml/h throughout the protocol, which results in a total volume of 0.92 ml administered per injection. As in previous studies, pain is rated every five seconds on a numerical pain rating scale ranging from 0 to 100, whereas 0 represents no pain and 100 the maximum imaginable

pain. Stimulation will alternatingly use both forearms. First, the cannula is intradermally inserted into the respective spot (inflamed or non-inflamed) on the volar forearm of the subject and further fixed with Leukoplast (BSN medical GmbH) to prevent slipping of the stopcock. The infusion and the stopwatch are started simultaneously when no pain (= 0) is reported after cannula insertion. Pain is rated every five seconds until the subject reports no pain for a period of 30 consecutive seconds. A non-acidic stimulus is obtained by filling both syringes with solutions of a pH of 7.4.

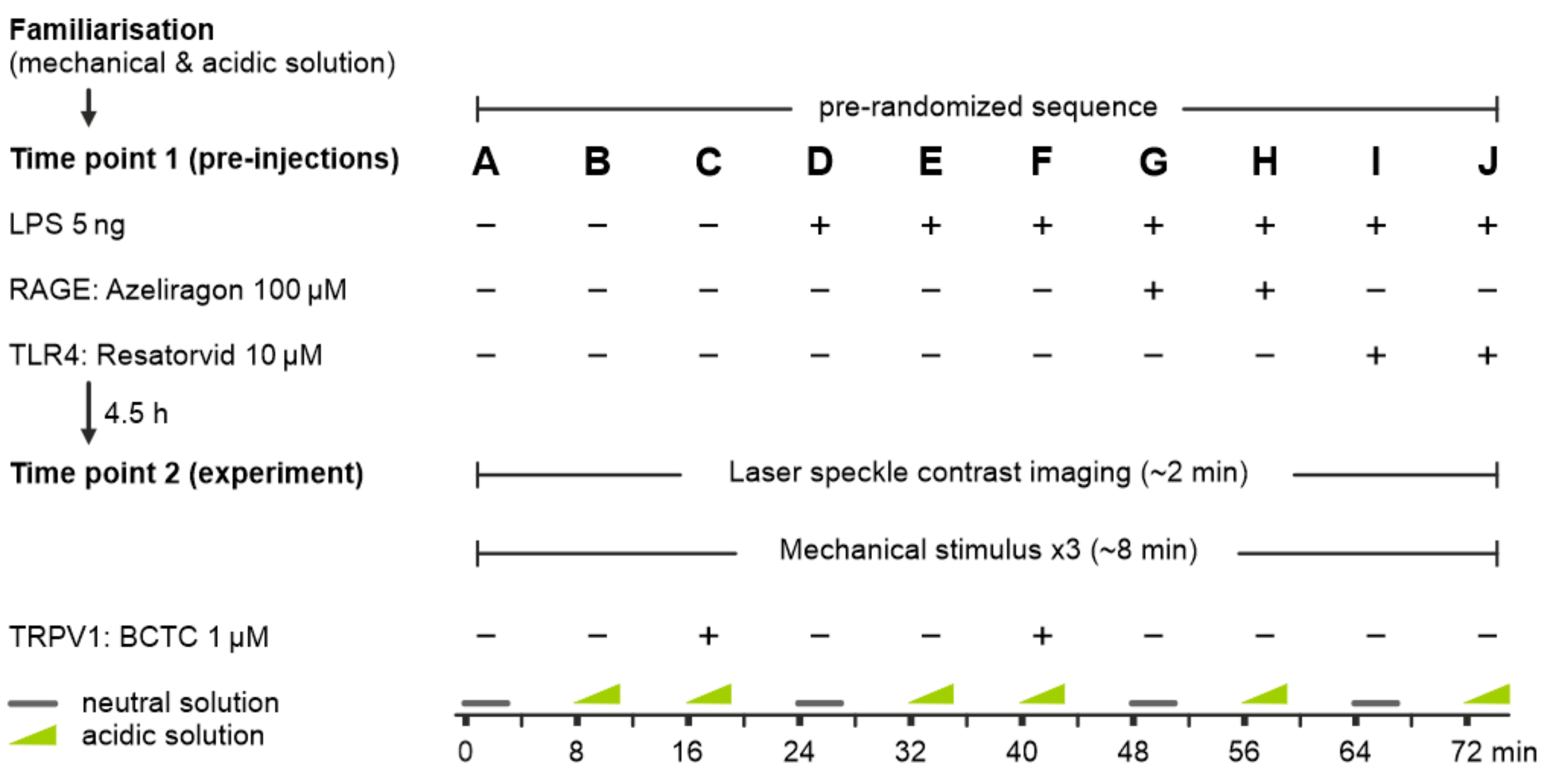

Figure 6: Overview of the experimental procedures.

Applied substances, dosage and administration

Synthetic interstitial fluid (SIF)

To closely mimic extracellular fluid, we previously used a synthetic interstitial fluid (SIF) (https://pubmed.ncbi.nlm.nih.gov/5781321/). To reach a physiological buffer capacity, histidine (pK_a=6.0) is added at 22.0 mM. This SIF variant allows for a stable pH in the range of 5.0 to 7.4 and contains the following (in mmol/l): NaCl 113.8, KCl 3.5, Na_2HPO_4 1.7, $MgSO_2 \times 7H_2O$ 0.7, sodium gluconate 9.6, glucose 5.0, sucrose 7.6, $CaCl_2 \times 2H_2O$ 1.5, Histidine 22.0. The calculated osmolarity is 300 mosmol/l. All fluids injected are diluted in SIF adjusted to a pH of 7.4. For the acid induced pain model, SIF is adjusted to a pH of 7.4 and 5.0, respectively.

Lipopolysaccharide (LPS)

Lipopolysaccharide (LPS; Molecular formula: $C_{205}H_{366}N_3O_{117}P_5$; 4900 g/mol) is a major constituent of the cell wall of gram-negative bacteria. It is acquired from List Biological Laboratories as a highly purified molecule obtained from Escherichia coli O113, which has

been shown to be safe in humans (https://pubmed.ncbi.nlm.nih.gov/10191237/). Notably, List Biological Laboratories was commissioned by the NIH to manufacture GMP grade reference endotoxin (https://pubmed.ncbi.nlm.nih.gov/30782041/). According to the corresponding author, the same source has been used for two of the above mentioned studies on intradermal LPS challenge (https://pubmed.ncbi.nlm.nih.gov/34293819/ https://pubmed.ncbi.nlm.nih.gov/34935141/). In this study, applying a dose of 5 ng per injection, 7 injections of LPS will result in a total dose of 35 ng, which is in the range of total doses used in previous intradermal trials and far below the total doses injected in intravenous trials (up to 300 ng). None of our preliminary experiments with repeated injections of LPS have indicated systemic adverse effects.

Resatorvid

This TLR4 antagonist is co-injected with LPS at a dose of 2 x 0.18 µg = 0.36 µg. Resatorvid has undergone Phase III trials (daily doses of 1.2 or 2.4 mg/kg intravenously for 4 days, equivalent to daily doses of 84 or 168 mg for a person weighing 70 kg, tested on 92 or 93 study participants, https://clinicaltrials.gov/study/NCT00143611). The daily total dose used in those trials exceeds the dose used here by nearly 500,000-fold. Results of the study have been published (https://pubmed.ncbi.nlm.nih.gov/20562702/). The side effect of methemoglobinemia: “In the TAK-242 1.2 mg/kg/day group, mean levels increased from 0.9% at baseline to 2.8% at 2 hrs” appears to be of limited relevance for the 500,000-fold lower dose. Therefore, it is assumed that there will be no adverse effects. Biological activity of Resatorvid against LPS activity has been shown using human TLR4 and its co-receptors CD14 and MD2 expressed in HEK293 cells (Kawamoto et al., Eur J Pharmacol. 2008; 584:40-8). The IC_{50} for LPS-induced NF-κB activation was found to be 110 nM. Assuming a dilution factor of about 10-fold upon intradermal injection and aiming for a receptor occupancy of at least 90%, using ~ 100-fold the IC_{50} was considered reasonable. Resatorvid (CAS 243984-11-4) is obtained from MedChemExpress, dissolved as 1 mM stock solution in DMSO and diluted to the final concentration of 10 µM, resulting in a solution containing 1% DMSO.

Azeliragon

This RAGE antagonist is co-injected with LPS at a dose of 2 x 2.66 µg = 5.32 µg. Azeliragon has also undergone Phase III trials (daily dose of 5 mg orally for 18 months in ~440 study participants), where the daily total dose used exceeded the dose used here by nearly 1,000-fold (https://www.clinicaltrials.gov/study/NCT02080364). It is also assumed here that there will be no adverse effects. Biological activity of 4 mg/kg intraperitoneal Azeliragon has been demonstrated by the reduction of LPS-induced airway inflammation

(https://pubmed.ncbi.nlm.nih.gov/33548580/). Binding of the prototypical RAGE agonist S100b is inhibited by Azeliragon with an IC_{50} of ~1 µM (https://patents.google.com/patent/WO2005000295A1/en?oq=WO2005000295). Assuming a dilution factor of about 10-fold upon intradermal injection and aiming for a receptor occupancy of at least 90%, using ~ 100-fold the IC_{50} was considered reasonable. Azeliragon (CAS 603148-36-3) is obtained from Targetmol, dissolved as 10 mM stock solution in DMSO and diluted to the final concentration of 100 µM, resulting in a solution containing 1% DMSO.

BCTC (N-(4-tert-butylphenyl)-4-(3-chloropyridin-2-yl)piperazine-1-carboxamide)

This TRPV1 antagonist is administered at a total dose of 2 x 0.34 µg = 0.68 µg and added to the acidic solution. BCTC has been used in three previous studies at total doses of up to 3.36 µg per participant (https://pubmed.ncbi.nlm.nih.gov/28062311/ https://pubmed.ncbi.nlm.nih.gov/32107360/, Ethics application number: 1152/2020). No adverse effects were observed. The total dose of BCTC to be used here is approximately 20% of the previous maximal dose, so the risk of adverse effects is still considered very low. BCTC (CAS 393514-24-4) is obtained from Cayman Chemical and dissolved as 10 mM stock solution in DMSO.

Solutions injected at timepoint 1 have a final DMSO concentration of 1%. Solutions containing final concentrations of Resatorvid, Azeliragon, BCTC and control were measured to have <0.01 Endotoxin Units/ml by a Limulustext according to the European Medicines Agency ICH Guideline Q4B. Passing through a 0.22 µm filter is used to obtain sterile solutions as a final step for all solutions.

Study interruption and discontinuation

The study is interrupted or discontinued if it is believed to be contrary to the best interests of the subjects. In such a case, the reason for study interruption or discontinuation will be documented.

Concomitant medication

During the study, no concomitant medication is allowed to be taken by the subjects except for hormonal contraception. Necessity of anti inflammatory medication for apparently not study-related conditions after trial start leads to exclusion.

Randomization

The study design involves 10 groups. The low local dose, which does not spread to other test sites as well as the short duration of biological effects induced by pain, excludes biological carry over effects. To prevent bias from psychological carryover effects, multiples of 10

participants are tested using a Latin squares balanced design according to Williams (https://adsabs.harvard.edu/full/1949ausra...2..149w). For each participant, one of 10 pre-specified sequences is randomly selected and removed from the pool (Table 1).

	Injection #									
**Sequence #**	1	2	3	4	5	6	7	8	9	10
**1**	A	B	J	C	I	D	H	E	G	F
**2**	B	C	A	D	J	E	I	F	H	G
**3**	C	D	B	E	A	F	J	G	I	H
**4**	D	E	C	F	B	G	A	H	J	I
**5**	E	F	D	G	C	H	B	I	A	J
**6**	F	G	E	H	D	I	C	J	B	A
**7**	G	H	F	I	E	J	D	A	C	B
**8**	H	I	G	J	F	A	E	B	D	C
**9**	I	J	H	A	G	B	F	C	E	D
**10**	J	A	I	B	H	C	G	D	F	E

Table 1: Pre-specified sequences according to Latin squares balanced Williams design.

Blinding

The experimenter and the subject will be fully blinded for all injections.

Benefit risk assessment

In the course of this study only solutions containing non-hazardous pH values and only minute amounts of substances will be injected, thus, the potential associated risk is considered minimal. The study participants will have no apparent benefit from the study, why their effort will be financially compensated. Success of this study could lay the basis for subsequent studies elucidating mechanisms of inflammatory pain, thereby creating the foundation for novel analgesic therapeutic strategies. Therefore, the overall benefit outweighs the potential risk of this study.

Furthermore, the experimenter will constantly inspect the volunteer’s forearms during the experiment for potential conditions that require medical attention. Due to slow LPS redistribution, side effects of intravenous LPS injection as fever, headache, muscle ache are not to be expected. Nevertheless, volunteers will be instructed to report immediately to one of the contacts listed in the declaration of consent if any of these conditions occur after their visit.

In addition, we will contact the volunteers 72 h after the experiments and explicitly ask them for the appearance of any adverse events.

Statistical methodology and analysis

Primary outcome variable skin perfusion

The degree of hyperemia, quantified by laser speckle imaging, will be analyzed using ImageJ. The following steps describe the protocol for analyzing the (non-)inflamed spots of one forearm of a subject. To determine the volume under the surface (VUS, in analogy to an area under the curve, but for a 2-dimensional signal) of the flux change, for each of the previously injected skin spots a ROI covering the flow increase is adapted. The flux change (Δflux) is determined by subtracting from the average of the ROI the average signal of the surrounding forearm, and multiplying this with the ROI area. Any widespread or systemic change, which is not expected to occur, would be present in both the test site and the surrounding forearm and would therefore be corrected for by the chosen analysis approach. The analyzing person will be blinded concerning the type of injection. The variable is measured in arbitrary units (A.U.)

Secondary outcome variable Pain AUC

After completion of the pilot study acquired data were inspected graphically. The outcome variable for the proposed is based on pain ratings (0 = no pain, 100 = maximum imaginable pain). The Area Under the Curve (AUC) of pain is calculated over the time span from 90 to 110 seconds (last 20 seconds or last 5 pain ratings of the acid injection). This yields the outcome variable 'Pain AUC'.

Subjective pain ratings

The subjective pain ratings provided by the participant upon mechanical stimulation and increasingly acidic infusions are metric variables, noted on paper by the experimenter.

Sample size

To be able to answer the main research question with the experimental design, the question arises about the minimum multiple of 10 participants that provides sufficient power for all hypotheses of the main research question. The letters of the respective groups that each participant is injected with are shown in Figure 6 (Groups A-J).

Primary Hypothesis: The mean blood flow of LPS groups (D,E,F) is compared with the mean blood flow of LPS + Azeliragon groups (G,H). The sample size calculation using pilot study data yields a sample size of 10 participants for a relevant effect size of 35%, $\alpha = 0.05$ and $1 - \beta = 0.8$.

Secondary Hypothesis 1: The acidosis pain rating Pain AUC of LPS group E is compared with the Pain AUC of LPS + Azeliragon group H. The sample size calculation using pilot study data yields a sample size of 40 participants for a relevant effect size of 35%, $\alpha = 0.025$ and $1 - \beta = 0.8$.

Secondary Hypothesis 2: The mean mechanical pain rating (cross-pinching) of LPS groups (D,E,F) is compared with the mean mechanical pain rating of LPS + Azeliragon groups (G,H). The sample size calculation using pilot study data yields a sample size of 20 participants for a relevant effect size of 35%, $\alpha = 0.025$ and $1 - \beta = 0.8$.

Taking into account the inclusion of multiples of 10 participants due to the experimental design, 40 participants need to be enrolled to provide sufficient power for all hypotheses. Research questions 2 and 3 are purely exploratory and are not considered in the sample size calculation. Based on experience with previous studies we assume that up to 10% of participants might not be able to complete the study, e.g. due to unforeseen events such as illness. Therefore, we suggest for up to 45 subjects to be enrolled in the study.

Statistical analysis

Applicable to all analyses

Conditional residuals of each model will be inspected for gross deviations from normal distribution and overly influential observations. All reported p-values will be the results of two-sided tests. P-values ≤ 0.05 are considered statistically significant. Correction for multiple testing is not planned as there is only a single primary hypothesis. To stabilise the residual distributions in each analysis, appropriate transformations of the dependent variables are performed when necessary.

Analysis primary hypothesis

Descriptive statistics

The dependent variable blood flow (arbitrary units) will be described graphically. The first plot will be a spaghetti plot, with types of injections (A-J in Figure 6) on the x-axis, blood flow on the y-axis and a line for each subject.

The 10 injections A-J differ not only in whether LPS was initially injected, but also in whether acid or BCTC is subsequently injected. However, the neutral or acidic second injection is irrelevant for the primary analysis, as the blood flow is measured before the second injection. This results in repeated measurements within the test subjects: Injections A, B and C in Fig. 6

are identical with respect to the first injection; all do not contain LPS, D, E and F are also identical, they contain LPS, G and H are identical, they contain LPS and the RAGE antagonist, I and J are also identical, they contain LPS and the TLR4 antagonist. Based on that, a second descriptive graph will be generated. The blood flow values from identical injections will be averaged within each volunteer. This will result in a second spaghetti plot with the following categories on the x-axis: 1) Control (consisting of averages [arithmetic mean] within each participant of injections A, B and C), 2) LPS (D, E, F), 3) LPS with RAGE antagonist (G, H) and 4) LPS with TLR4-antagonist (I, J). Again, each volunteer will be represented by a line. Depending on the distribution, the arithmetic mean, geometric mean, or median will be plotted on top of the individual data.

Inferential statistics

The data will be analyzed by a linear mixed model. A factor with a level for each volunteer will be used as random factor, a factor 'Injection type' with 4 levels as fixed factor. As the injections do not have a specific sequence (all of them are injected within a few minutes 4.5 hours prior to simultaneous blood flow measurements), a period effect does not need to be incorporated. The primary hypothesis will be tested using a contrast between the two levels 'LPS' and 'LPS + RAGE antagonist' of the fixed factor. The estimated difference will be plotted with its 95% confidence interval, in case of prior transformations after back-transformation.

The other comparisons between the four levels of the fixed factors serve as positive controls: 1) No LPS vs. LPS estimates the blood flow increase due to LPS, which was already explored in the pilot study, 2) 'LPS' vs 'LPS + TLR4-antagonist' should be different, as it can be considered textbook knowledge that LPS causes inflammation via TLR4.

Analysis secondary hypothesis 1

Descriptive statistics

First, the subjective pain ratings will be plotted for each of the 10 injection types with the pain ratings (0 – 100) on the y-axis, the time during and after injections in seconds on the x axis, and a separate line for each volunteer. Each injection (A-J in Fig. 6) will be plotted in a separate panel. Next, Pain AUC, calculated from the pain ratings as described above, will be plotted by a graph with the 10 injections as categories on the x axis, the Pain AUC on the y axis, and separate lines for each volunteer. For each injection type, a suitable measure of central tendency will be plotted in addition to the individual Pain AUC values.

Inferential statistics

The data will be analyzed by a linear mixed model. The injection types A-J (Fig.6) will be levels of a fixed factor. Each volunteer will be represented by a level of a random factor. As injections are performed in a given sequence (Table 1), the period will be incorporated into the model as a fixed factor. Based on the lowest AIC, an AR(1), Toeplitz or compound symmetry covariance structure will be used.

The secondary hypothesis 1 will be tested by a contrast comparing injection E (LPS followed by acidic injection) with H (LPS+RAGE antagonist followed by acidic injection).

The following pairwise comparisons will additionally be performed, yet not corrected for multiple testing, and consequently interpreted as hypothesis generating:

- D vs. G, testing whether the RAGE antagonist affects mechanical pain after LPS due to injection of a non-acidic solution.
- E vs. J, testing whether the TLR4 antagonist affects acidosis pain after LPS due to injection of an acidic solution.
- D vs. I, testing whether the TLR4 antagonist affects mechanical pain after LPS due to injection of a non-acidic solution.

Analysis secondary hypothesis 2

Descriptive statistics

The dependent variable mechanical pain (0-100) will be described graphically. The first plot will be a spaghetti plot, with types of injections (A-J in Figure 6) on the x-axis, mechanical pain on the y-axis and a line for each subject. As the continuous acidic injections have not yet taken place at the time of the mechanical pain testing, injections A-C are identical (analogous to the blood flow), as are D-F, as well as G-H and I-J. Accordingly, the mechanical pain values of these injection combinations will be averaged (arithmetic mean) within each volunteer. These averaged values will be plotted as a spaghetti plot with the following categories on the x-axis: 1) Control (consisting of averages [arithmetic mean] within each participant of injections A, B and C), 2) LPS (D, E, F), 3) LPS with RAGE antagonist (G, H) and 4) LPS with TLR4-antagonist (I, J). On the y-axis the averaged mechanical pain values will be plotted, each volunteer will be represented by a line.

Inferential statistics

The data will be analyzed by a linear mixed model. A factor with a level for each volunteer will be used as random factor, a factor ‘Injection type’ with 4 levels as fixed factor.

The secondary hypothesis 2 will be tested using a contrast between the two levels 'LPS' and 'LPS + RAGE antagonist' of the fixed factor. The estimated difference will be plotted with its 95% confidence interval, in case of prior transformations after back-transformation.

The other comparisons between the four levels of the fixed factors serve as positive controls: 1) No LPS vs. LPS estimates the mechanical pain increase due to LPS (which was already explored in the pilot study), 2) 'LPS' vs 'LPS + TLR4-antagonist' should be different regarding mechanical pain.

Exploratory analysis research question 2

To address this interaction hypothesis, Pain AUC values from injections A (neutral, no LPS), B (acidic, no LPS), D (neutral, with LPS) and E (acidic, with LPS) will be analyzed.

The difference between A and B corresponds to the acidosis pain in native skin, since pain reported in A can only be mechanical.

The difference between D and E corresponds to the acidosis pain in inflamed skin, since pain reported in D can only be mechanical.

Whether these differences differ will be assessed by a mixed linear model using the interaction term of two binary fixed factors (LPS [with/without] and Acid [with/without]). Subjects will be included as levels of a random factor, the period as a fixed factor, the covariance structure will be determined based on the lowest AIC.

Exploratory analysis research question 3

To address this interaction hypothesis, Pain AUC values from acidic injections B (no LPS, no TRPV1 antagonist), C (no LPS, with TRPV1 antagonist), E (with LPS, no TRPV1 antagonist) and F (with LPS, with TRPV1 antagonist) will be analyzed.

The difference between B and C corresponds to the acidosis pain in native skin that is exclusively TRPV1-dependent.

The difference between E and F corresponds to the acidosis pain in inflamed skin that is exclusively TRPV1-dependent.

Whether these differences differ will be assessed by a mixed linear model using the interaction term of two binary fixed factors (LPS [with/without] and TRPV1-antagonist [with/without]). Subjects will be included as levels of a random factor, the period as a fixed factor, the covariance structure will be determined based on the lowest AIC.

For all analyses, a subanalysis is done to see if there are differences between female and male participants.

Ethical and legal aspects

Informed consent of subjects

Following comprehensive instruction regarding the nature, significance, impact and risks of this study, the subjects will give written consent to participate. In addition to the comprehensive instructions, the participants also receive a written information sheet in understandable language, explaining the nature and purpose of the study and its progress. The subjects have to agree to the possibility of study-related data being passed on to relevant authorities. During the instruction the participants are to be made aware of the fact that they can withdraw their consent without giving reasons at any time.

Privacy and data protection

All electronic records will only hold a sequentially assigned number and the sex for every participant, which does not allow to identify the participant. Sex is digitized to allow subanalysis by sex. Informed consent and handwritten records are stored in a locked cupboard in the applicant's office. This office is always locked when the applicant or the applicant's assistant is not present. Data will be handled in accordance with the DSGVO.

Reporting and publication

We plan to publish the data from this study. The manuscript will be circulated to all co-investigators before submission. Confidentiality of subjects in reports/publications are guaranteed.

Insurance

During their participation in this trial the volunteers are insured (Rahmenversicherung MedUni Wien, Polizzennummer 07229622-2). Details about the existing patient's insurance are provided in the patient information sheet.

Ethics and Good Clinical Practice (GCP)

The investigator ensures that this study is conducted in full conformance with the principles of the "Declaration of Helsinki" (as amended at the 64th WMA General Assembly, Fortaleza, Brazil, 2013) and with the laws and regulations of the country in which the research is conducted.

The investigator of the trial guarantees that only appropriately trained personnel will be involved in the study. All studies must follow the ICH GCP Guidelines and the regulatory requirements.

Therefore, this study follows the EU Directive embedded in the Austrian drug act.
