# Supplementary material for: RAGE mediates LPS-induced inflammatory pain in human skin – a double-blind, randomised, placebo-controlled full crossover study
Source: Nat Commun. 2026 Sep 2;17:10461. doi: 10.1038/s41467-026-76771-2 (PMC13635236; doi:10.1038/s41467-026-76771-2)
Supplement: Supplementary file 2 [file 41467_2026_76771_MOESM2_ESM.pdf]

nature portfolio

Corresponding author(s): Univ.-Prof. Dr. med. Michael J.M. Fischer

Last updated by author(s): Jun 11, 2026

Reporting Summary

Nature Portfolio wishes to improve the reproducibility of the work that we publish. This form provides structure for consistency and transparency in reporting. For further information on Nature Portfolio policies, see our Editorial Policies and the Editorial Policy Checklist.

Statistics

For all statistical analyses, confirm that the following items are present in the figure legend, table legend, main text, or Methods section.

n/a	Confirmed	
☐	☒	The exact sample size (*n*) for each experimental group/condition, given as a discrete number and unit of measurement
☐	☒	A statement on whether measurements were taken from distinct samples or whether the same sample was measured repeatedly
☐	☒	The statistical test(s) used AND whether they are one- or two-sided<br>*Only common tests should be described solely by name; describe more complex techniques in the Methods section.*
☐	☒	A description of all covariates tested
☐	☒	A description of any assumptions or corrections, such as tests of normality and adjustment for multiple comparisons
☐	☒	A full description of the statistical parameters including central tendency (e.g. means) or other basic estimates (e.g. regression coefficient) AND variation (e.g. standard deviation) or associated estimates of uncertainty (e.g. confidence intervals)
☐	☒	For null hypothesis testing, the test statistic (e.g. *F*, *t*, *r*) with confidence intervals, effect sizes, degrees of freedom and *P* value noted<br>*Give P values as exact values whenever suitable.*
☒	☐	For Bayesian analysis, information on the choice of priors and Markov chain Monte Carlo settings
☐	☒	For hierarchical and complex designs, identification of the appropriate level for tests and full reporting of outcomes
☐	☒	Estimates of effect sizes (e.g. Cohen's *d*, Pearson's *r*), indicating how they were calculated

Our web collection on statistics for biologists contains articles on many of the points above.

Software and code

Policy information about availability of computer code

Data collection	moorFLPI-2 Measurement V3.0; QST.Lab TCSII software version 14.40.17, Chromeleon 7.3.2
Data analysis	moorFLPI Review Software V6.0; Microsoft Excel 2016; R version 4.5.1, including nlme (version 1.3-168), mgcv (version 1.9-3) and emmeans (version 1.11.1) packages

For manuscripts utilizing custom algorithms or software that are central to the research but not yet described in published literature, software must be made available to editors and reviewers. We strongly encourage code deposition in a community repository (e.g. GitHub). See the Nature Portfolio guidelines for submitting code & software for further information.

Data

Policy information about availability of data

All manuscripts must include a data availability statement. This statement should provide the following information, where applicable:

- Accession codes, unique identifiers, or web links for publicly available datasets
- A description of any restrictions on data availability
- For clinical datasets or third party data, please ensure that the statement adheres to our policy

All data collected from subjects during the study, including sex, heat pain thresholds, pain ratings and blood flow values are provided in the Supplementary Information. For data protection purposes data has been de-identified and individual subjects are represented as numbers.

Research involving human participants, their data, or biological material

Policy information about studies with human participants or human data. See also policy information about sex, gender (identity/presentation), and sexual orientation and race, ethnicity and racism.

Reporting on sex and gender	Our study had equal numbers of female and male participants. Sex was added to the analysis as an exploratory factor.
Reporting on race, ethnicity, or other socially relevant groupings	Race, ethnicity, or other socially relevant groupings were not considered in our study.
Population characteristics	Age between 18 and 70 years was an explicit inclusion criterion for our study, median and range are reported, though not considered in the analysis.
Recruitment	Participants were recruited using a notice that was posted in publicly accessible buildings of the Medical University of Vienna.
Ethics oversight	Ethics Committee of the Medical University of Vienna

Note that full information on the approval of the study protocol must also be provided in the manuscript.

Field-specific reporting

Please select the one below that is the best fit for your research. If you are not sure, read the appropriate sections before making your selection.

[x] Life sciences [ ] Behavioural & social sciences [ ] Ecological, evolutionary & environmental sciences

For a reference copy of the document with all sections, see nature.com/documents/nr-reporting-summary-flat.pdf

Life sciences study design

All studies must disclose on these points even when the disclosure is negative.

Sample size	For initial estimation of the variance, 12 subjects were included in the pilot study. For the primary hypothesis of the main study, the mean blood flow of LPS groups (D,E,F) is compared with the mean blood flow of LPS + azeliragon injected spots (G,H). The sample size calculation using pilot study data yielded a sample size of 10 participants for a relevant effect size of 35%, $\alpha = 0.05$ and $1 - \beta = 0.8$. For the secondary hypothesis 1, the acid-induced pain rating AUC Pain of LPS and LPS + azeliragon is compared (E vs. H). The sample size calculation using pilot study data yielded a sample size of 40 participants for a relevant effect size of 35%, $\alpha = 0.025$ and $1 - \beta = 0.8$. For the secondary hypothesis 2, the mean mechanical pinch pain rating of LPS groups (D,E,F) is compared with the mean mechanical pinch pain rating of LPS + azeliragon groups (G,H). The sample size calculation using pilot study data yielded a sample size of 20 participants for a relevant effect size of 35%, $\alpha = 0.025$ and $1 - \beta = 0.8$.
Data exclusions	The per-protocol analysis did not allow for data exclusion.
Replication	The data gathered from blood flow measurements and pain models were highly reproducible throughout the study.
Randomization	For the main study, a Williams balanced latin square design was chosen to minimize psychological carryover effects between injections and to ensure complete counterbalancing of treatment sequences. Ten different injection types were compared, therefore this design required ten unique sequence combinations and a multiple of ten for the number of participants. For each participant, one of the pre-specified sequences was randomly selected and removed from the pool.
Blinding	For the main study, subject and experimenter were fully blinded regarding the specific allocation of sequences, which includes the presence of LPS, azeliragon and resatorvid or pH and BCTC in the respective solutions.

Reporting for specific materials, systems and methods

We require information from authors about some types of materials, experimental systems and methods used in many studies. Here, indicate whether each material, system or method listed is relevant to your study. If you are not sure if a list item applies to your research, read the appropriate section before selecting a response.

Materials & experimental systems

n/a	Involved in the study
☒	☐ Antibodies
☒	☐ Eukaryotic cell lines
☒	☐ Palaeontology and archaeology
☒	☐ Animals and other organisms
☐	☒ Clinical data
☒	☐ Dual use research of concern
☒	☐ Plants

Methods

n/a	Involved in the study
☒	☐ ChIP-seq
☒	☐ Flow cytometry
☒	☐ MRI-based neuroimaging

Clinical data

Policy information about clinical studies

All manuscripts should comply with the ICMJE guidelines for publication of clinical research and a completed CONSORT checklist must be included with all submissions.

Clinical trial registration

NCT07092215

Study protocol

The study protocol is accessible via the Supplementary Information.

Data collection

Data was collected at the Medical University of Vienna between 20th of November and 21st of December 2023 (pilot study) and between 17th of June and 11th of July 2025 (main study).

Outcomes

Primary outcome 'Δblood flow':

The image was binarized to generate regions of interest marking inflamed skin spots by three 'erode', followed by eight 'dilate' operations. Surrounding reference regions were generated by ten further 'dilate' operations. Pixel areas and mean grey values were then used to calculate a reference-subtracted volume integral ('Δblood flow') for every skin spot.

Secondary outcome 'AUC Pain':

Analyses involving injection-induced pain used the area under the curve of the pain ratings as a summary outcome. For each subject and injection type, pain was recorded on a 0–100 numeric rating scale at 5 s intervals until six consecutive 0 ratings were obtained. To obtain a standardised quantitative measure of the experienced pain, the area under the curve was calculated over the predefined inclusive interval 90–110 s, corresponding to the last 20 s of the injection. Within each subject and injection type, all pain ratings within this window were numerically integrated using the trapezoidal rule based on pain (0–100) and time (seconds), resulting in one area under the curve value for each injection type per subject. This value served as the outcome variable area under the curve of pain ('AUC Pain') with the unit pain-seconds.

Plants

Seed stocks

Report on the source of all seed stocks or other plant material used. If applicable, state the seed stock centre and catalogue number. If plant specimens were collected from the field, describe the collection location, date and sampling procedures.

Novel plant genotypes

Describe the methods by which all novel plant genotypes were produced. This includes those generated by transgenic approaches, gene editing, chemical/radiation-based mutagenesis and hybridization. For transgenic lines, describe the transformation method, the number of independent lines analyzed and the generation upon which experiments were performed. For gene-edited lines, describe the editor used, the endogenous sequence targeted for editing, the targeting guide RNA sequence (if applicable) and how the editor was applied.

Authentication

Describe any authentication procedures for each seed stock used or novel genotype generated. Describe any experiments used to assess the effect of a mutation and, where applicable, how potential secondary effects (e.g. second site T-DNA insertions, mosiacism, off-target gene editing) were examined.
