# Supplementary material for: RAGE mediates LPS-induced inflammatory pain in human skin – a double-blind, randomised, placebo-controlled full crossover study
Source: Nat Commun. 2026 Sep 2;17:10461. doi: 10.1038/s41467-026-76771-2 (PMC13635236; doi:10.1038/s41467-026-76771-2)
Supplement: Supplementary file 3 [file 41467_2026_76771_MOESM3_ESM.pdf]

nature portfolio

Peer Review File

RAGE mediates LPS-induced inflammatory pain in human skin

Corresponding Author: Professor Michael Fischer

This file contains all reviewer reports in order by version, followed by all author rebuttals in order by version.

Version 0:

Reviewer comments:

Reviewer #1

(Remarks to the Author)
The manuscript reports the development and validation of a novel human experimental pain model based on intradermal LPS administration to mimic localized bacterial inflammation, and uses this model to dissect underlying molecular mechanisms. In a pilot study, the authors characterized the time course of LPS-induced skin hyperemia and pain hypersensitivity, identifying a peak inflammatory response around 4.5–6 hours, with prolonged mechanical and acid-induced hypersensitivity. In a subsequent double-blind, randomized, placebo-controlled crossover study, they tested pharmacological antagonism of key candidate pathways. Co-injection of the RAGE antagonist azeliragon markedly reduced LPS-induced hyperemia and significantly attenuated both mechanical and acid-induced pain, whereas the TLR4 antagonist resatorvid had no detectable effect on any outcome. Using a refined intradermal acid ramp paradigm, the authors further demonstrate that inflammation shifts the pH sensitivity of pain toward near-physiological values, and that most acid-induced pain is mediated by TRPV1, as shown by inhibition with BCTC. Overall, the study identifies RAGE, rather than TLR4, as a dominant mediator of LPS-induced inflammatory pain in human skin and establishes a translationally relevant model for studying inflammatory pain mechanisms and therapeutic targets.

The study is well-designed and highly innovative. The study outcomes are very interesting, and provide important new insights into pain physiology. As such, the manuscript qualifies for publication in Nature Communications. However, in our view, the manuscript requires significant improvement before it is acceptable for publication:

General remarks:

1. The study design is complex but elegant. It must have been challenging for the authors to answer so many questions in one study. This is certainly an achievement, but also a flaw: even though we are highly familiar with both the applied LPS and pain methodology, it was still challenging for us to coherently follow the author's line of thinking. When reading the results section and studying the figures in detail, we could unravel and comprehend the study's objectives and main findings due to their high level of structure and clarity, but this was much less clear based on the text in the introduction and discussion section. In our opinion, the manuscript requires a thorough editorial upgrade to make sure that the complex and elegant study design is also understandable for readers less familiar with the matter. A more thoughtful separation between the main manuscript and supplement may be a solution to increase the readability and comprehension of the manuscript.

Furthermore, the manuscript also requires a solid quality check on language and grammar. The current quality is insufficient. There are also numerous examples of meaningless sentences (e.g. line 380-381: 'Unsurprisingly, TRPV1 still contributes, and this is still the majority.', or line 397-399, lines 401-404). These are just a couple of examples, but other cases can be found throughout the manuscript.

2. For a critical appraisal of the effects of the different antagonists on the challenge responses, it is important to understand whether the local antagonist exposure was sufficiently high enough. Based on the information provided, it is not clear whether this was the case, or whether there was a risk that a low or absent antagonist response could be attributed to underexposure. Substantial information is provided on pharmaceutical handling of the antagonists (which is thoroughly appreciated), but clear information on dose selection, route of administration, and estimated local exposure is missing (or obscured, in case we may have missed it). This information is very important in comprehending the study design and methodology. Please also indicate whether test products were GMP grade (and if not, how QP release was allowed), and

how the test products were acquired.

3. A key discussion point is the direct versus indirect activation of RAGE by LPS, but the mechanistic relationship between TLR4 and RAGE is hardly discussed in the context of the generated data, or in the context of literature. The authors state that it is unclear if direct binding of LPS to RAGE is a crucial step, or if RAGE is involved further downstream (lines 344-345). However, data also show that there was no contribution of TLR4 to hyperemia or hypersensitivity in the skin inflammation model (lines 351-352). Isn't this evidence that LPS drives RAGE directly?
Other observations that warrant more thorough discussion: The presented data show that the LPS-driven hyperemic response is RAGE-dependent. This is a very interesting finding, but the underlying mechanistic basis is hardly discussed. Another interesting observation is that LPS sensitization does not modulate pain responses to heat, whereas it does impact acid- or pinch-inflicted pain as shown by the pilot study. Please discuss these observations in greater detail, including relevant references.

Specific comments:

- (General) Consistency with US or UK English, e.g. sensitize/sensitise or honored/honoured (p.3 line 60-61).

- (General) Consider checking spelling and consistency throughout the manuscript, e.g. TRL4 vs TLR4 (p.3 line 64) or pinprick vs pin prick (p.24 line 404-405).

- (General) References exceed 70. Although guidelines are not strict as per the Nature Communications website, please consider whether all are necessary. As stressed earlier, the amount of information may be overwhelming for the reader.

- (General) Italicize bacterial genus, species and other latin wording (e.g. line 319-321).

- (General) Concentrations for Na2HPO4 and MgSO2 do not need to be written as solvated (with H2O behind it), as the concentration refers to solute only in solution.

- (General) LPS drives a hyperemic response, which is quantified by LSCI. However, we'd prefer to refer to this endpoint as skin perfusion rather than as hyperemia (by analogy with measuring body temperature rather than measuring fever).

- (General) Some skin areas were used for subsequent evaluations, by different methods. Are you sure that one intervention does not influence the response to another intervention performed thereafter on the same area. As example: heating the skin will impact the perfusion response at a later moment in time.

- (Introduction) Please include a solid section in the introduction explaining the physiological relationship between TLR4, RAGE and TRPV1.

- (Introduction) Objectives 2 and 3 appear to overlap. Please describe in greater detail to make the distinction.

- (Introduction) “pharmacological means” (line 81). Please describe in greater detail which means these are. Paragraph 2 mentions azeliragon and resatorvid, but does not include BCTC.

- (Results, line 187) 'RAGE is the primary mediator of mechanical sensitization by LPS'. This may be the case for cannula insertion-related pain, but for pinch/infusion pain there is still remaining response under RAGE inhibition. How do the authors then justify that RAGE is the primary mediator of mechanical sensitization?

- (Results, line 188) 'With self-closing forceps, a new mechanical pinch stimulus was established.' The actual meaning of this sentence in the presented context is unclear. Please either explain better, or limit this info to the materials and methods section.

- (Results, general) RAGE inhibition both modulates the hyperemic response to LPS and pain sensitization. Inter-individual variability for various endpoints is large. Did the authors study the correlation between (RAGE-dependent modulation of) hyperemic responses and hypersensitivity? If not, please do so.

- (Results, line 142/143) “pH21”, “levels16” please correct references.

- (Discussion) Consider a subheader for the limitations section in the discussion.

- (Discussion, lines 441-442) Intradermal administration is not considered a topical route of administration.

- (Discussion) Lines 326-331 are confusing. The authors compare inflammatory responses of the paws in rats with dermal hyperemia and pain in man. The relevance of this comparison is not clear: different species, anatomical location, different tissue and different underlying physiology make this a random comparison. Moreover, the authors state that mechanical hypersensitivity lags behind hyperemia, which is not evident based on the presented data. Also, the statement of a decoupling between acid hypersensitivity and hyperemia is far from evident, but this is a conclusion that the authors draw.

- (Discussion) The authors insufficiently discuss their own findings in the light of publicly available data. In many cases, literature is presented extensively, but the direct relevance for their findings is unclear. One good example of this are lines

332-350, presenting a lot of findings by other groups, without linking these findings to own data.

- (Discussion, lines 404-413) It is unclear how this text is supported by or relates to the generated data, or the problem setting as outlines in the Introduction. Also, the meaning/intention of lines 421-429 is difficult to establish.

- (Discussion, lines 484-486) Please provide data on LPS concentration-response as supplementary data.

- (Legends) Legends are included in some graphs (e.g. 5C), but not in all (e.g. 6E). Please consider legends for further clarity and uniformity.

- (Methods, line 593) Please correct typo ‘overserved’.

Reviewer #2

(Remarks to the Author)
This manuscript from Dr. Fischer’s group reports a refined intradermal LPS human skin inflammation paradigm, combining laser speckle hyperaemia, mechanical pinch and cannula-insertion pain, and a pH-ramp injection model, with local azeliragon (RAGE), resatorvid (TLR4), and BCTC (TRPV1) to dissect mechanisms of inflammatory hyperalgesia. The study is methodologically rigorous and yields internally consistent data indicating that, in this paradigm, hyperaemia and mechanical/acid hyperalgesia are largely RAGE-dependent, not detectably affected by TLR4 antagonism, and that TRPV1 continues to mediate most acid-evoked pain with a leftward shift of the pH–pain relationship in inflammation. Overall, this is a technically strong and mechanistically informative human study; with more cautious framing of the “new model” and the RAGE>TLR4 conclusion, and with additional support or discussion around target engagement and the TRPV1-independent component, it would make a valuable contribution to the human pain and inflammation literature. The following are some major suggestions (and one minor) that may help to improve the manuscript.

Major concerns:
1. Overstated “new model” claim Intradermal LPS as a local skin inflammation model is pre-existing; the genuine advance here lies in its refinement (laser speckle hyperaemia, pH ramp, mechanical pinch) and mechanistic pharmacology, rather than a de novo human model. The manuscript should be reframed accordingly and more clearly positioned relative to other human skin inflammation models (UVB, NGF, burn/freeze injury, prior intradermal LPS), with explicit discussion of what is shared and what is unique about this paradigm (e.g. strong mechanical and acid hyperalgesia, no heat hyperalgesia).

2. RAGE>TLR4: The conclusion that LPS-induced inflammation is RAGE- but not TLR4-dependent rests on an asymmetric pharmacological design, with a single TLR4 antagonist (resatorvid, 10 µM; total dose ~0.4 µg, ≈0.0002% of the daily s.c. dose used clinically) and no direct target-engagement readout, compared to azeliragon at 100 µM (total dose 5.3 µg, ≈0.1% of the daily oral phase III dose), where off-target effects are acknowledged but not experimentally ruled out. The authors should (1) narrow the scope of their claim to this specific intradermal LPS paradigm, dose, and time window, and (2) either provide *in vitro* data demonstrating that 10 µM resatorvid effectively blocks TLR4 signaling under matched conditions (e.g. human skin or immune cells exposed to the same LPS preparation). In addition, the rationale for using ~0.1% of the clinical azeliragon dose versus only ~0.0002% of the clinical resatorvid dose should be clarified (or the exposures more closely matched), as this discrepancy complicates a direct comparison of RAGE and TLR4 contributions in this model.

3. Incomplete sensory phenotype and limited time course: the model robustly produces mechanical and acid hyperalgesia, but no heat hyperalgesia, despite clear evidence of TRPV1 sensitization in the acid domain. Furthermore, the observation window (to 50 h) is sufficient to show resolution of hyperaemia but not of hypersensitivity, which clearly outlasts the vascular response; the authors themselves note that the time course of pain is not fully captured. Claims that the paradigm broadly “mimics local infection” should therefore be moderated; the model is best described as capturing selected acute/subacute features—particularly RAGE-linked mechanical and acid pain—rather than a comprehensive infection-related pain phenotype. If feasible, extending the observation window in at least a subset of subjects (e.g. to 72–96 h) for mechanical and pH-ramp readouts would substantially strengthen the characterization of the model’s temporal profile.

4. TRPV1-independent acid pain component remains mechanistically unresolved: the GAM-based analysis convincingly demonstrates both a TRPV1-independent component of acid pain and a leftward pH-pain shift in inflammation, with pain increases occurring at pH values where the inferred TRPV1-dependent component is minimal. However, the mechanism underlying this residual pain remains undefined. Negative data for ASIC and TRPA1 antagonism are drawn from previous naïve-skin experiments under different conditions and are not reproduced within the current inflamed paradigm. The authors should explicitly frame the residual component as mechanistically unresolved, avoid strong statements about which channels mediate it, and, if possible, either add exploratory data with ASIC/TRPA1 antagonists in the inflamed model or discuss why such experiments were not performed and which alternative proton sensors they consider most plausible.

Minor point:
Mechanical pinch reliability: the intraclass correlation within a given site for mechanical pinch ratings is modest (ICC ~0.29), indicating considerable measurement noise at the spot level, even though between-subject ICC is higher. A brief discussion of this in the main text, including its implications for precision and any considered refinements of the mechanical stimulus, would improve transparency.

Reviewer #3

(Remarks to the Author)
The authors have conducted an experimental study in healthy humans using intradermal lipopolysaccharide (LPS) injections as an inflammatory pain model to investigate related mechanisms of hypersensitivity to mechanical and acidic stimuli. To this end, they conducted a pilot study to establish and characterize the dose-time dependence of the LPS challenge model used for subsequent investigation of involved mechanisms. By using drug substances that were previously under clinical development antagonising RAGE and TLR4, they demonstrate that RAGE but not TLR4 reduces LPS-induced hyperaemia and related hypersensitivity to mechanical and acidic stimuli. The authors use an appropriate approach to adminster drug substances without the need to receive authorisation of a clinical study in humans as a clinical trial. Their findings are novel and interesting but methodological aspects need clarification to conclusively evaluate their findings and to be reproduced elsewhere.

A central part of their work is the use of LPS as a challenge agent. While the authors have used GMP-grade LPS that meets the required level of purity and stability for the use in humans, a dose for the mechanistic study was selected based on “ng” rather that endotoxin units (EU). As it is well known that the biologic activity of endotoxin mediates subsequent effects, it is of interest to have the relevant potency of the batch of endotoxin. Also, it is preferred to adjust the dose to endotoxin units rather than ng to have comparable results with other studies or future work. The nominal dose of one vial of LPS (1 µg or 1000 ng) can be considerably different from the effective dose in EU. This ambiguity needs specification and justification.

Furthermore, LPS is provided as lyophililized powder. However, LPS was stored in a stock solution of 1 mg/ml in ddH20 without further information on stability and sterility. Was this prepared daily? The authors need to provide detailed information on storage conditions and quality control to exclude potential instabilities or impurities with handling.

Co-injections of LPS with antagonists were performed. To this end, LPS was mixed with antagonists at respective concentrations. Did the authors check for stability of their compounds and LPS activity.

Also, resatorvid and azeliragon were used with indicated impurities at a relatively high local concentration (10µM for resatorvid and 100 µM for azeliragon). Off-target effects can not be excluded and might question specificity of the data. Could the authors provide justification or add this as a potential limitation.

The authors have preregistered their work which was executed according to the preregistered protocol. The clinical study has been executed according to established standards and essential information is provided.

Minor points for consideration

Results, Figure 1C: In the pilot study, hyperaemia has resided in all but one participant at 50 hours. Any explanation what happened here?

Results, Figure 1E+F: How can lack of pain by mechanical pinch and acid in one participant despite hyperaemia be explained and does this “phenotype” require exclusion in the main study?

Results, line 195: For mechanical pinch pain, intraclass correlation of 3 retest experiments was 0.29, indicating that the three pinch ratings at the same skin site were “moderately consistent” – is this really moderate or rather low?

Results, line 279: The authors state “None of the participants reported any adverse effects or taking anti-inflammatory drugs related to the study within 72 hours”. In other words, anti-inflammatory drugs not related to the study were taken? Please specifiy and rewrite to avoid ambiguity

Reference 75 seems to be a patent which is not cited correctly

Version 1:

Reviewer comments:

Reviewer #1

(Remarks to the Author)
We'd like to thank the authors for the thorough rebuttal. We think the updated manuscript now presents clear and important new information. We have read the manuscript with a lot of interest.

Reviewer #2

(Remarks to the Author)
The authors have addressed most of my concerns satisfactorily, and the manuscript is now substantially improved. The experimental approach is rigorous and the data are of high quality.

However, the central conclusion that LPS-induced inflammation is mediated by RAGE but not TLR4 still relies on indirect

pharmacological evidence, without direct confirmation of target engagement under the experimental conditions. While the authors provide a rationale for dose selection, the absence of effect of resatorvid does not conclusively exclude a role for TLR4.

I therefore recommend that the authors further temper this conclusion and explicitly limit it to the specific experimental context and conditions tested.

Reviewer #3

(Remarks to the Author)
The authors have carefully and sufficiently responded to my comments / concerns and have provided an appropriate and comprehensive update of the manuscript. Hardly worth mentioning, one remaining comment: The authors might want to provide the patent number (WO2005000295A1) in citation 67 to avoid ambiguity and to comply with citation sytle.

Open Access This Peer Review File is licensed under a Creative Commons Attribution 4.0 International License, which permits use, sharing, adaptation, distribution and reproduction in any medium or format, as long as you give appropriate credit to the original author(s) and the source, provide a link to the Creative Commons license, and indicate if changes were made.
In cases where reviewers are anonymous, credit should be given to 'Anonymous Referee' and the source.
The images or other third party material in this Peer Review File are included in the article’s Creative Commons license, unless indicated otherwise in a credit line to the material. If material is not included in the article’s Creative Commons license and your intended use is not permitted by statutory regulation or exceeds the permitted use, you will need to obtain permission directly from the copyright holder.
To view a copy of this license, visit https://creativecommons.org/licenses/by/4.0/

Reviewer #1 (Remarks to the Author):

The manuscript reports the development and validation of a novel human experimental pain model based on intradermal LPS administration to mimic localized bacterial inflammation, and uses this model to dissect underlying molecular mechanisms. In a pilot study, the authors characterized the time course of LPS-induced skin hyperemia and pain hypersensitivity, identifying a peak inflammatory response around 4.5–6 hours, with prolonged mechanical and acid-induced hypersensitivity. In a subsequent double-blind, randomized, placebo-controlled crossover study, they tested pharmacological antagonism of key candidate pathways. Co-injection of the RAGE antagonist azeliragon markedly reduced LPS-induced hyperemia and significantly attenuated both mechanical and acid-induced pain, whereas the TLR4 antagonist resatorvid had no detectable effect on any outcome. Using a refined intradermal acid ramp paradigm, the authors further demonstrate that inflammation shifts the pH sensitivity of pain toward near-physiological values, and that most acid-induced pain is mediated by TRPV1, as shown by inhibition with BCTC. Overall, the study identifies RAGE, rather than TLR4, as a dominant mediator of LPS-induced inflammatory pain in human skin and establishes a translationally relevant model for studying inflammatory pain mechanisms and therapeutic targets.

The study is well-designed and highly innovative. The study outcomes are very interesting, and provide important new insights into pain physiology. As such, the manuscript qualifies for publication in Nature Communications. However, in our view, the manuscript requires significant improvement before it is acceptable for publication:

General remarks:

1. The study design is complex but elegant. It must have been challenging for the authors to answer so many questions in one study. This is certainly an achievement, but also a flaw: even though we are highly familiar with both the applied LPS and pain methodology, it was still challenging for us to coherently follow the author’s line of thinking. When reading the results section and **studying the figures in detail, we could unravel and comprehend the study's objectives** and main findings due to their high level of structure and clarity, **but this was much less clear based on the text in the introduction and discussion section**. In our opinion, the manuscript requires a thorough editorial upgrade to make sure that the complex and elegant study design is also understandable for readers less familiar with the matter. A more thoughtful separation between the main manuscript and supplement may be a solution to increase the readability and comprehension of the manuscript.

> **We would like to thank the reviewer for the positive feedback regarding the figures, but also the constructive criticism regarding the introduction. We have shortened the first part of the introduction, which is directly followed by the study rationale and objectives. Only thereafter, the background regarding the potentially involved receptors is laid out.**

Furthermore, the manuscript also requires a solid quality check on language and grammar. The current quality is insufficient. There are also numerous examples of meaningless sentences (e.g. line 380-381: 'Unsurprisingly, TRPV1 still contributes, and this is still the majority.', or line 397-399, lines 401-404). These are just a couple of examples, but other cases can be found throughout the manuscript.

> **The first two indicated sentences are important, but have been too condensed and incomplete.**
> **Line 380-381 has been expanded to: "The novel aspect of this study is that also in inflammatory conditions, acid-induced pain largely depends on TRPV1."**
> **Line 397-399 has been revised to "The observed level of increased pH sensitivity exceeds prior cellular data and reaches a level of pathophysiological relevance. Frequently occurring and mild reductions of pH can be expected to reach pH 7.2 and therefore activate nociceptors and contribute to pain in humans."**
> **Lines 401-404 contained less important information about mechanical testing. This has been condensed to "The standard approach of the quantitative sensory testing battery to assess suprathreshold mechanical pain uses pinpricks."**
> **The manuscript has been carefully reread and edited.**

2. For a critical appraisal of the effects of the different antagonists on the challenge responses, it is important to understand whether the local antagonist exposure was sufficiently high enough. Based on the information provided, it is not clear whether this was the case, or whether there was a risk that a low or absent antagonist response could be attributed to underexposure. Substantial information is provided on pharmaceutical handling of the antagonists (which is thoroughly appreciated), but clear information on dose selection, route of administration, and estimated local exposure is missing (or obscured, in case we may have missed it). This information is very important in comprehending the study design and methodology. Please also indicate whether test products were GMP grade (and if not, how QP release was allowed), and how the test products were acquired.

> **Substance preparation was guided by GMP principles, including documentation, qualified personnel, adequate facilities and equipment, and the quality control of the product. The latter includes the source of the products, measurement of purity (HPLC), control for the absence of endotoxin and sterile packaging. For experimental studies which do not fit into clinical trial categories, there is no Qualified Person. The approval is solely provided by the institutional ethic committee, which reviewed all materials, including the manufacturing, and has approved the study.**
> **More important than the formalities is the point regarding sufficient substance exposure. It is certainly not in the interest of the authors to obscure anything, which is in our view well documented by the already extensive methods section, which we are happy to extend wherever helpful for comprehension or reproduction.**
> **Regarding the period of local antagonist exposure, a final concern would be redistribution, particularly to exclude that as a reason for the lack of effect by**

resatorvid. Local redistribution is largely determined by the lipophilicity, as highly lipophilic molecules are sequestered into lipophilic reservoirs and are therefore not part of the moving aqueous phase. This leads to a long tissue residence with only a slow release from the dermis over an extended period. There are no respective data for resatorvid available. However, for pravastatin and salmeterol, which have both a similar molecular weight as resatorvid and an xlogP of 1.6 and 3.9, a fraction of 23% and 40% of the substance remained in rat skin after 24 h (Ito et al. 2012). Resatorvid has an xlogP of 3.0, therefore a large fraction of the substance should be expected to remain in the dermis within the 4.5 h until measurement.

The concentration of the substances is based on the reported IC50 values. This considered our previous assessment of differences between *in vitro* and *in vivo* IC50 of about 6.5-fold, determined for an TRPA1 antagonist in an identical intradermal application (Heber et al. 2019). We assume that local dilution, resulting from a local pressure gradient driving the initial convective redistribution, explains this difference. The chosen 100-fold IC50, considering up to 10-fold local dilution results in at least 10-fold IC50 (assuming a Hill coefficient of 1), translates into an at least 90% inhibition.

We further detailed the local administration, including that there was "aspiration to ensure that small blood vessels were not punctured" and that "The injection depth of about 1 mm from the skin surface allows a visual control of the needle tip."

3. A key discussion point is the direct versus indirect activation of RAGE by LPS, but the mechanistic relationship between TLR4 and RAGE is hardly discussed in the context of the generated data, or in the context of literature. The authors state that it is unclear if direct binding of LPS to RAGE is a crucial step, or if RAGE is involved further downstream (lines 344-345). However, data also show that there was no contribution of TLR4 to hyperemia or hypersensitivity in the skin inflammation model (lines 351-352). Isn't this evidence that LPS drives RAGE directly?

The relationship between TLR4 and RAGE has been extended in the introduction by two new paragraphs.

Our results show that inhibition of RAGE reduces both increased sensitisation and blood flow. This does not allow to differentiate between a direct binding of LPS to RAGE and an LPS binding to another target which obligatorily involves RAGE in its signalling cascade. However, this topic has been addressed by others, showing "direct LPS binding to RAGE by a surface plasmon resonance assay, a plate competition assay, and flow cytometry" (Yamamoto et al. 2011). We have revised the respective section, citing that "LPS binds directly to RAGE".

Other observations that warrant more thorough discussion: The presented data show that the LPS-driven hyperemic response is RAGE-dependent. This is a very interesting finding, but the underlying mechanistic basis is hardly discussed.

We have added an additional paragraph in this regard.

Another interesting observation is that LPS sensitization does not modulate pain responses to heat, whereas it does impact acid- or pinch-inflicted pain as shown by the pilot study. Please discuss these observations in greater detail, including relevant references.

Internal exploratory experiments should not be overinterpreted, but in these the heat sensitivity after LPS was also not changed in our recently published approach of intradermal injection of increasingly heated fluid. This is in line with the study data. Nevertheless, it is unclear why neither the expected change in the heat pain threshold nor the suprathreshold heat response was observed.

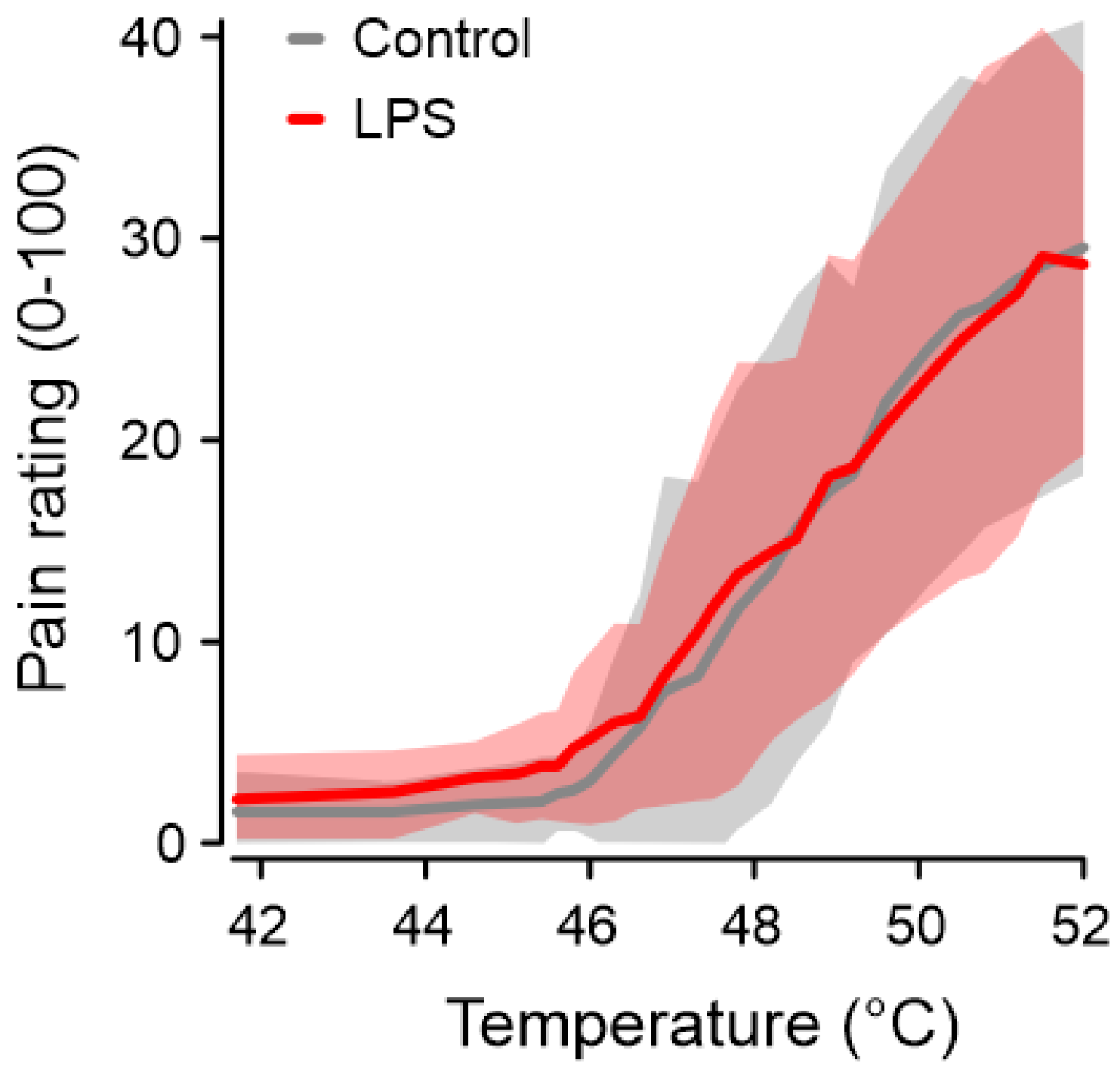

<u>Figure: Heat-induced pain in intradermal injection model is not sensitized by LPS.</u> At 4.5 h after injection of control or LPS, skin sites were tested by the increasingly heated intradermal injection (Heber et al., Sci Adv. 2024; 10(36):eado3498). There was no apparent change in the heat pain threshold or the suprathreshold pain ratings. Data are mean ± SEM from 11 injections tested double-blinded on three study authors.

We have revised the paragraph which discusses why acidic and heat sensitivity might differ, even if they are both at least partially mediated by TRPV1.

Specific comments:

- (General) Consistency with US or UK English, e.g. sensitize/sensitise or honored/honoured (p.3 line 60-61).

Thanks for pointing that out, the text has been changed to British English.

- (General) Consider checking spelling and consistency throughout the manuscript, e.g. TRL4 vs TLR4 (p.3 line 64) or pinprick vs pin prick (p.24 line 404-405).

Uniformity of these terms was corrected.

- (General) References exceed 70. Although guidelines are not strict as per the Nature Communications website, please consider whether all are necessary. As stressed earlier, the amount of information may be overwhelming for the reader.

We have considered all references and reduced these from 81 to 73, despite several new citations added in the revision.

- (General) Italicize bacterial genus, species and other latin wording (e.g. line 319-321).

Latin wording, particularly the bacterial genus and 'in vitro' has been italicised.

- (General) Concentrations for Na2HPO4 and MgSO2 do not need to be written as solvated (with H2O behind it), as the concentration refers to solute only in solution.

The H2O to is indeed unnecessary and has been removed.

- (General) LPS drives a hyperemic response, which is quantified by LSCI. However, we'd prefer to refer to this endpoint as skin perfusion rather than as hyperemia (by analogy with measuring body temperature rather than measuring fever).

The endpoint is indeed doppler shift, reflecting skin perfusion. We checked all occurrences. The result of increased blood flow is referred to as 'hyperaemia'. Wherever the text refers to the measurement of outcome variables, we changed it to "blood flow". Similar, when considering outcome variables, we changed 'pain hypersensitivity' to "pain sensitivity".

- (General) Some skin areas were used for subsequent evaluations, by different methods. Are you sure that one intervention does not influence the response to another intervention performed thereafter on the same area. As example: heating the skin will impact the perfusion response at a later moment in time.

This is an important point. The order of tests was carefully considered.
First, perfusion was acquired. This is a non-contact optical reading. Any painful stimulus should be considered likely to affect perfusion, but this could be avoided by acquiring it first.
Second, the order of painful stimuli was considered. Heat stimulation was applied next. The lack of a change in the heat pain threshold in the pilot study led to discontinuation of this stimulus in the main study, which allows us to answer this

question. The mechanical pinch sensitivity was not different, whether there was a prior assessment of the heat pain threshold or not:

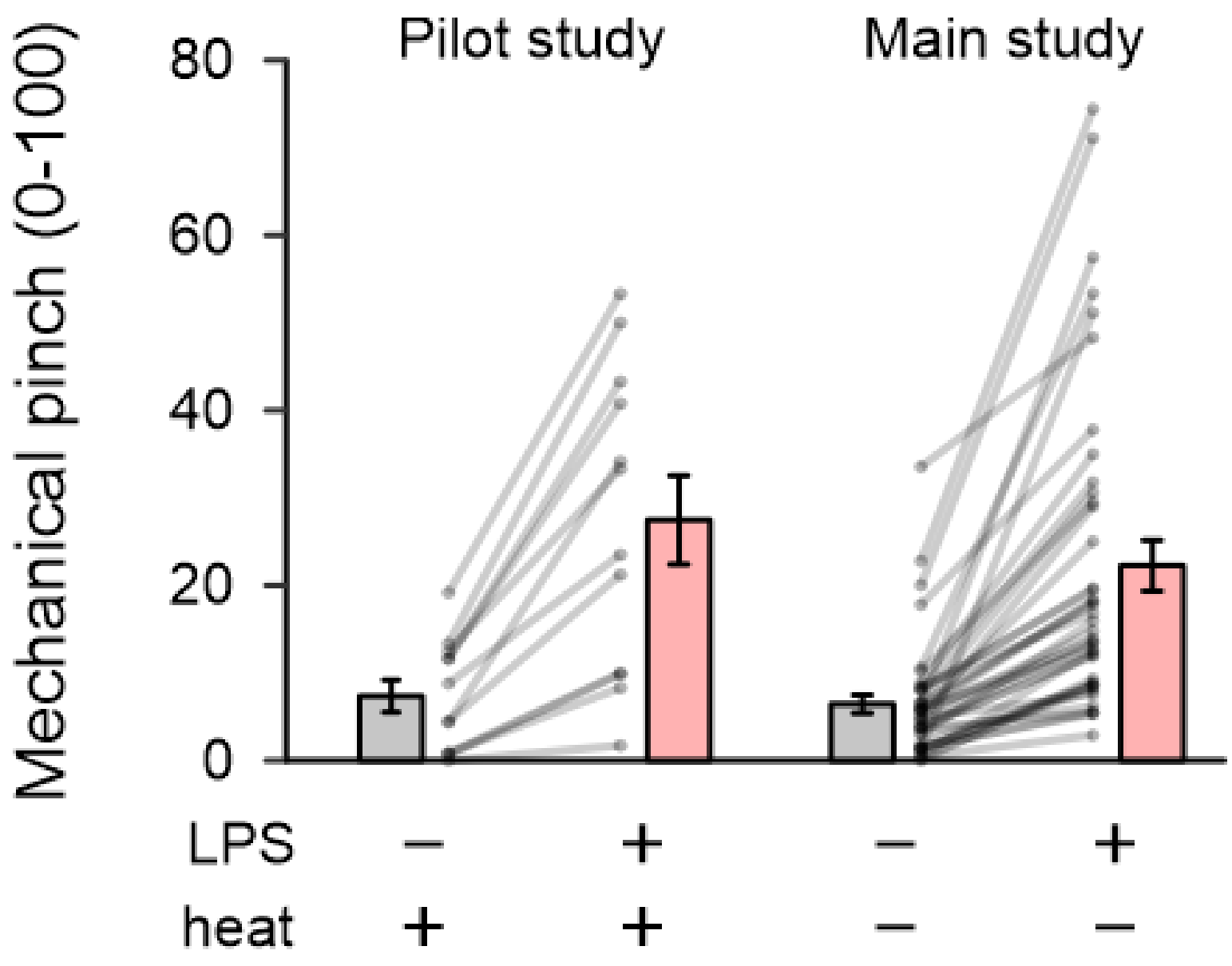

<u>Figure: Mechanical pinch ± prior heat pain threshold testing.</u> Skin spots were injected with control or LPS 4.5 h. In the pilot study, but not in the main study, the heat pain threshold was assessed before mechanical testing. There was no difference in reported pain induced by mechanical pinch between both study parts (full factorial mixed model with binary fixed factors 'prior heat' and 'LPS' and subject as random factor: main effect of 'prior heat' $p = 0.43$, main effect of LPS $p < 0.001$, interaction term $p = 0.35$), indicating that prior heat testing does not affect mechanical pinch pain. Individual ratings are provided as spaghetti plot. Data are mean ± SEM.

Third, prior assessment of heat pain threshold and mechanical pinch could affect acid sensitivity. This can be answered based on the internal control experiments with the authors as subjects (double-blinded). The acid stimulus is novel, and the optimisations were performed without heat and mechanical pinch beforehand, but with the full protocol for final validation. This allows direct comparison of acid-induced pain of 10 (+prestimulation) vs. 38 (-prestimulation) measurements for control spots and 17 (+prestimulation) vs 44 (-prestimulation) measurements in LPS-preinjected spots. These were performed on three of the authors, which were blinded regarding the protocol. The acid-induced pain was not different between experiments with and without heat pain threshold and mechanical pinch beforehand. Importantly, this holds true for LPS-preinjected and control skin spots.

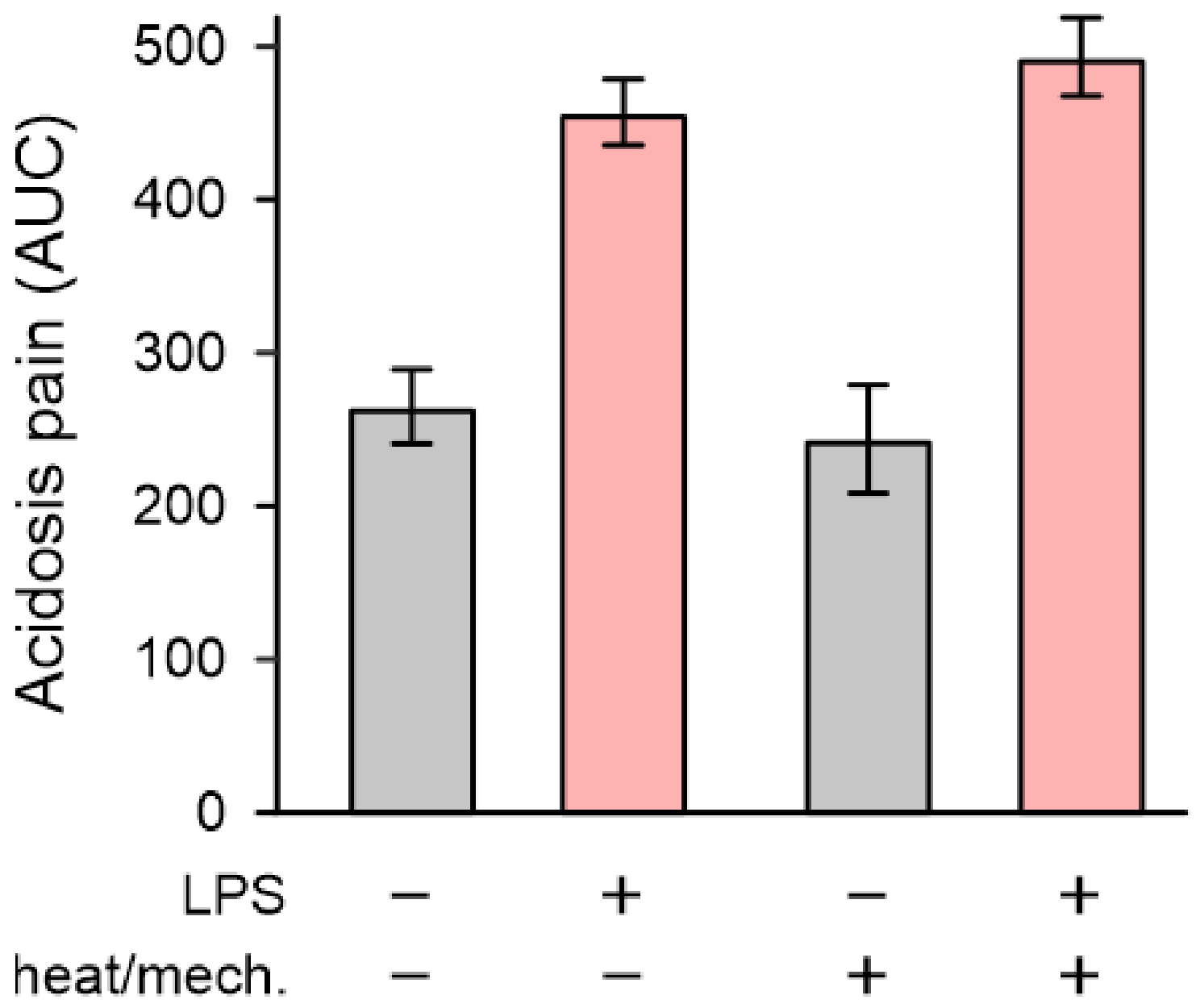

<u>Figure: Acid-induced pain ± prior heat and mechanical testing.</u> Skin spots were injected with control or LPS 4.5 h before heat pain threshold and mechanical pinch pain testing. There was no difference in acid-induced pain due to prior assessment of heat or mechanical sensitivity. Data are mean ± SEM.

- (Introduction) Please include a solid section in the introduction explaining the physiological relationship between TLR4, RAGE and TRPV1.

The mechanistic relationship of TLR4 and RAGE has been substantially extended in the introduction, as discussed above (point 3). Expanding the coverage of TRPV1 in the introduction might deter from the main findings concerning RAGE vs. TLR4.

- (Introduction) Objectives 2 and 3 appear to overlap. Please describe in greater detail to make the distinction.

The two objectives were prespecified before the study approval, and changing them post-hoc would violate clinical trial guidelines. However, we consider these "2) to elucidate the underlying pathway leading to inflammatory pain hypersensitivity and 3) to clarify the role of TRPV1 in acid-induced pain in LPS-based skin inflammation" quite different and can verbally explain how these were and should be understood.
Objective 2 is related to RAGE vs. TLR4 and was specifically addressed by their respective antagonists. Therefore, means to address objective 2 do not provide information about TRPV1. On the contrary, objective 3 is focussed on TRPV1 and further limited to its acid sensitivity. The only means to address this objective is BCTC, which cannot provide any information about RAGE vs. TLR4.

We clarified this in the last sentence of the introduction; we "address objectives 2 by application of RAGE and TLR4 antagonists and objective 3 by application of a TRPV1 antagonist".

- (Introduction) "pharmacological means" (line 81). Please describe in greater detail which means these are. Paragraph 2 mentions azeliragon and resatorvid, but does not include BCTC.

We added BCTC to this paragraph.

- (Results, line 187) 'RAGE is the primary mediator of mechanical sensitization by LPS'. This may be the case for cannula insertion-related pain, but for pinch/infusion pain there is still remaining response under RAGE inhibition. How do the authors then justify that RAGE is the primary mediator of mechanical sensitization?

We understand this criticism, as RAGE does not explain the majority of the effect. Given the unexpected lack of a TLR4 share, RAGE is the only so far known mediator. The heading has been changed to "RAGE mediates the mechanical sensitisation by LPS".

- (Results, line 188) 'With self-closing forceps, a new mechanical pinch stimulus was established.' The actual meaning of this sentence in the presented context is unclear. Please either explain better, or limit this info to the materials and methods section.

This was adapted to "With the chosen self-closing forceps, a new mechanical stimulus was established which is mildly painful in naive skin, but clearly sensitized in inflamed skin."

- (Results, general) RAGE inhibition both modulates the hyperemic response to LPS and pain sensitization. Inter-individual variability for various endpoints is large. Did the authors study the correlation between (RAGE-dependent modulation of) hyperemic responses and hypersensitivity? If not, please do so.

Correlations between the three outcome parameters had not been investigated and we thank the reviewer for this suggestion.

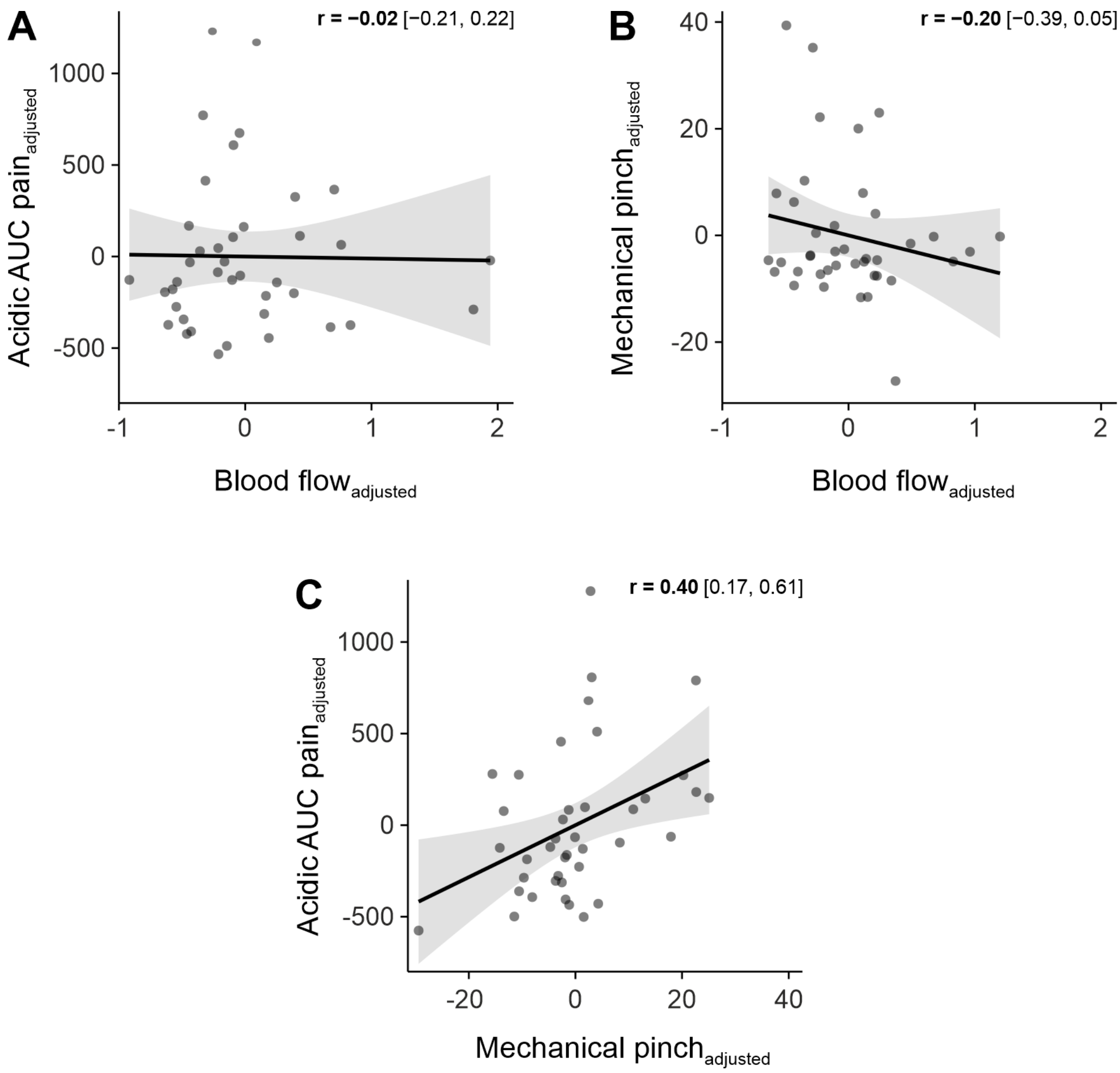

We added to the results: "Finally, associations of the primary and secondary outcome parameters were tested. There was no correlation between LPS-induced change in blood flow and mechanical sensitivity (partial Pearson r = -0.02, 95% CI [-0.21, 0.22]). There was also no correlation between LPS-induced change in blood flow and acid sensitivity mechanical sensitivity (partial Pearson r = -0.20, 95% CI [-0.39, 0.05]). To avoid false-positive correlation due to mediating parameters, data were adjusted (residualized) for acid-induced pain without LPS-induced inflammation or for mechanical pinch pain without LPS-induced inflammation, respectively. Individual subjective ratings to different painful stimuli should be expected to be correlated. To avoid that this results in a false-positive correlation, data were adjusted for both pain ratings provided at the skin sites without LPS-induced inflammation (mechanical pinch and acid-induced pain). Importantly, the adjusted LPS-induced mechanical and acid sensitivity were positively correlated (partial Pearson r = 0.40, 95% CI [0.17, 0.61]."

- (Results, line 142/143) “pH21”, “levels16” please correct references.

> **Thank you for spotting these mistakes. 21 and 16 were citations, but the superscript had been lost. These were again correctly inserted.**

- (Discussion) Consider a subheader for the limitations section in the discussion.

> **It is our style to have a separate paragraph for limitations, usually with the respective subheading. Nature communications author guidelines state “the Discussion should be succinct and may not contain subheadings”. As a compromise, there is a paragraph, starting with “Limitations:”**

- (Discussion, lines 441-442) Intradermal administration is not considered a topical route of administration.

> **Indeed, topical is medically used for applications onto the skins. To indicate the local exposure, we changed the wording to “local intradermal LPS challenge”.**

- (Discussion) Lines 326-331 are confusing. The authors compare inflammatory responses of the paws in rats with dermal hyperemia and pain in man. The relevance of this comparison is not clear: different species, anatomical location, different tissue and different underlying physiology make this a random comparison. Moreover, the authors state that mechanical hypersensitivity lags behind hyperemia, which is not evident based on the presented data. Also, the statement of a decoupling between acid hypersensitivity and hyperemia is far from evident, but this is a conclusion that the authors draw.

> **‘Decoupling’ was an overstatement. We downtoned this to “both mechanical and acid hypersensitivity recover slower than hyperaemia”.**

- (Discussion) The authors insufficiently discuss their own findings in the light of publicly available data. In many cases, literature is presented extensively, but the direct relevance for their findings is unclear. One good example of this are lines 332-350, presenting a lot of findings by other groups, without linking these findings to own data.

> **The respective section was shortened. It now only describes arguments for involvement of TLR4 and RAGE.**

- (Discussion, lines 404-413) It is unclear how this text is supported by or relates to the generated data, or the problem setting as outlined in the Introduction. Also, the meaning/intention of lines 421-429 is difficult to establish.

> **Lines 404-413 describe verbally the results of a large set of preliminary experiments. Personal discussions with the developers of the QST clarified that they also consider such a stimulus to be missing, and not reflected in the test battery. The text was revised, and now clearly separates ‘in own pilot experiments’**

from the description of study data, following "In the study, this mechanical pinch generated …"
Lines 421-429 were revised to "A TRPV1 contribution to the heat pain threshold could also be expected, due to the observed shift in TRPV1 sensitivity for acid detection. However, the pilot study surprisingly showed a lack of heat sensitisation in LPS-induced inflammation."

- (Discussion, lines 484-486) Please provide data on LPS concentration-response as supplementary data.

Providing a LPS dose-response relationship as supplementary data as part of the manuscript would require its own clinical trial, including approval by the ethics committee. Nonetheless, as stated in the indicated section in the methods ("A dose of 5 ng LPS per injection was validated in own pilot experiments, also e.g. 10-fold lower dose caused hyperaemia and hypersensitivity."), preliminary data are available. Below is the exploratory dose-dependency of LPS for blood flow and mechanical pinch.

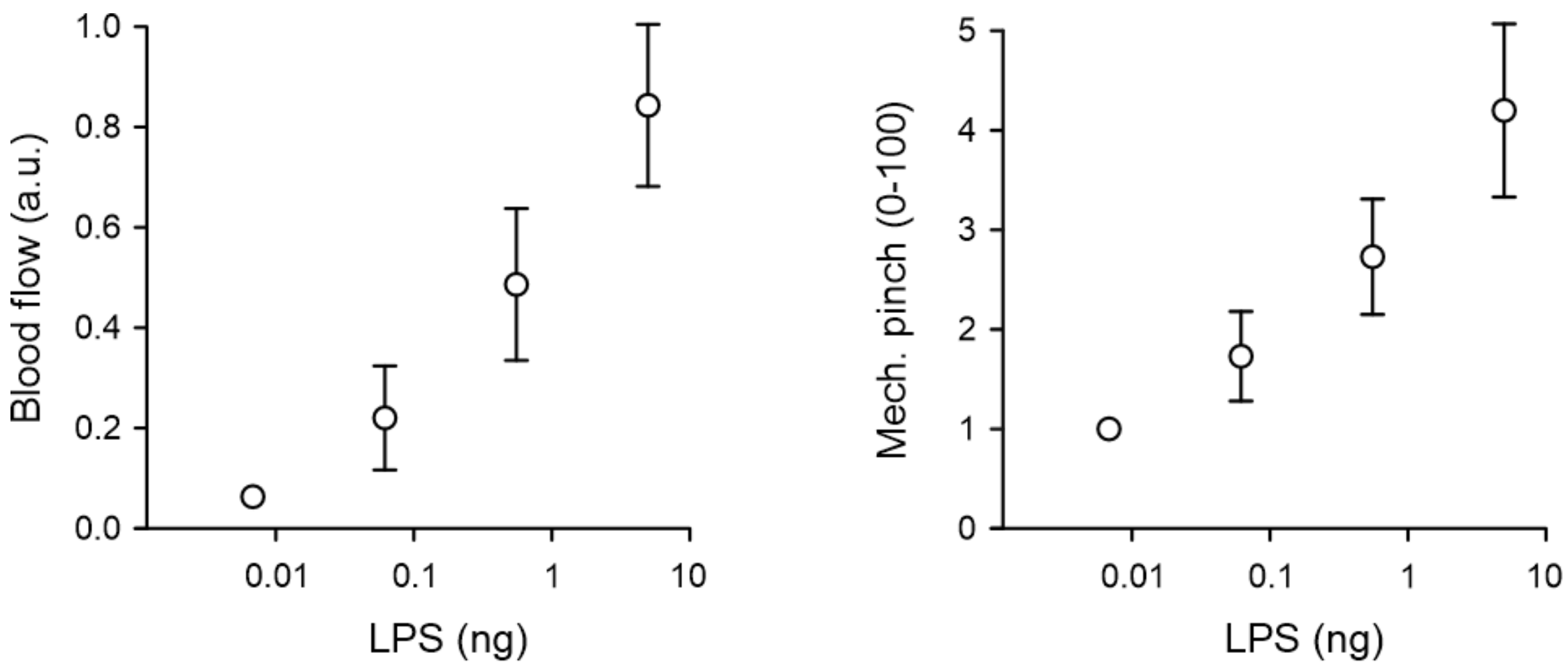

<u>Figure: LPS dose response for blood flow and mechanical pinch pain.</u> In the exploratory dose-response testing, lower doses generated a signal, but 5 ng was preferred due to signal amplitude and signal-noise ratio. Higher doses were not taken into consideration as this would have limited the number of LPS injections per subject. The data are presented as the mean ± SEM of five measurements per dose, performed on three study authors who were blinded to the LPS dose.

- (Legends) Legends are included in some graphs (e.g. 5C), but not in all (e.g. 6E). Please consider legends for further clarity and uniformity.

Labels have been added to figure 3H and 6E.

- (Methods, line 593) Please correct typo 'overserved'.

The typo has been corrected.

Reviewer #2 (Remarks to the Author):

This manuscript from Dr. Fischer's group reports a refined intradermal LPS human skin inflammation paradigm, combining laser speckle hyperaemia, mechanical pinch and cannula-insertion pain, and a pH-ramp injection model, with local azeliragon (RAGE), resatorvid (TLR4), and BCTC (TRPV1) to dissect mechanisms of inflammatory hyperalgesia. The study is methodologically rigorous and yields internally consistent data indicating that, in this paradigm, hyperaemia and mechanical/acid hyperalgesia are largely RAGE-dependent, not detectably affected by TLR4 antagonism, and that TRPV1 continues to mediate most acid-evoked pain with a leftward shift of the pH–pain relationship in inflammation. Overall, this is a technically strong and mechanistically informative human study; with more cautious framing of the "new model" and the RAGE>TLR4 conclusion, and with additional support or discussion around target engagement and the TRPV1-independent component, it would make a valuable contribution to the human pain and inflammation literature. The following are some major suggestions (and one minor) that may help to improve the manuscript.

Major concerns:
1. Overstated "new model" claim: Intradermal LPS as a local skin inflammation model is pre-existing; the genuine advance here lies in its refinement (laser speckle hyperaemia, pH ramp, mechanical pinch) and mechanistic pharmacology, rather than a de novo human model. The manuscript should be reframed accordingly and more clearly positioned relative to other human skin inflammation models (UVB, NGF, burn/freeze injury, prior intradermal LPS), with explicit discussion of what is shared and what is unique about this paradigm (e.g. strong mechanical and acid hyperalgesia, no heat hyperalgesia).

> **Indeed, intradermal LPS has been used by two other groups before, and the findings are discussed and cited. However, these did not assess sensitivity or pain. In all three occurrences of the word 'novel', this is always in the context of pain (Abstract: "established a novel human pain model", Objectives: "objectives of this study were 1) to establish LPS-based skin inflammation as a novel human pain model" and Conclusion "established a novel human pain model").**
>
> **To avoid that the 'pain component' is overread, the prior description of an intradermal LPS model was made more explicit "LPS has so far been applied intradermally in humans in four studies", directly citing all four papers.**

2. RAGE>TLR4: The conclusion that LPS-induced inflammation is RAGE- but not TLR4-dependent rests on an asymmetric pharmacological design, with a single TLR4 antagonist (resatorvid, 10 µM; total dose ~0.4 µg, ≈0.0002% of the daily s.c. dose used clinically) and no direct target-engagement readout, compared to azeliragon at 100 µM (total dose 5.3 µg, ≈0.1% of the daily oral phase III dose), where off-target effects are acknowledged but not experimentally ruled out. The authors should (1) narrow the scope of their claim to this specific intradermal LPS paradigm, dose, and time window, and (2) either provide *in vitro* data demonstrating that 10 µM resatorvid effectively blocks TLR4 signaling under matched

conditions (e.g. human skin or immune cells exposed to the same LPS preparation). In addition, the rationale for using ~0.1% of the clinical azeliragon dose versus only ~0.0002% of the clinical resatorvid dose should be clarified (or the exposures more closely matched), as this discrepancy complicates a direct comparison of RAGE and TLR4 contributions in this model.

The 0.0002% refer to the total dose continuously administered over 24 hours. To scrutinize a comparison of the local concentration co-applied with LPS to a clinical subcutaneous application, the redistribution from the subcutaneous injection spot, the distribution volume and the plasma half-life would need to be considered. For resatorvid, clinical pharmacokinetic parameters are unfortunately not available.
The comparison to the daily clinical doses is reported for risk assessment. The mentioned doses meet the EMA ICH M3(R2) guidelines and classify our study as a microdosing study, where the dose to be administered has to be below 100 µg and below 1% of the 'pharmacologically active dose'.
Chosen doses also had to be justified to the ethics committee. The drug doses used in our study were mainly based on receptor pharmacology. IC_{50} values were evaluated and multiplied by a factor of 100, given an expected 6.5-fold local dilution (Heber et al. 2019). The rationale was to aim for at least 10-fold IC_{50}, to obtain at least 90% inhibition, covering the targets largely.
Despite the large clinical doses administered to septic patients, resatorvid can be considered quite potent. The requested in vitro data have been reported more than 10 years ago, with reported IC_{50} values in the range of 37-310 nM for inhibiting responses in LPS-stimulated human immune cells (Kawamoto et al. 2008) and even lower IC_{50} values for inhibiting cytokine production (Masayuki et al. 2016). Consideration of the mentioned aspect explains the chosen dosing of resatorvid and also the 100 µM chosen for azeliragon, which had a reported IC_{50} of 1 µM.
As a rationale, we added to the methods section that the chosen concentrations are "(~100-fold IC_{50})".

3. Incomplete sensory phenotype and limited time course: the model robustly produces mechanical and acid hyperalgesia, but no heat hyperalgesia, despite clear evidence of TRPV1 sensitization in the acid domain. Furthermore, the observation window (to 50 h) is sufficient to show resolution of hyperaemia but not of hypersensitivity, which clearly outlasts the vascular response; the authors themselves note that the time course of pain is not fully captured. Claims that the paradigm broadly "mimics local infection" should therefore be moderated; the model is best described as capturing selected acute/subacute features—particularly RAGE-linked mechanical and acid pain—rather than a comprehensive infection-related pain phenotype. If feasible, extending the observation window in at least a subset of subjects (e.g. to 72–96 h) for mechanical and pH-ramp readouts would substantially strengthen the characterization of the model's temporal profile.

The time course of hypersensitivity is indeed incomplete. A new clinical trial to complete the model description, even assuming approval without revisions, would require about 6-12 months. We consider the importance of RAGE vs. TLR4 as the main result, therefore the expected delay in publication was avoided.

Revisiting immunological data from human skin punch biopsies by Buters et al. 2022, the determined cytokines IL-10, IL-1beta, IL6, IL8 and TNF returned to baseline at their latest time point of 48 h after LPS challenge, as did hyperaemia. However, immune cell presence was still elevated after 48 h, including neutrophils, monocytes, dendritic cells, and T- and B-lymphocytes. In hindsight, this might have suggested that present local immune cells might contribute to sensitivity (Tanaka et al. 2023).

4. TRPV1-independent acid pain component remains mechanistically unresolved: the GAM-based analysis convincingly demonstrates both a TRPV1-independent component of acid pain and a leftward pH-pain shift in inflammation, with pain increases occurring at pH values where the inferred TRPV1-dependent component is minimal. However, the mechanism underlying this residual pain remains undefined. Negative data for ASIC and TRPA1 antagonism are drawn from previous naïve-skin experiments under different conditions and are not reproduced within the current inflamed paradigm. The authors should explicitly frame the residual component as mechanistically unresolved, avoid strong statements about which channels mediate it, and, if possible, either add exploratory data with ASIC/TRPA1 antagonists in the inflamed model or discuss why such experiments were not performed and which alternative proton sensors they consider most plausible.

The residual component is mechanistically unresolved. We fully agree to this clear wording and have added ", which is mechanistically unresolved," to the respective section.
The TRPV1 participation was an important question, addressable alongside the TLR4 vs. RAGE objective. This brought the number of parallel groups to the practical limit. We added a paragraph, explaining why ASIC and TRPA1 were not addressed: "Other receptors, such as TRPA1 or ASICs, were not investigated in the present inflammation-focussed study as the number of injections per participant visit was already high. Including additional hypotheses would have increased the required sample size due to adjustment for multiple testing. A potential future study addressing the small TRPV1-independent fraction of acid-induced pain would require a large sample size."

Minor point:
Mechanical pinch reliability: the intraclass correlation within a given site for mechanical pinch ratings is modest (ICC ~0.29), indicating considerable measurement noise at the spot level, even though between-subject ICC is higher. A brief discussion of this in the main text, including its implications for precision and any considered refinements of the mechanical stimulus, would improve transparency.

In our opinion the arithmetic mean of a threefold measurement of mechanical pinch represents a good balance of minimal time for acquisition of the measurement and noise reduction by resampling. The mechanical pinch stimulus is only mildly painful, the resulting numerical ratings are low, and therefore a

small deviation is a high relative difference. The visualisation in Fig. S2A shows the expected high consistency of the measurements.

The intraclass correlation coefficient is an aggregate measure of multiple correlations. The ICC calculation considered variables differently than planned, resulting in the surprisingly low value. The ICC is not an easily understandable measure, and in addition has the flaw to ignore that variance is not uniform, but a function of the intensity of mechanical pain.

To further characterize the new mechanical pinch stimulus, we added panel C to Figure S2. We consider this helpful for further use of the stimulus, as it indicates the variance to be expected for a respective pain intensity.

Reviewer #3 (Remarks to the Author):

The authors have conducted an experimental study in healthy humans using intradermal lipopolysaccharide (LPS) injections as an inflammatory pain model to investigate related mechanisms of hypersensitivity to mechanical and acidic stimuli. To this end, they conducted a pilot study to establish and characterize the dose-time dependence of the LPS challenge model used for subsequent investigation of involved mechanisms. By using drug substances that were previously under clinical development antagonising RAGE and TLR4, they demonstrate that RAGE but not TLR4 reduces LPS-induced hyperaemia and related hypersensitivity to mechanical and acidic stimuli. The authors use an appropriate approach to adminster drug substances without the need to receive authorisation of a clinical study in humans as a clinical trial. Their findings are novel and interesting but methodological aspects need clarification to conclusively evaluate their findings and to be reproduced elsewhere.

A central part of their work is the use of LPS as a challenge agent. While the authors have used GMP-grade LPS that meets the required level of purity and stability for the use in humans, a dose for the mechanistic study was selected based on "ng" rather that endotoxin units (EU). As it is well known that the biologic activity of endotoxin mediates subsequent effects, it is of interest to have the relevant potency of the batch of endotoxin. Also, it is preferred to adjust the dose to endotoxin units rather than ng to have comparable results with other studies or future work. The nominal dose of one vial of LPS (1 µg or 1000 ng) can be considerably different from the effective dose in EU. This ambiguity needs specification and justification.

> **We agree that for many biological molecules, an activity determined per batch is better for comparison than the mass of the molecule.**
> **In the presented methodology and reporting we aligned with the prior studies by Buters et al. 2022a, Buters et al. 2022b (18 volunteers, 08-10/2018 and 24 volunteers, 10/2018 - 01/2019, respectively). Their lower dose of 5 ng was chosen as the larger dose of 15 ng had barely more effect, but would have caused a threefold LPS exposure, with overall exposure too close to clinical systemic endotoxin studies. To convey the identical dosing as described by Buters et al., we report this as previously.**
> **Triggered by the reviewer, we further scrutinized general reporting in this respect. Surprisingly, it is overall more common to report LPS dose than endotoxin units, ignoring the variable potency. We have added the manufacturer's information on endotoxin activity (10.3 EU/ng) to the methods.**

Furthermore, LPS is provided as lyophililized powder. However, LPS was stored in a stock solution of 1 mg/ml in ddH20 without further information on stability and sterility. Was this prepared daily? The authors need to provide detailed information on storage conditions and quality control to exclude potential instabilities or impurities with handling.
Co-injections of LPS with antagonists were performed. To this end, LPS was mixed with antagonists at respective concentrations. Did the authors check for stability of their compounds and LPS activity.

This is now further detailed in the methods "All solutions were prepared, sterile filtered and aliquoted in June 2023 and stored at -20°C until the experimentation day (pilot study in November 2023, main study in June 2025)".
Considering the objective readout hyperemia, there are no signs of a lower effectiveness in the main study compared to the pilot study 18 months earlier. This indirect assessment suggests a high level of stability. Also published data do not suggest a stability issue. LPS is rather stable: "Storage of LPS samples for a period of 1 year at 7°C had no effect on the endotoxin level." (Douwes et al., Appl Environ Microbiol. 1995; 61:1763-9).
The recombinant Factor C assay, done for this revision in February 2026 of the manuscript reported 5.5 EU/ng for the original sample diluted and frozen in June 2023. Since there is evidence for LPS stability, we consider for the difference between our and the manufacturers results a measurement error more likely than LPS degradation. The reply to reviewer 1 holds an LPS concentration-response curve. This demonstrates that even a twofold change in LPS would have little impact on the readouts of the study. Finally, all subjects were done within a short period of time, and every subject on the same day in one sitting. In case there was LPS degradation, it affects all subjects similarly, and also the LPS in every subject similarly. Therefore, even if there would have been some LPS degradation, this would not affect our conclusions. Further, addition of Azeliragon or Resatorvid to LPS did not lower the measured LPS activity in the recombinant Factor C assay.
There were still frozen diluted test substances, prepared for dropouts. These were retested by HPLC in February 2026. There were no signs of substance degradation. Importantly, the assay has the required sensitivity to detect potential breakdown products, as all marginal side peaks indicating impurities were identical to the measurements performed for quantifications before the study. We added "Substance stability was validated by HPLC in 2026".

Also, resatorvid and azeliragon were used with indicated impurities at a relatively high local concentration (10 µM for resatorvid and 100 µM for azeliragon). Off-target effects can not be excluded and might question specificity of the data. Could the authors provide justification or add this as a potential limitation.

Both substances were obtained in high purity and used at the lowest concentration required to largely inhibit the targets. These parameters were carefully considered beforehand: IC50 values were evaluated and multiplied by a factor of 100, given an expected 6.5-fold local dilution (Heber al. 2019), which applies also to impurities. The rationale was to aim for at least 10-fold IC50, to obtain at least 90% inhibition, covering this target largely.
Not only azeliragon and resatorvid (purities 96.7% and 98.3%) are locally diluted, but also the impurities. A factor of 6.5-fold is also used for these considerations. To provide an estimate of the potential impurities: A volume of 50 µl at a concentration of 100 µM contains 2.66 µg of azeliragon. All impurities together

have a mass of 0.088 µg. To evaluate the reviewers feedback, some further literature research was required:

Impurities consist of organic impurities, inorganic impurities, residual solvents, mutagenic impurities and stereochemical impurities. The International Council for Harmonisation guidelines Q3A-Q3D imposes limits on these in drugs, and these might be helpful in scrutinizing the exposure in the present study. The lowest limits in Q3D are the parenteral limits for cadmium, mercury and lead, being 2, 3 and 5 µg/day, respectively. Considering Q3C, the most restricted solvent is benzene with a permitted daily exposure of 20 µg. Finally mutagenic impurities are covered in guideline M7. To avoid mutagenicity of impurities, the latter have the lowest thresholds, with the minimum being 0.096 µg/day for N-Nitrosodimethylamine.

In summary, unless one assumes that the impurities consist solely of the most mutagenic substance possible, the applied intracutaneous microdosing approach should not be expected to have toxicity.

Regarding "Off-target effects can not be excluded": To consider a worst-case scenario, if all of the impurity was cadmium this would result in a concentration of about 2 µM. Sensitive structures are e.g. L-type voltage-gated calcium channels, where "with 2 µM Cd in the patch pipette, one can already begin to detect interruptions" (Lansman, J Gen Physiol. 1986; 88:321-47). In summary, a worst case scenario of impurities reaches the threshold of off-target effects. Assuming a more likely mix of impurities, off-target effects appear unlikely. We are grateful for having been pushed to consider this more carefully than before.

The authors have preregistered their work which was executed according to the preregistered protocol. The clinical study has been executed according to established standards and essential information is provided.

Minor points for consideration

Results, Figure 1C: In the pilot study, hyperaemia has resided in all but one participant at 50 hours. Any explanation what happened here?

The respective participant communicated mechanical impact by a volleyball at the test site after the -50 h LPS injection but before the -25 h LPS injection. Given that this does not violate the study protocol, despite the likely systematic error and the known cause thereof, guidelines forbid to exclude such a datapoint.

Results, Figure 1E+F: How can lack of pain by mechanical pinch and acid in one participant despite hyperaemia be explained and does this "phenotype" require exclusion in the main study?

Pain is subjective, hyperemia not. A subject with low sensitivity or a personality trait resulting in low pain reporting would explain this. In such subjects, it is reassuring that the parallel acquisition of hyperemia serves as positive control for LPS. There was no exclusion due to observed ratings. This topic was addressed in

the planning of the study. The advising biostatistician clearly argued against this, since this would bias the study sample, which in turn would not reflect the general population.

Results, line 195: For mechanical pinch pain, intraclass correlation of 3 retest experiments was 0.29, indicating that the three pinch ratings at the same skin site were "moderately consistent" – is this really moderate or rather low?

In our opinion the arithmetic mean of a threefold measurement of mechanical pinch represents a good balance of minimal time for acquisition of the measurement and noise reduction by resampling. The mechanical pinch stimulus is only mildly painful, the resulting numerical ratings are low, and therefore a small deviation is a high relative difference. The visualization in Fig. S2A shows the expected high consistency of the measurements.

The intraclass correlation coefficient is an aggregate measure of multiple correlations. The ICC calculation considered variables differently than planned, resulting in the surprisingly low value. The ICC is not an easily understandable measure, and in addition has the flaw to ignore that variance is not uniform, but a function of the intensity of mechanical pain.

To further characterize the new mechanical pinch stimulus, we added panel C to Figure S2. We consider this helpful for further use of the stimulus, as it indicates the variance to be expected for a respective pain intensity.

Results, line 279: The authors state "None of the participants reported". In other words, anti-inflammatory drugs not related to the study were taken? Please specify and rewrite to avoid ambiguity

The participants knew and signed that taking an inflammatory drug within the 50 h from the first LPS injection to the final measurement would lead to exclusion. None of the subjects reported taking an inflammatory drug during the study period.

The feedback received after 72 hours was poorly formulated and updated to: "All participants were contacted at 72 h after completion of the study and reported no adverse effects. They also reported not taking anti-inflammatory drugs."

Reference 75 seems to be a patent which is not cited correctly

This is indeed a patent, which reports the IC50 of azeliragon. In the patent, azeliragon is 'Example A', and the patent states "Example A inhibits binding with an IC50 of about 1 µM". Although this is a patent, this reflects the original information of the manufacturer, which most likely ran more tests on this than others.

These data are in line with recent data, which found an IC50 of 1.7 and 2.2 for the inhibition of cell line growth by azeliragon (Pomicter et al. 2025, so far only in abstract form).

Reviewer #2 (Remarks to the Author):

The authors have addressed most of my concerns satisfactorily, and the manuscript is now substantially improved. The experimental approach is rigorous and the data are of high quality.

However, the central conclusion that LPS-induced inflammation is mediated by RAGE but not TLR4 still relies on indirect pharmacological evidence, without direct confirmation of target engagement under the experimental conditions. While the authors provide a rationale for dose selection, the absence of effect of resatorvid does not conclusively exclude a role for TLR4.

I therefore recommend that the authors further temper this conclusion and explicitly limit it to the specific experimental context and conditions tested.

We thank the reviewer for constructive critique. Our approach is indeed only based on pharmacological evidence, and we were in the privileged position that antagonists for both targets have advanced sufficiently in clinical trials to test these in our study.

We added to the limitations section: "Target engagement in human subjects, e.g. by displacement of a radioligand in a microdosing study would be a valuable next step." This requires experience with such studies (which we admittedly don't have), is costly, and approval and conduction requires time. We believe that this merits a separate study and publication, and should be done by a group with respective experience.

Resatorvid is widely accepted as a specific TLR4 inhibitor, but we acknowledge that a lack of an observed effect cannot rule out TLR4 involvement across other contexts than the investigated skin inflammation. That this was observed in a 'skin inflammation model' is also stressed in the last sentence of the abstract. To prominently place it also at the end, we added "in the skin" into the final conclusion sentence.

In accordance with the reviewer's recommendation, we believe that the data speak for themselves. Therefore, claims should not overextend, which we tried to reflect in the text by stating that findings regarding the RAGE and TLR4 pathways might be limited to the investigated site. Placed prominently at the beginning of the limitations, we added: "It cannot be excluded that the relative importance of RAGE and TLR4 is different at another site."

Reviewer #3 (Remarks to the Author):

The authors have carefully and sufficiently responded to my comments / concerns and have provided an appropriate and comprehensive update of the manuscript. Hardly worth mentioning, one remaining comment: The authors might want to provide the patent number (WO2005000295A1) in citation 67 to avoid ambiguity and to comply with citation style.

The patent number has been added.

Editors’ requests:

Please note the reviewer also communicated with the editor that they wish for a statement of limitation to be made to include that the TRPV1-independent component of acid-induced pain is appropriately acknowledged but could be framed more explicitly as mechanistically unresolved.

This topic was addressed in the first revision by addition of the sentence: “However, the TRPV1-independent component remains mechanistically unresolved.” This was verbally now further stressed by: “However, there is a TRPV1-independent component, which remains mechanistically unresolved.”
